# Palladium-Catalyzed Synthesis of Ammonium Sulfinates from Aryl Halides and a Sulfur Dioxide Surrogate: A Gas- and Reductant-Free Process**

**DOI:** 10.1002/anie.201404527

**Published:** 2014-07-27

**Authors:** Edward J Emmett, Barry R Hayter, Michael C Willis

**Affiliations:** Department of Chemistry, University of Oxford, Chemistry Research LaboratoryMansfield Road, Oxford, OX1 3TA (UK); Oncology Innovative Medicines, AstraZeneca, Alderley Park, MacclesfieldCheshire, SK10 4TG (UK)

**Keywords:** alcohols, arenes, palladium, sulfonamides, synthetic methods

## Abstract

Sulfonyl-derived functional groups populate a broad range of useful molecules and materials, and despite a variety of preparative methods being available, processes which introduce the most basic sulfonyl building block, sulfur dioxide, using catalytic methods, are rare. Described herein is a simple reaction system consisting of the sulfur dioxide surrogate DABSO, triethylamine, and a palladium(0) catalyst for effective convertion of a broad range of aryl and heteroaryl halides into the corresponding ammonium sulfinates. Key features of this gas- and reductant-free reaction include the low loadings of palladium (1 mol %) and ligand (1.5 mol %) which can be employed, and the use of isopropyl alcohol as both a solvent and formal reductant. The ammonium sulfinate products are converted in situ into a variety of sulfonyl-containing functional groups, including sulfones, sulfonyl chlorides, and sulfonamides.

Sulfonyl-derived functional groups such as sulfones, sulfonamides, and sulfinic acids feature in a myriad of applications ranging from active ingredients in pharmaceuticals[1] and agrochemicals,[2] to key intermediates in complex chemical syntheses,[3] to speciality materials[4] and food additives.[5] The syntheses of these functional groups are in general based on classic reactivity patterns. For example, sulfones are most commonly prepared by the oxidation of a precursor sulfide, itself usually obtained from a conventional nucleophile/electrophile combination.[6] Aromatic sulfonamides are ordinarily accessed from the corresponding sulfonyl chlorides, which in turn are most often available from the parent aromatic using electrophilic aromatic substitution chemistry.[7] Both of these approaches feature significant limitations: the use of an oxidative route precludes the presence of oxidation-sensitive functional groups, and the corresponding sulfide precursors often require the use of foul-smelling thiols for their preparation. Using electrophilic aromatic substitution chemistry limits the accessible molecules to those that conform to the inherent electronic and steric factors which control selectivity in these processes. Alternative procedures exist for both of these functional groups;[8] however, convenient and general methods to access these important molecules are still lacking.

The fundamental feature that links all sulfonyl-derived functional groups is the -SO_2_- arrangement of atoms, thus suggesting that a conceptually simple route to these molecules is one that involves the direct catalytic introduction of SO_2_. Despite the ready availability of SO_2_ as a bulk feedstock, its use in transition-metal catalysis is severely limited. Pioneering reports from Pelzer and Keim established that the palladium-catalyzed introduction of SO_2_ was feasible, although these early reports of sulfinic acid synthesis described reactions of poor scope and required high pressures of SO_2_ and hydrogen, and the use of inconvenient aryl diazonium substrates [Eq. (1)].[9]

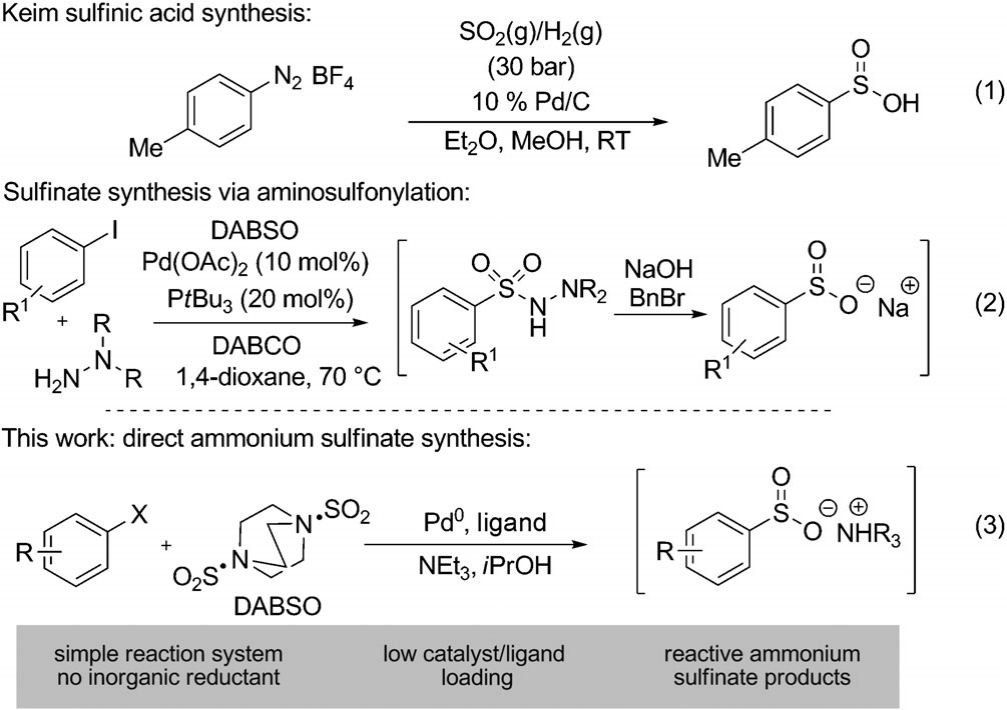


We have introduced the amine–SO_2_ charge-transfer complex DABSO [DABCO⋅(SO_2_)_2_] as a convenient and easy-to-handle solid which functions as a surrogate for SO_2_ gas in a number of known transformations,[10,11] and also in a new palladium-catalyzed aminosulfonylation procedure using aryl halides.[12] Wu et al. have reported related transformations employing K_2_S_2_O_5_ as the SO_2_ source,[13] and also the use of aryl boronic acids to access identical products.[14] All of these processes suffer from the limitation that only hydrazine nucleophiles can be employed.[15]

Rather than focus on the synthesis of a single class of sulfonyl derivative, a catalytic synthesis of sulfinates would potentially allow access to a host of sulfonyl-derived functional groups through established reactions.[16] Although we have recently shown that aminosulfonamides, prepared from the palladium-catalyzed combination of aryl halides, DABSO, and hydrazines, can be converted into the corresponding sulfinates by way of a base-promoted degradation, the process is cumbersome in that it requires the initial introduction and then expulsion of an expensive hydrazine derivative [Eq. (2)].[17] Shavnya and Miscitti have also shown that sulfinates can be prepared using palladium catalysis, in this case, from the combination of aryl halides and K_2_S_2_O_5_. However, the process requires high ligand loadings (30 mol %), the use of an excess of inorganic reductant, as well as a stoichiometric additive (TBAB).[18] Herein we describe an operationally simple protocol which employs only DABSO, triethylamine, and isopropyl alcohol, in combination with a low palladium and ligand loading to convert aryl halides into the corresponding ammonium sulfinates [Eq. (3)]. No inorganic reducing agent is needed, with isopropyl alcohol serving as both the reaction solvent and the formal reductant. The process is high yielding across a broad range of aryl and heteroaryl halides, and the ammonium sulfinate products display good reactivity in their conversion into a number of sulfonyl derivatives.

We selected the conversion of 4-iodotoluene (**1 a**) into the corresponding sulfinate (**2 a**) as a model transformation (Table 1). Our initial reagent and catalyst selections were based on our earlier developed aminosulfonylation chemistry, with the exchange of the hydrazine nucleophile for a range of reductants. The desired sulfinate was only observed in significant quantities when sodium formate in isopropyl alcohol was employed (entry 1).[19] The use of calcium formate provided a small improvement in efficiency (entries 2 and 3), and these conditions were then used to evaluate a number of alternative phosphine ligands (entries 4–9). Significant improvements in yields were obtained with both PCy_3_ and P*t*Bu_2_Me (used as their HBF_4_ salts), and the optimal ligand proved to be PdAd_2_Bu[20] (entry 9), which in addition to providing a small improvement in yield also delivered a practical advantage because of its improved stability.[20] We were aware that the *i*PrOH, used as solvent, could also function as a reductant in palladium catalysis,[21] and accordingly we then performed a duplicate experiment but without the addition of any external inorganic reducing agent. Pleasingly, an identical yield was obtained for the reductant-free transformation (entry 10). As far as we are aware, these experiments represent the first example of an alcohol being employed as a reductant in combination with a sulfinyl derivative. Finally, decreasing the palladium loading to 5 mol %, the ligand loading to 7.5 mol %, and the number of DABSO equivalents to 0.6 (1.2 equivalents of SO_2_) delivered the optimized reaction conditions (entry 11). The catalyst loading could be reduced further, for example, using only 0.5 mol % palladium and 0.75 % ligand led to a 91 % conversion into the sulfinate (entry 12).

**Table 1:** Optimization of reaction conditions for the formation of the sulfinate 2 a from 4-iodotoluene.[a] 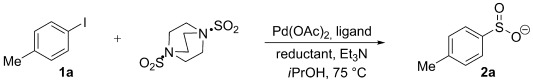

Entry	Ligand	Reductant	Yield [%][b]
1	P*t*Bu_3_⋅HBF_4_	HCO_2_Na	68
2	P*t*Bu_3_⋅HBF_4_	HCO_2_K	38
3	P*t*Bu_3_⋅HBF_4_	(HCO_2_)_2_Ca	71
4	binap	(HCO_2_)_2_Ca	23
5	dppf	(HCO_2_)_2_Ca	84
6	dccp⋅(HBF_4_)_2_	(HCO_2_)_2_Ca	25
7	PCy_3_⋅HBF_4_	(HCO_2_)_2_Ca	88
8	P*t*Bu_2_Me⋅HBF_4_	(HCO_2_)_2_Ca	95
9	PAd_2_Bu	(HCO_2_)_2_Ca	96
10	PAd_2_Bu	none	96
11[c]	PAd_2_Bu	none	99
12[d]	PAd_2_Bu	none	91

[a] Reaction conditions: Pd(OAc)_2_ (10 mol %), ligand (20 mol %), DABSO (1.1 equiv), Et_3_N (3.0 equiv), *i*PrOH [0.2 m], 75 °C, 16 h.

[b] HPLC yield relative to an internal standard.

[c] Pd(OAc)_2_ (5 mol %), PAd_2_Bu (7.5 mol %), DABSO (0.6 equiv).

[d] Pd(OAc)_2_ (0.5 mol %), PAd_2_Bu (0.75 mol %). binap=2,2′-bis(diphenylphosphanyl)-1,1′-binaphthyl, dccp=1,3-bis(dicyclohexylphosphino)propane, dppf=1,1′-bis(diphenylphosphino)ferrocene.

Sulfinates are versatile intermediates, but they are rarely the desired final product of a transformation. To evaluate the scope of the developed reaction we elected to derivatize the newly formed sulfinates in situ. Using α-bromo *tert*-butyl acetate as the electrophile allowed the corresponding sulfones to be readily prepared, and was preferable to attempting to isolate and purify individual ammonium sulfinate salts (Table 2). In general, a broad range of functionalized aryl iodides could be converted smoothly into the corresponding sulfinates, and then into the isolable sulfone products. Electron-rich substrates performed well, with a variety of electron-donating substituents being tolerated in all positions of the aromatic ring (**3 a**–**f**). Notable examples include the mixed sulfide-sulfone **3 d**, a product difficult to prepare using traditional sulfone methodology, and sulfones **3 e** and **3 f**, featuring unprotected OH and NH_2_ substituents, respectively. A selection of electron-neutral substrates were employed without incident (**3 g**–**j**). Electron-withdrawing functional groups were likewise compatible with the reaction conditions, with ester, ketone, amide, nitrile, and trifluoromethyl-substituted products being isolated in good yields (**3 k**–**o**). The greater reactivity of aryl iodide substrates allowed sulfones featuring bromo, chloro, and fluoro substituents to be prepared (**3 p**–**r**). Finally, although aryl iodide substrates proved to be most efficient, it was possible to employ aryl bromide starting materials, with the sulfones **3 k** and **3 o** being prepared from both aryl halide substrates.

**Table 2:** Scope of aryl halides in the palladium-catalyzed preparation of aryl ammonium sulfinates.[a]

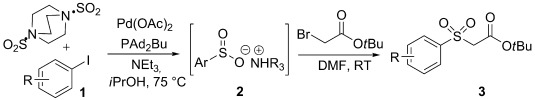
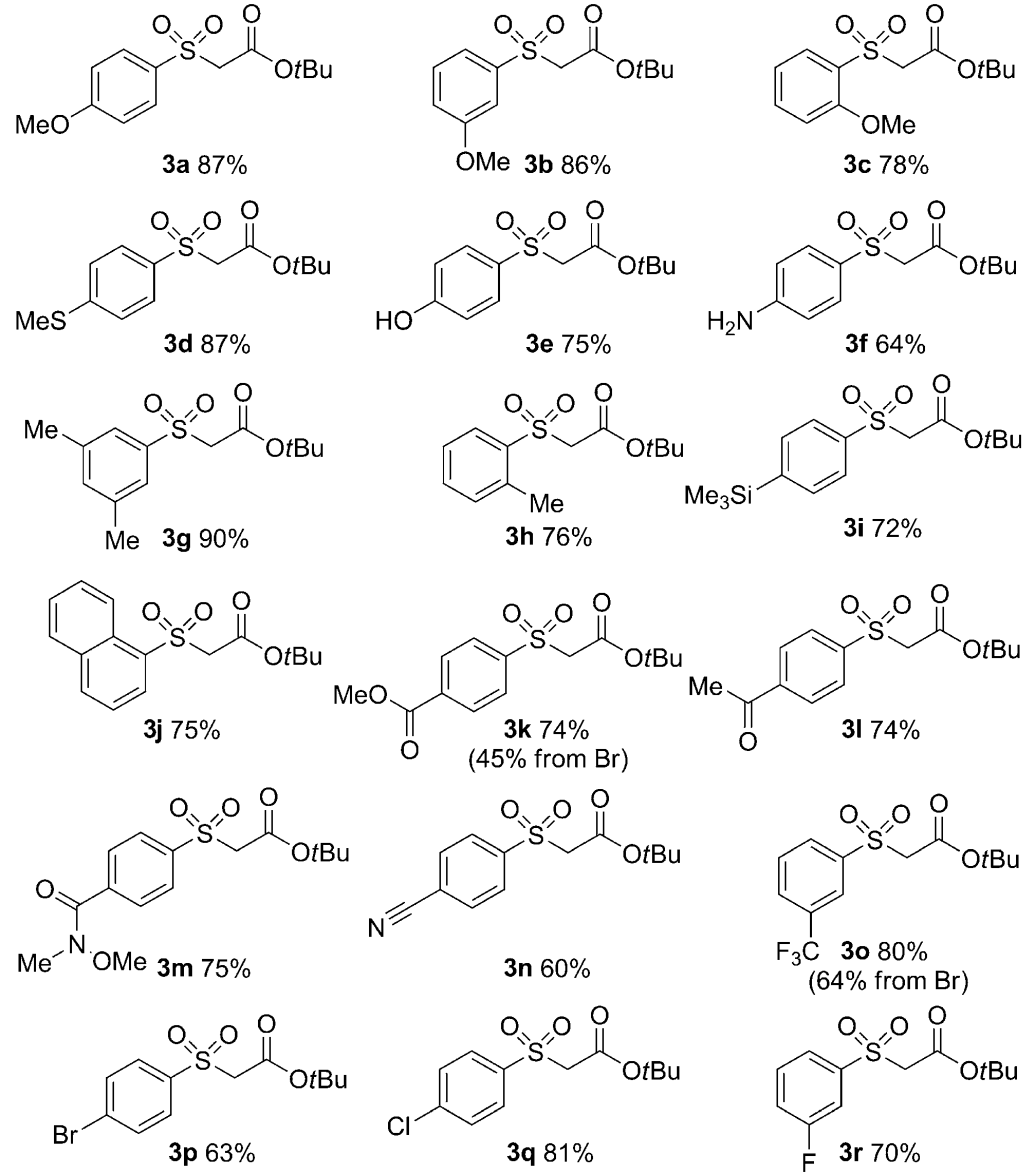

[a] Reaction conditions: Pd(OAc)_2_ (5 mol %), PAd_2_Bu (7.5 mol %), DABSO (0.6 equiv), Et_3_N (3.0 equiv), *i*PrOH [0.2 m], 75 °C, 16 h. DMF=*N*,*N*-dimethylformamide.

We next explored the use of the heteroaryl halides as substrates (Table 3). Examples of indole, benzofuran, benzothiophene, thiophene, pyridine, and quinolone ring systems were all converted into the expected sulfone products, although in general the yields were reduced relative to the benzene-derived examples (**3 s**–**x**). Several alkenyl iodides were also investigated, and proved to be compatible with the reaction (**3 y**,**z**).

**Table 3:** Heteroaryl and alkenyl halides in the palladium-catalyzed preparation of ammonium sulfinates.[a]

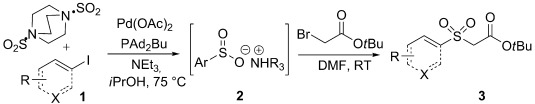
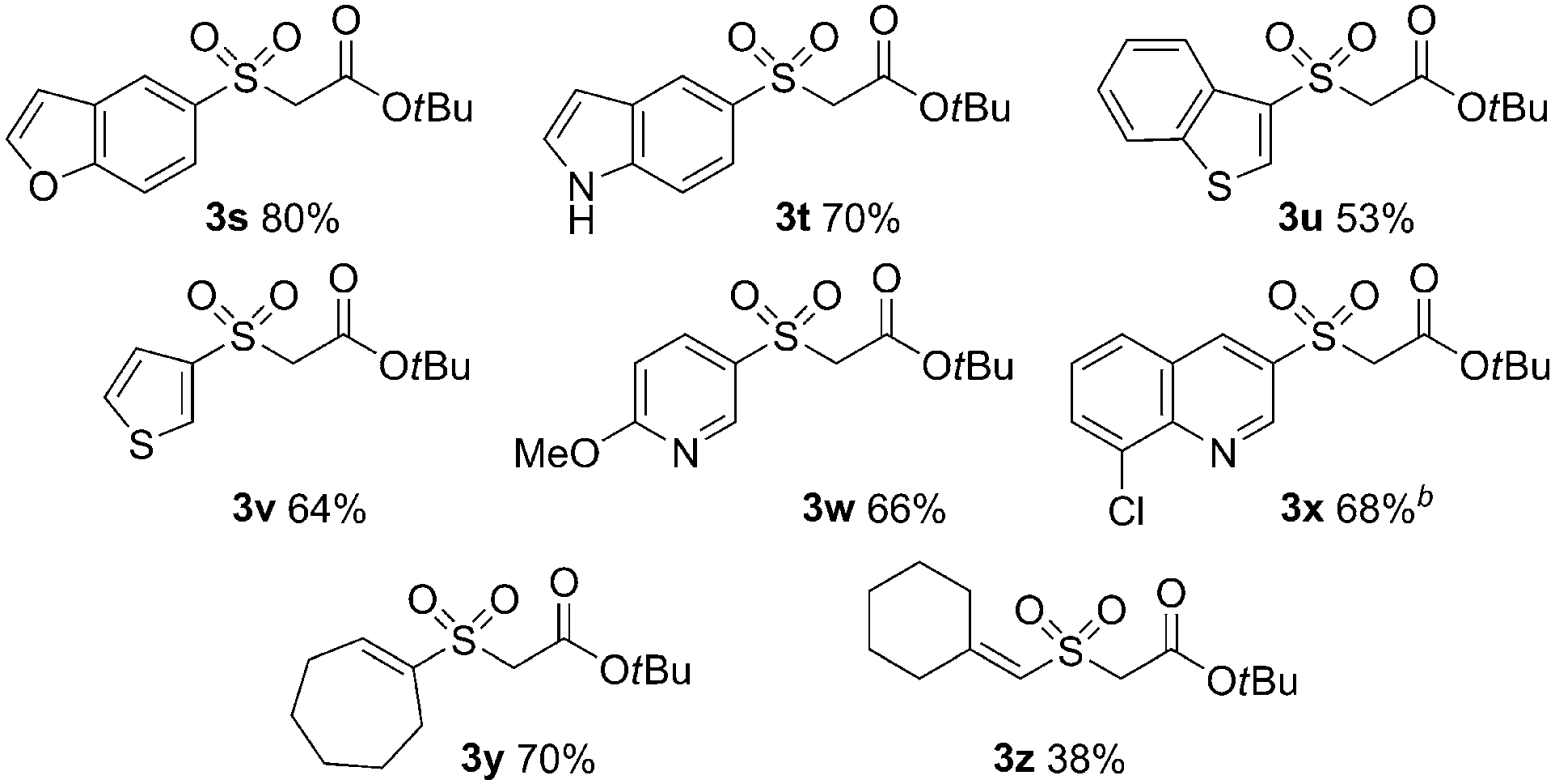

[a] Reaction conditions: Pd(OAc)_2_ (5 mol %), PAd_2_Bu (7.5 mol %), DABSO (0.6 equiv), Et_3_N (3.0 equiv), *i*PrOH [0.2 m], 75 °C, 16 h. [b] Pd(OAc)_2_ (10 mol %), PAd_2_Bu (15 mol %), DABSO (1.1 equiv).

For pragmatic reasons all of the reactions shown in Tables 2 and 3 were performed using 5 mol % of palladium and 7.5 mol % of ligand. On a preparative scale (12 mmol) it was possible to achieve good reactivity at lower loadings, with the sulfone **3 g** being isolated in 96 % yield from a reaction employing 1 mol % of palladium and 1.5 mol % of ligand (Scheme 1). Although we have not scaled the reaction further, the use of isopropyl alcohol as both the solvent and formal reductant holds promise in this perspective, as it has been identified as a sustainable solvent suitable for process development.[22]

**Scheme 1:**
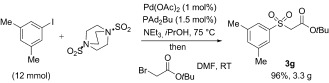
Preparative-scale synthesis of the sulfone 3 g.

At the outset of our investigation an efficient catalytic synthesis of sulfinates was targeted because of the wide range of established reactivity, for additional transformations, known for these molecules. In situ derivatization of sulfinates is particularly important as their purification is hampered because of their propensity to disproportionate in the presence of acid. However, the majority of these proven derivatization reactions had been developed on metal sulfinate salts. To validate our sulfinate synthesis we needed to ascertain the utility of the corresponding ammonium salts. Our initial findings, employing the ammonium sulfinate **2 g**, are shown in Scheme 2. Alkylation with a range of alkyl halides was possible and was achieved by adding a DMF solution of the electrophile to the crude sulfinate salt, and examples employing α-bromo *tert*-butyl acetate and benzyl bromide are shown (**3 g** and **4**). Cyclohexene oxide was efficiently opened to deliver the corresponding β-hydroxysulfone (**5**).[23] Diarylsulfones were accessible by a combination with aryl iodonium salts,[24] and treatment of **2 g** with diphenyliodonium chloride provided the diarylsulfone (**6**). Aryl(heteroaryl)sufones were available using an S_N_Ar-type process.[25] Accordingly, reaction of **2 g** with 2-chlorobenzothiazole provided the corresponding heterocyclic sulfone (**7**). Treatment of the ammonium sulfinate with sulfuryl chloride resulted in oxidative chlorination and allowed isolation of the derived sulfonyl chloride (**8**).[26] The moderate yield achieved for this transformation reflects isolation issues. The derived sulfonyl chloride intermediates can be combined directly with an amine to deliver sulfonamides.[26] For example, the morpholine derivative **9** was isolated in good yield. Finally, reduction using a Sn/HCl mixture allowed isolation of the corresponding disulfide (**10**).[27] In general, the ammonium sulfinate products were found to deliver reactivity at least comparable to that of their metal-salt counterparts, and in several cases to show increased rates of reaction, presumably because of their increased solubility in organic solvents.

**Scheme 2:**
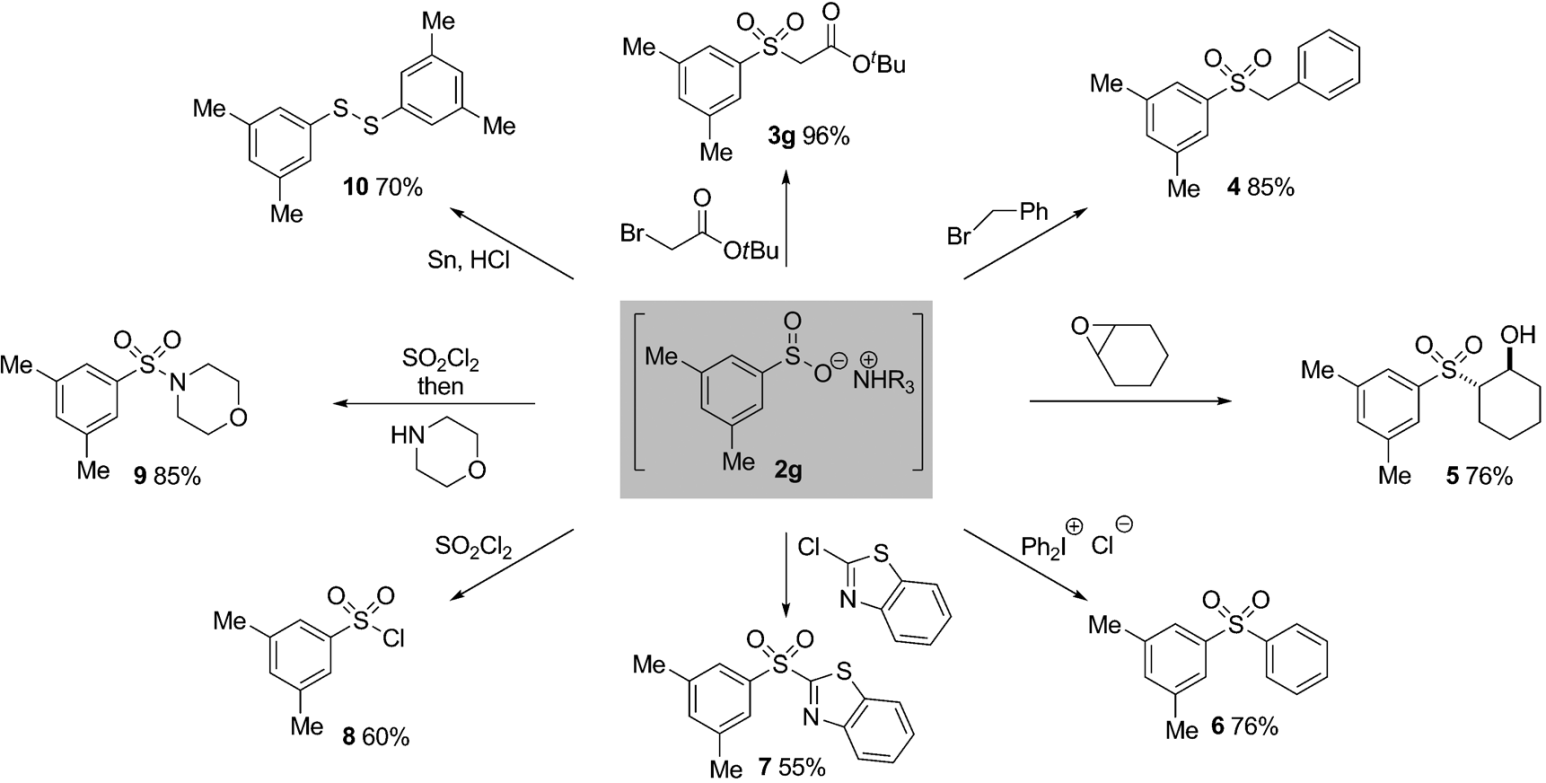
Additional reactivity of ammonium sulfinate 2 g.

In summary, by employing the SO_2_ surrogate DABSO, partnered with isopropyl alcohol as the reductant, we have been able to develop a simple and efficient process for the palladium-catalyzed conversion of aryl and heteroaryl halides into the corresponding ammonium sulfinates. This effective sulfination reaction employs low catalyst loadings, is operationally simple to perform, and has broad substrate scope. The ability to employ a simple alcohol as both reaction solvent and reductant significantly streamlines the process. The ammonium sulfinate products display good levels of reactivity and can be smoothly converted into a variety of sulfonyl derivatives. Further studies to develop related transformations, and to elucidate the mechanism of this transformation, are underway.
