# Supplementary material for: Palladium-Catalyzed Synthesis of Ammonium Sulfinates from Aryl Halides and a Sulfur Dioxide Surrogate: A Gas- and Reductant-Free Process
Source: Angew Chem Int Ed Engl. 2014 Jul 27;53(38):10204–8. doi: 10.1002/anie.201404527 (PMC4497614; doi:10.1002/anie.201404527)
Supplement: Supplementary file 1 [file anie0053-10204-sd1.pdf]

Angewandte Chemie
Eine Zeitschrift der Gesellschaft Deutscher Chemiker

Supporting Information

© Wiley-VCH 2014

69451 Weinheim, Germany

Palladium-Catalyzed Synthesis of Ammonium Sulfinates from Aryl Halides and a Sulfur Dioxide Surrogate: A Gas- and Reductant-Free Process**

*Edward J. Emmett, Barry R. Hayter, and Michael C. Willis**

anie_201404527_sm_miscellaneous_information.pdf

Supporting Information

1. Initial Screening of Optimization Conditions 2
2. Experimental 3
    2.1 General Considerations 3
    2.2 Data 4
3. References 20
4. Spectra 21

1. Initial Screening of Optimization Conditions

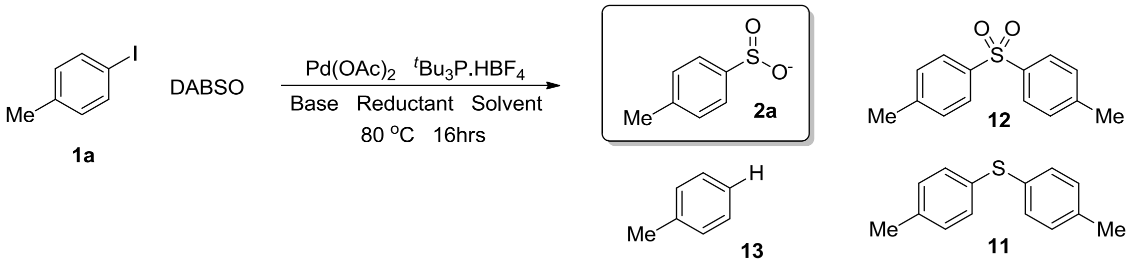

Entry	Base	Reductant	Solvent	2a:11	LCAP (12+13)
1	Et_3N	Oct_3SiH	Dioxane	0.9 : 1	6%
2	DABCO	Oct_3SiH	Dioxane	0.6 : 1	15%
3	DIPEA	Oct_3SiH	Dioxane	0.5 : 1	6%
4	Cs_2CO_3	Oct_3SiH	Dioxane	trace[b]	-
5	Et_3N	Et_3SiH	Dioxane	-[b]	-
6	Et_3N	$(EtO)_3SiH$	Dioxane	-[b]	-
7	Et_3N	PMHS	Dioxane	-[b]	-
8	Et_3N	$NH_4^+HCO_2^-$	Dioxane	-[b]	-
9	Et_3N	$Na^+HCO_2^-$	Dioxane	1.7 : 1	4%
10	Et_3N	$Na^+HCO_2^-$	NMP	trace[b]	-
11	Et_3N	$Na^+HCO_2^-$	Toluene	-[b]	-
12	Et_3N	$Na^+HCO_2^-$	^{t}BuOH	7.3 : 1	12%
13[c]	Et_3N	$Na^+HCO_2^-$	IPA[d]	8.4 : 1[e]	4%

[a] Reaction conditions: *p*-tolyl iodide (1 equiv., 0.3 mmol), DABSO (1.1 equiv.), $Pd(OAc)_2$ (10 mol%), $^tBu_3P.HBF_4$ (20 mol%), reductant (2.5 equiv.), base (3 equiv.), solvent [0.2M]. [b] Unreacted aryl iodide **1a** recovered. [c] 2.9 : 1 without reductant . [d] at 75 °C. [e] 68% HPLC yield of **2a** relative to internal standard (same entry as Table 1, entry 1 in main text)

LCAP = Liquid Chromatography Area Percentage

2. Experimental

2.1 General Considerations

Chemicals were purchased from Sigma Aldrich, Alfa Aesar, Fluorochem or Acros Organics and used without further purification. HPLC grade (or anhydrous grade where mentioned) solvents were purchased from Sigma Aldrich and used directly without further purification. Tetrahydrofuran, dichloromethane and acetonitrile were obtained anhydrous (KF titration < 10 ppm) from an in-house solvent drying system (Innovative Technology Inc. PS-400-7) having passed through anhydrous alumina columns. 'Petrol' refers to the fraction of light petroleum ether boiling in the range 40-60 ^{o}C.

Reactions were performed with continuous magnetic stirring, under an atmosphere of nitrogen, unless otherwise stated, using standard Schlenk techniques and all glassware was dried in an oven (>200 ^{o}C, overnight) and allowed to cool under a flow of dry nitrogen (passed through Drierite®) prior to use. Flash column chromatography was performed using Apollo scientific silica gel 60 (particle size 0.040-0.063 nm) with the indicated eluents. Thin Layer Chromatography (TLC) analysis was carried out on Merck Kieselgel 60 PF254 pre-coated aluminium backed sheets and visualised either by UV fluorescence (254 nm) and/or by staining with potassium permanganate ($KMnO_4$) or vanillin.

NMR spectra were recorded at ambient temperature on Brüker AVN 400 (400 MHz) or AVC 500 (500 MHz) spectrometers. Chemical shifts (δ) are reported in parts per million (ppm) and referenced relative to the residual solvent peak(s) ($CDCl_3$ @ 7.27 (^{1}H) or 77.0 (^{13}C) ppm). Coupling constants (J) are given in Hertz (Hz) and rounded to the nearest 0.5 Hz. Assignments were made on the basis of chemical shifts, coupling constants, COSY, HSQC and comparison with spectra of related compounds. Signal multiplicities are denoted as: s, singlet; d, doublet; t, triplet; q, quartet; m, multiplet; br., broad; app., apparent.

Melting points were measured using a Leica Gallen III hot-stage microscope. Low resolution mass spectra were recorded on a Fisons Platform spectrometer (ESI). High resolution mass spectra were measured by the internal service at the University of Oxford using a Bruker Daltronics microTOF spectrometer. *m/z* ratio values are reported in Daltons; high resolution values are calculated to four decimal places from the molecular formula, all found within a tolerance of 5 ppm. Infrared spectra were determined neat using a Bruker Tensor 27 FT spectrometer with an internal range of 600-4000 cm^{-1}.

The synthesis of DABSO (now commercially available from Sigma Aldrich and Key Organics) was previous reported in our laboratory.[1]

2.2 Data

(4-Iodophenyl)Trimethylsilane (1i)

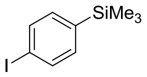

The procedure of Buchwald *et al.*[3] was used with the use of (4-bromophenyl)trimethylsilane[4] (0.23 g, 1.0 mmol) to give the titled *aryl iodide* **1i** as a colourless liquid (0.27 g, 98%); ^{1}H NMR (400 MHz, $CDCl_3$) δ 7.55 (2H, d, *J* = 8.0, Ar-*H*), 7.10 (2H, d, *J* = 8.0, Ar-*H*), 0.11 (9H, s, Si*Me*$_3$); ^{13}C (100 MHz, $CDCl_3$) δ 139.8, 136.8, 135.0, 95.7, -1.3; IR υ_{max} (neat)/cm^{-1} 2954, 1568, 1373, 1248, 1105, 1057, 1006; HRMS (EI) found *m/z* 260.9591 (100%, $[M-Me]^+$), 275.9821 (20%, $[M]^+$), $C_9H_{13}SiI^+$ (M^+) requires *m/z* 275.9826.

4-Iodo-*N*-methoxy-*N*-methylbenzamide (1m)

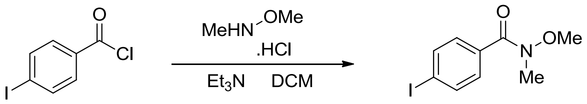

To a suspension of *N*,*O*-dimethylhyroxylamine hydrochloride (0.20 g, 2.1 mmol) in DCM (5 mL) cooled to 0 °C was added triethylamine (0.60 mL, 4.3 mmol) dropwise. A solution of 4-iodobenzoyl chloride (0.50 g, 1.9 mmol) in DCM (5 mL) was added dropwise at the same temperature before the resultant suspension was allowed to warm to room temperature and stirred for 1 hr until the acid chloride was consumed by TLC. The reaction mixture was quenched with $NaHCO_{3(aq)}$ (20 mL), the layers separated and the aqueous layer extracted with DCM (3 × 30 mL). The combined organic layers were dried ($MgSO_4$), filtered and concentrated *in vacuo*. Purification by flash column chromatography (10–50% EtOAc in petrol) gave the titled *amide* **1m** as a thick colourless oil (0.48 g, 88%); ^{1}H NMR (400 MHz, $CDCl_3$) δ 7.75 (2H, d, *J* = 8.5, Ar-*H*), 7.42 (2H, d, *J* = 8.5, Ar-*H*), 3.52 (3H, s, N*Me*), 3.34 (3H, s, O*Me*); ^{13}C (100 MHz, $CDCl_3$) δ 168.8, 137.1, 133.3, 129.9, 97.3, 61.1, 33.4; IR υ_{max} (neat)/cm^{-1} 2933, 1636 (C=O), 1584, 1414, 1376, 1212, 1181, 1110, 1065, 1007; LRMS (ESI) *m/z* 292 (35%, $[M+H]^+$), 314 (10%, $[M+Na]^+$), 605 (100%, $[2M+Na]^+$); HRMS (ESI) found *m/z* 313.9647 $[M+Na]^+$, $C_9H_{10}O_2NINa^+$ requires *m/z* 313.9648.

5-Iodobenzofuran (1s)

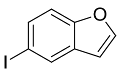

The procedure of Buchwald *et al.*[3] was used with the use of 5-bromobenzofuran (125 µL, 1.00 mmol) to give the titled *aryl iodide* **1s** as a colourless liquid (232 mg, 95%); ^{1}H NMR (400 MHz, $CDCl_3$) δ 7.95 (1H, d, *J* = 1.5, Ar-*H*), 7.60 (1H, d, *J* = 2.0, Ar-*H*), 7.57 (1H, dd, *J* = 8.5, 2.0, Ar-*H*), 7.30 (1H, d, *J* = 8.5, Ar-*H*), 6.72 (1H, dd, *J* = 2.0, 1.0, Ar-*H*); ^{13}C (100 MHz, $CDCl_3$) δ 154.3, 145.8, 132.8, 130.1, 130.0, 113.4, 105.8, 86.2; HRMS (FI) found *m/z* 243.9387, $C_8H_5OI^+$ (M^+) requires *m/z* 243.9385.

8-Chloro-3-iodoquinoline (1x)

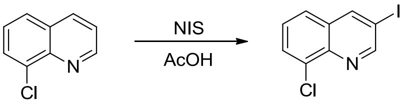

Procedure adapted from a Glaxo Group Limited patent.[2] *N*-Iodosuccinimide (7.6 g, 34 mmol) was added portionwise to a stirred solution of 8-chloroquinoline (5.0 g, 31 mmol) in acetic acid (35 mL) at 70 °C. The reaction mixture was stirred under nitrogen at the same temperature for 18 hrs and then allowed to cool to room temperature before being concentrated *in vacuo*. The resultant brown solid was dissolved in DCM (100 mL) and washed sequentially with $Na_2S_2O_{3(aq)}$ (100 mL) and $NaHCO_{3(aq)}$ (100 mL). The organic layer was dried ($MgSO_4$), filtered and concentrated *in vacuo*. The crude product was recrystallised twice from hot EtOAc to give the titled quinoline **1x** as an off-white crystalline solid (3.48 g, 39%); mp 98–99 °C (EtOAc); ^{1}H NMR (400 MHz, $CDCl_3$) δ 9.15 (1H, d, *J* = 2.0, Ar-*H*), 8.57 (1H, d, *J* = 2.0, Ar-*H*), 7.85 (1H, dd, *J* = 7.5, 1.5, Ar-*H*), 7.65 (1H, dd, *J* = 8.5, 1.0, Ar-*H*), 7.49 (1H, t, *J* = 8.0, Ar-*H*); ^{13}C (100 MHz, $CDCl_3$) δ 156.2, 144.1, 142.7, 133.8, 131.0, 130.2, 127.5, 125.9, 91.0; IR υ_{max} (neat)/cm^{-1} 3029, 1572, 1474, 1354, 1307, 1080; LRMS (ESI) *m/z* 290 (100%, [M(^{35}Cl)+H]$^+$), 292 (100%, [M(^{37}Cl)+H]$^+$); HRMS (ESI) found *m/z* 311.9055 [M+Na]$^+$, $C_9H_5N^{35}ClINa^+$ requires *m/z* 311.9047.

1-Iodocyclohept-1-ene (1y)

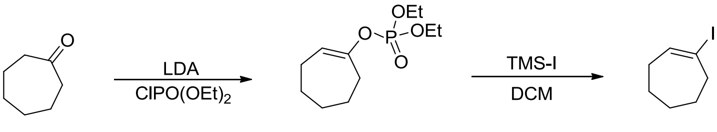

A solution of cycloheptanone (1.77 mL, 15.0 mmol) in dry THF (20 mL) was cooled to -78 °C. A solution of LDA in THF (1.8 M, 9.17 mL, 16.5 mmol) was added dropwise over 10 mins and the resulting solution stirred for 30 mins. A solution of diethylphosphorochloridate (8.06 g, 30.0 mmol) in THF (20 mL) was added dropwise at the same temperature and the resulting solution stirred for 30 mins, then for a further 1 hr at room temperature. The reaction mixture was cooled to 0 °C and quenched dropwise with sat. $NH_4Cl_{(aq)}$ (50 mL). The solution was extracted with EtOAc (3 × 60 mL)

and the organic layers combined, dried ($MgSO_4$), filtered and the solvent removed *in vacuo*. The crude oil was purified by flash column chromatography (10–50% EtOAc in petrol) affording the *alkenyl phosphate* as a pale yellow oil (2.61 g, 70%); ^{1}H NMR (400 MHz, $CDCl_3$) δ 5.65–5.61 (1H, m, C*H*), 4.19–4.11 (4H, m, 2 × OCH_2Me), 2.43–2.40 (2H, m, CH_2), 2.10–2.05 (2H, m, CH_2), 1.73–1.56 (6H, m, 3 × CH_2), 1.37–1.29 (6H, m, 2 × *Me*); ^{13}C NMR (100 MHz, $CDCl_3$) δ 152.2, 115.4, 64.0, 33.3, 30.6, 26.9, 25.0, 24.8, 16.1; IR υ_{max} (neat)/cm^{-1} 2926, 1678, 1445, 1369, 1268, 1158, 1104, 1019; LRMS (ESI) *m/z* 249 (5%, $[M+H]^+$), 520 (100%, $[2M+Na]^+$), 767 (90%, $[3M+Na]^+$). HRMS (ESI) found *m/z* 249.1247 $[M+H]^+$, $C_{11}H_{22}O_4P^+$ requires *m/z* 249.1250.

The titled *alkenyl iodide* **1y** (1.63 g, 70%) was synthesised as a colourless liquid, from the alkenyl phosphate (2.61 g, 10.5 mmol) as described by Wiemer *et al.*;[5] ^{1}H NMR (400 MHz, $CDCl_3$) δ 6.52 (1H, t, *J* = 6.5, C*H*), 2.78–2.75 (2H, m, CH_2), 2.06 (2H, app. q, *J* = 6.0, CH_2), 1.76–1.70 (2H, m, CH_2), 1.58–1.52 (4H, m, 2 × CH_2); ^{13}C NMR (100 MHz, $CDCl_3$) δ 142.8, 100.7, 45.0, 31.4, 31.0, 26.6, 26.3; HRMS (FI) found *m/z* 221.9905 (100%, $[M^+]$), $C_7H_{11}I^+$ requires *m/z* 221.9906.

General procedure for the formation of *β-tert*-butylacetate sulfones exemplified by the preparation of *tert*-butyl 2-((3,5-dimethylphenyl)sulfonyl)acetate (3g)

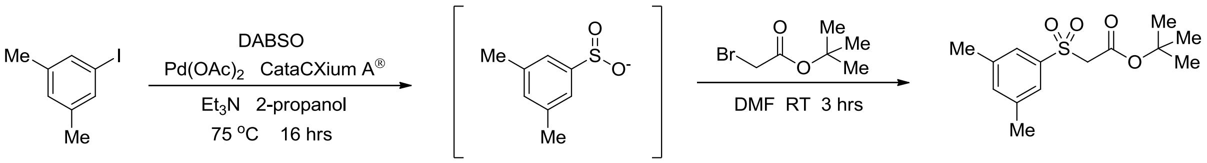

A glass reaction tube was charged with DABSO (43 mg, 0.18 mmol), palladium(II) acetate (4 mg, 15 µmol) and CataCXium A® (8 mg, 23 µmol), sealed with a rubber septum and evacuated and filled with nitrogen four times. 1-Iodo-3,5-dimethylbenzene (44 µL, 0.30 mmol), anhydrous triethylamine (125 µL, 0.9 mmol) and anhydrous 2-propanol (1.5 mL) were added sequentially through the septum and the reaction mixture stirred under positive pressure of nitrogen in a preheated oil bath at 75 °C for 16 hrs. After cooling to room temperature, a solution of *tert*-butyl bromoacetate (89 µL, 0.6 mmol) in anhydrous DMF (0.5 mL) was added dropwise and the resultant solution stirred at the same temperature for 3 hrs or until consumption of the sulfinate was observed by HPLC. Upon completion, the reaction mixture was poured onto water (20 mL) and extracted with EtOAc (3 × 20 mL). The combined organic fractions were dried ($MgSO_4$), filtered and concentrated *in vacuo*. Flash column chromatography (5–20% Et_2O in petrol) afforded the titled *sulfone* **3g** as a pale yellow microcrystalline solid (77 mg, 90%); mp 56–57 °C (DCM); ^{1}H NMR (400 MHz, $CDCl_3$) δ 7.54 (2H, s, Ar-*H*), 7.29 (1H, s, Ar-*H*), 4.01 (2H, s, CH_2), 2.40 (6H, s, 2 × Ar-*Me*), 1.37 (9H, s, C*Me*$_3$); ^{13}C (100 MHz, $CDCl_3$) δ 161.3, 139.2, 138.6, 135.7, 125.9, 83.4, 62.2, 27.6, 21.2; IR υ_{max} (neat)/cm^{-1} 2978, 1727 (C=O), 1610, 1457, 1371, 1323 (SO_2), 1287, 1133 (SO_2), 1102; LRMS (ESI) *m/z* 307 (60%,

[M+Na]$^+$), 591 (100%, [2M+Na]$^+$); HRMS (ESI) found *m/z* 307.0978 [M+Na]$^+$, $C_{14}H_{20}O_4SNa^+$ requires *m/z* 307.0975.

Procedure as follows for large scale reaction:

A 100 mL round-bottom-flask was charged with DABSO (1.73 g, 7.2 mmol), palladium(II) acetate (27 mg, 0.12 mmol) and CataCXium A® (65 mg, 0.18 mmol), sealed with a rubber septum and evacuated and filled with nitrogen four times. 1-Iodo-3,5-dimethylbenzene (1.7 mL, 12 mmol), anhydrous triethylamine (5.0 mL, 36 mmol) and anhydrous 2-propanol (30 mL) were added sequentially through the septum and the reaction mixture stirred under positive pressure of nitrogen on a metal heating block at 75 ^{o}C for 16 hrs. After cooling to room temperature, a solution of *tert*-butyl bromoacetate (3.5 mL, 24 mmol) in anhydrous DMF (10 mL) was added dropwise over 20 mins and the resultant solution stirred at the same temperature for 2 hrs until consumption of the sulfinate was observed by HPLC. Upon completion, the reaction mixture was poured onto water (150 mL) and extracted with EtOAc (3 × 150 mL). The combined organic fractions were washed with water (150 mL) and brine (150 mL), dried ($MgSO_4$), filtered and concentrated *in vacuo*. Automated flash column chromatography (0–20% EtOAc in petrol) afforded the titled *sulfone* **3g** as a pale yellow oil which crystallised to an off-white microcrystalline solid with standing (3.28 g, 96%). Data as above.

***tert*-Butyl 2-((4-methoxyphenyl)sulfonyl)acetate (3a)**

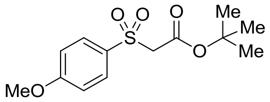

The general procedure was followed with the use of 4-iodoanisole (70 mg, 0.30 mmol). Flash column chromatography (20–40% Et_2O in petrol) afforded the titled *sulfone* **3a** as a pale yellow oil (75 mg, 87%); ^{1}H NMR (400 MHz, $CDCl_3$) δ 7.86 (2H, d, *J* = 9.0, Ar-*H*), 7.02 (2H, d, *J* = 9.0, Ar-*H*), 4.01 (2H, s, C*H$_2$*), 3.89 (3H, s, O*Me*), 1.39 (9H, s, C*Me$_3$*); ^{13}C (100 MHz, $CDCl_3$) δ 164.0, 161.5, 130.8, 130.4, 114.2, 83.4, 62.3, 55.7, 27.7; IR υ_{max} (neat)/cm^{-1} 2980, 1729 (C=O), 1595, 1499, 1370, 1326 (SO_2), 1299, 1259, 1140 (SO_2), 1085, 1022; LRMS (ESI) *m/z* 304 (40%, [M+NH_4]$^+$), 309 (35%, [M+Na]$^+$), 595 (100%, [2M+Na]$^+$); HRMS (ESI) found *m/z* 309.0773 [M+Na]$^+$, $C_{13}H_{18}O_5SNa^+$ requires *m/z* 309.0767.

***tert*-Butyl 2-((3-methoxyphenyl)sulfonyl)acetate (3b)**

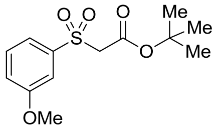

The general procedure was followed with the use of 3-iodoanisole (36 µL, 0.30 mmol). Flash column chromatography (15–40% Et_2O in petrol) afforded the titled *sulfone* **3b** as a pale yellow oil (74 mg, 86%); ^{1}H NMR (400 MHz, $CDCl_3$) δ 7.54–7.45 (2H, m, Ar-*H*), 7.43 (1H, t, *J* = 2.0, Ar-*H*), 7.19 (1H, ddd, *J* = 8.0, 2.5, 1.0, Ar-*H*), 4.03 (2H, s, CH_2), 3.87 (3H, s, O*Me*), 1.37 (9H, s, CMe_3); ^{13}C (100 MHz, $CDCl_3$) δ 161.1, 159.8, 139.9, 130.2, 120.54, 120.51, 112.9, 83.5, 62.0, 55.7, 27.6; IR υ_{max} (neat)/cm^{-1} 2980, 1730 (C=O), 1598, 1482, 1316 (SO_2), 1288, 1247, 1140 (SO_2), 1091, 1035; LRMS (ESI) *m/z* 304 (35%, $[M+NH_4]^+$), 309 (30%, $[M+Na]^+$), 595 (100%, $[2M+Na]^+$); HRMS (ESI) found *m/z* 309.0765 $[M+Na]^+$, $C_{13}H_{18}O_5SNa^+$ requires *m/z* 309.0767.

***tert*-Butyl 2-((2-methoxyphenyl)sulfonyl)acetate (3c)**

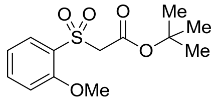

The general procedure was followed with the use of 2-iodoanisole (39 µL, 0.30 mmol). Flash column chromatography (25–50% Et_2O in petrol) afforded the titled *sulfone* **3c** as an off-white microcrystalline solid (67 mg, 78%); mp 89–90 °C (DCM); ^{1}H NMR (400 MHz, $CDCl_3$) δ 7.95 (1H, dd, *J* = 8.0, 1.5, Ar-*H*), 7.62 (1H, ddd, *J* = 7.5, 1.5, 1.0, Ar-*H*), 7.12 (1H, t, *J* = 7.5, Ar-*H*), 7.07 (1H, d, *J* = 8.5, Ar-*H*), 4.30 (3H, s, O*Me*), 4.01 (2H, s, CH_2), 1.24 (9H, s, CMe_3); ^{13}C (100 MHz, $CDCl_3$) δ 161.5, 157.3, 135.8, 130.6, 126.8, 120.6, 112.2, 83.1, 60.3, 56.3, 27.5; IR υ_{max} (neat)/cm^{-1} 2944, 1721 (C=O), 1590, 1479, 1434, 1323 (SO_2), 1304, 1276, 1220, 1153 (SO_2), 1099, 1016; LRMS (ESI) *m/z* 304 (40%, $[M+NH_4]^+$), 309 (35%, $[M+Na]^+$), 595 (100%, $[2M+Na]^+$); HRMS (ESI) found *m/z* 309.0775 $[M+Na]^+$, $C_{13}H_{18}O_5SNa^+$ requires *m/z* 309.0767.

***tert*-Butyl 2-((4-(methylthio)phenyl)sulfonyl)acetate (3d)**

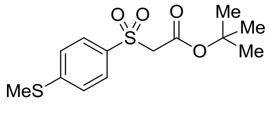

The general procedure was followed with the use of 4-iodothioanisole (75 mg, 0.30 mmol). Flash column chromatography (15–35% Et_2O in petrol) afforded the titled *sulfone* **3d** as a yellow oil (79 mg, 87%); ^{1}H NMR (400 MHz, $CDCl_3$) δ 7.80 (2H, d, *J* = 8.5, Ar-*H*), 7.34 (2H, d, *J* = 8.5, Ar-*H*), 4.01 (2H, s, CH_2), 2.52 (3H, s, S*Me*), 1.38 (9H, s, CMe_3); ^{13}C (100 MHz, $CDCl_3$) δ 161.3, 147.8, 134.3, 128.7, 125.1, 83.5, 62.1, 27.6, 14.6; IR υ_{max} (neat)/cm^{-1} 2980, 1728 (C=O), 1579, 1395, 1322 (SO_2), 1292, 1146 (SO_2), 1095, 1078; LRMS (ESI) *m/z* 320 (50%, $[M+NH_4]^+$), 325 (50%, $[M+Na]^+$),

627 (100%, $[2M+Na]^+$); HRMS (ESI) found *m/z* 325.0543 $[M+Na]^+$, $C_{13}H_{18}O_4S_2Na^+$ requires *m/z* 325.0539.

***tert*-Butyl 2-((4-hydroxyphenyl)sulfonyl)acetate (3e)**

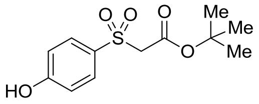

The general procedure was followed with the use of 4-iodophenol (66 mg, 0.30 mmol). Flash column chromatography (40–70% Et_2O in petrol) afforded the titled *sulfone* **3e** as a pale yellow oil (61 mg, 75%); ^{1}H NMR (400 MHz, $CDCl_3$) δ 7.77 (2H, d, *J* = 9.0, Ar-*H*), 6.94 (2H, d, *J* = 9.0, Ar-*H*), 4.05 (2H, s, C*H*$_2$), 1.40 (9H, s, C*Me*$_3$); ^{13}C (100 MHz, $CDCl_3$) δ 162.0, 161.6, 130.9, 129.3, 116.0, 84.2, 62.4, 27.7; IR υ_{max} (neat)/cm^{-1} 3383 br. (OH), 2981, 1728 (C=O), 1586, 1502, 1370, 1312 (SO_2), 1288, 1136 (SO_2), 1083; LRMS (ESI) *m/z* 290 (45%, $[M+NH_4]^+$), 295 (45%, $[M+Na]^+$), 567 (100%, $[2M+Na]^+$); HRMS (ESI) found *m/z* 295.0613 $[M+Na]^+$, $C_{12}H_{16}O_5SNa^+$ requires *m/z* 295.0611.

***tert*-Butyl 2-((4-aminophenyl)sulfonyl)acetate (3f)**

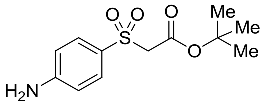

The general procedure was followed with the use of 4-iodoaniline (66 mg, 0.30 mmol). Flash column chromatography (60–75% Et_2O in petrol) afforded the titled *sulfone* **3f** as an off-white microcrystalline solid (52 mg, 64%); mp 105–107 °C (DCM); ^{1}H NMR (400 MHz, $CDCl_3$) δ 7.68 (2H, d, *J* = 9.0, Ar-*H*), 6.71 (2H, d, *J* = 9.0, Ar-*H*), 3.98 (2H, s, C*H*$_2$), 1.39 (9H, s, C*Me*$_3$); ^{13}C (100 MHz, $CDCl_3$) δ 161.8, 151.7, 130.7, 126.7, 113.9, 83.2, 62.5, 27.7; IR υ_{max} (neat)/cm^{-1} 3486, 3379 (NH_2), 2945, 1732 (C=O), 1627, 1596, 1508, 1372, 1317 (SO_2), 1287, 1129 (SO_2), 1084; LRMS (ESI) *m/z* 289 (45%, $[M+NH_4]^+$), 294 (40%, $[M+Na]^+$), 565 (100%, $[2M+Na]^+$); HRMS (ESI) found *m/z* 294.0777 $[M+Na]^+$, $C_{12}H_{17}O_4SNNa^+$ requires *m/z* 294.0770.

***tert*-Butyl 2-(*o*-tolylsulfonyl)acetate (3h)**

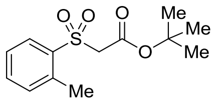

The general procedure was followed with the use of 2-iodotoluene (38 µL, 0.30 mmol). Flash column chromatography (10–25% Et_2O in petrol) afforded the titled *sulfone* **3h** as a pale yellow microcrystalline solid (62 mg, 76%); mp 66–67 °C (DCM); ^{1}H NMR (400 MHz, $CDCl_3$) δ 8.02 (1H, dd, *J* = 8.0, 1.0, Ar-*H*), 7.55 (1H, td, *J* = 7.5, 1.5, Ar-*H*), 7.41–7.34 (2H, m, Ar-*H*), 4.09 (2H, s, C*H*$_2$), 2.72 (3H, s, Ar-*Me*), 1.29 (9H, s, C*Me*$_3$); ^{13}C (100 MHz, $CDCl_3$) δ 161.1, 138.1, 137.0, 134.1, 132.6,

130.7, 126.4, 83.5, 61.5, 27.5, 20.3; IR υ_{max} (neat)/cm^{-1} 2945, 1720 (C=O), 1470, 1365, 1317 (SO_2), 1224, 1156 (SO_2), 1097, 1060; LRMS (ESI) *m/z* 288 (85%, $[M+NH_4]^+$), 293 (50%, $[M+Na]^+$), 563 (100%, $[2M+Na]^+$); HRMS (ESI) found *m/z* 293.0822 $[M+Na]^+$, $C_{13}H_{18}O_4SNa^+$ requires *m/z* 293.0818.

***tert*-Butyl 2-((4-(trimethylsilyl)phenyl)sulfonyl)acetate (3i)**

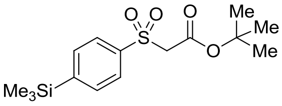

The general procedure was followed with the use of (4-iodophenyl)trimethylsilane (83 mg, 0.30 mmol). Flash column chromatography (5–25% Et_2O in petrol) afforded the titled *sulfone* **3i** as a colourless oil (71 mg, 72%); ^{1}H NMR (400 MHz, $CDCl_3$) δ 7.89 (2H, d, *J* = 8.0, Ar-*H*), 7.71 (2H, d, *J* = 8.0, Ar-*H*), 4.03 (2H, s, C*H$_2$*), 1.35 (9H, s, C*Me$_3$*), 0.30 (9H, s, Si*Me$_3$*); ^{13}C (100 MHz, $CDCl_3$) δ 161.2, 148.9, 139.0, 133.8, 127.2, 83.5, 62.1, 27.6, -1.5; IR υ_{max} (neat)/cm^{-1} 2956, 1732 (C=O), 1370, 1327 (SO_2), 1291, 1251, 1145 (SO_2), 1103, 1080; LRMS (ESI) *m/z* 346 (100%, $[M+NH_4]^+$), 351 (75%, $[M+Na]^+$), 679 (85%, $[2M+Na]^+$); HRMS (ESI) found *m/z* 351.1059 $[M+Na]^+$, $C_{15}H_{24}O_4SSiNa^+$ requires *m/z* 351.1057.

***tert*-Butyl 2-(naphthalen-1-ylsulfonyl)acetate (3j)**

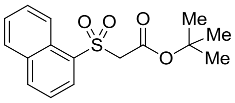

The general procedure was followed with the use of 1-iodonaphthalene (44 µL, 0.30 mmol). Flash column chromatography (10–25% Et_2O in petrol) afforded the titled *sulfone* **3j** as an off-white microcrystalline solid (69 mg, 75%); mp 85–87 °C (DCM); ^{1}H NMR (400 MHz, $CDCl_3$) δ 8.70 (1H, dd, *J* = 8.5, 0.5, Ar-*H*), 8.34 (1H, dd, *J* = 7.5, 1.0, Ar-*H*), 8.17 (1H, d, *J* = 8.0, Ar-*H*), 8.00 (1H, d, *J* = 8.0, Ar-*H*), 7.77–7.71 (1H, m, Ar-*H*), 7.67–7.60 (2H, m, Ar-*H*), 4.25 (2H, s, C*H$_2$*), 1.25 (9H, s, C*Me$_3$*); ^{13}C (100 MHz, $CDCl_3$) δ 161.0, 135.6, 134.1, 133.8, 131.3, 129.4, 128.9, 128.7, 127.0, 124.2, 123.7, 83.4, 61.8, 27.5; IR υ_{max} (neat)/cm^{-1} 2944, 1724 (C=O), 1506, 1370, 1319 (SO_2), 1290, 1150 (SO_2), 1119; LRMS (ESI) *m/z* 324 (80%, $[M+NH_4]^+$), 329 (50%, $[M+Na]^+$), 635 (100%, $[2M+Na]^+$); HRMS (ESI) found *m/z* 329.0827 $[M+Na]^+$, $C_{16}H_{18}O_4SNa^+$ requires *m/z* 329.0818.

Methyl 4-((2-(*tert*-butoxy)-2-oxoethyl)sulfonyl)benzoate (3k)

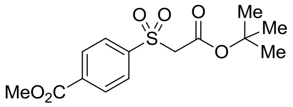

The general procedure was followed with the use of methyl 4-iodobenzoate (79 mg, 0.30 mmol). Flash column chromatography (20–40% Et_2O in petrol) afforded the titled *sulfone* **3k** as a pale yellow microcrystalline solid (70 mg, 74%); mp 89–90 °C (DCM); ^{1}H NMR (400 MHz, $CDCl_3$) δ 8.23 (2H, d, *J* = 8.5, Ar-*H*), 8.03 (2H, d, *J* = 8.5, Ar-*H*), 4.07 (2H, s, C*H₂*), 3.97 (3H, s, CO_2*Me*), 1.37 (9H, s, C*Me₃*); ^{13}C (100 MHz, $CDCl_3$) δ 165.4, 161.0, 142.6, 135.1, 130.2, 128.6, 83.9, 61.8, 52.7, 27.7; IR υ_{max} (neat)/cm^{-1} 1723 (C=O), 1401, 1329 (SO_2), 1303, 1272, 1159 (SO_2), 1102, 1082, 1017; LRMS (ESI) *m/z* 332 (90%, $[M+NH_4]^+$), 337 (80%, $[M+Na]^+$), 651 (100%, $[2M+Na]^+$); HRMS (ESI) found *m/z* 337.0724 $[M+Na]^+$, $C_{14}H_{18}O_6SNa^+$ requires *m/z* 337.0716.

***tert*-Butyl 2-((4-acetylphenyl)sulfonyl)acetate (3l)**

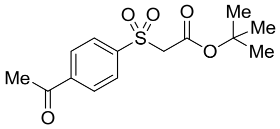

The general procedure was followed with the use of 4'-iodoacetophenone (74 mg, 0.30 mmol). Flash column chromatography (25–60% Et_2O in petrol) afforded the titled *sulfone* **3l** as an off-white microcrystalline solid (66 mg, 74%); mp 81–82 °C (DCM); ^{1}H NMR (400 MHz, $CDCl_3$) δ 8.14 (2H, d, *J* = 8.5, Ar-*H*), 8.05 (2H, d, *J* = 8.5, Ar-*H*), 4.07 (2H, s, C*H₂*), 2.68 (3H, s, CO*Me*), 1.38 (9H, s, C*Me₃*); ^{13}C (100 MHz, $CDCl_3$) δ 196.6, 161.0, 142.5, 141.0, 129.0, 128.8, 84.0, 61.8, 27.7, 26.9; IR υ_{max} (neat)/cm^{-1} 2927, 1725 (C=O ester), 1685 (C=O ketone), 1402, 1371, 1331 (SO_2), 1305, 1262, 1143 (SO_2), 1122, 1090, 1019; LRMS (ESI) *m/z* 316 (85%, $[M+NH_4]^+$), 321 (80%, $[M+Na]^+$), 619 (100%, $[2M+Na]^+$); HRMS (ESI) found *m/z* 321.0771 $[M+Na]^+$, $C_{14}H_{18}O_5SNa^+$ requires *m/z* 321.0767.

***tert*-Butyl 2-((4-(methoxy(methyl)carbamoyl)phenyl)sulfonyl)acetate (3m)**

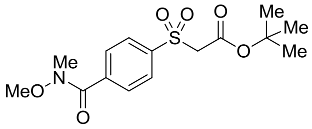

The general procedure was followed with the use of 4-iodo-*N*-methoxy-*N*-methylbenzamide (87 mg, 0.30 mmol). Flash column chromatography (40–60% EtOAc in petrol) afforded the titled *sulfone* **3m** as a pale yellow oil (74 mg, 72%); ^{1}H NMR (400 MHz, $CDCl_3$) δ 7.98 (2H, d, *J* = 8.5, Ar-*H*), 7.84 (2H, d, *J* = 8.5, Ar-*H*), 4.06 (2H, s, C*H₂*), 3.51 (3H, s, N*Me*), 3.37 (3H, s, O*Me*), 1.36 (9H, s, C*Me₃*); ^{13}C (100 MHz, $CDCl_3$) δ 167.8, 160.9, 140.4, 139.6, 128.8, 128.2, 83.8, 61.8, 61.3, 33.1, 27.6; IR υ_{max} (neat)/cm^{-1} 2980, 2938, 1731 (C=O), 1643, 1370, 1327 (SO_2), 1290, 1143 (SO_2), 1084; LRMS (ESI)

m/z 344 (90%, $[M+H]^+$), 361 (30%, $[M+NH_4]^+$), 366 (100%, $[M+Na]^+$), 709 (100%, $[2M+Na]^+$); HRMS (ESI) found *m/z* 366.0988 $[M+Na]^+$, $C_{15}H_{21}O_6SNNa^+$ requires *m/z* 366.0982.

***tert*-Butyl 2-((4-cyanophenyl)sulfonyl)acetate (3n)**

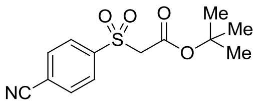

The general procedure was followed with the use of 4-iodobenzonitrile (69 mg, 0.30 mmol). Flash column chromatography (20–35% Et_2O in petrol) afforded the titled *sulfone* **3n** as an off-white microcrystalline solid (51 mg, 60%); mp 95–96 °C (DCM); ^1H NMR (400 MHz, $CDCl_3$) δ 8.09 (2H, d, *J* = 8.5, Ar-*H*), 7.89 (2H, d, *J* = 8.5, Ar-*H*), 4.09 (2H, s, C*H₂*), 1.40 (9H, s, C*Me₃*); ^{13}C (100 MHz, $CDCl_3$) δ 160.9, 142.8, 132.8, 129.4, 117.9, 117.0, 84.3, 61.6, 27.7; IR υ_{max} (neat)/cm^{-1} 2231 (CN), 1731 (C=O), 1371, 1327 (SO_2), 1287, 1252, 1141 (SO_2), 1082; LRMS (ESI) *m/z* 299 (45%, $[M+NH_4]^+$), 304 (100%, $[M+Na]^+$); HRMS (ESI) found *m/z* 304.0619 $[M+Na]^+$, $C_{13}H_{15}O_4NSNa^+$ requires *m/z* 304.0614.

***tert*-Butyl 2-((3-(trifluoromethyl)phenyl)sulfonyl)acetate (3o)**

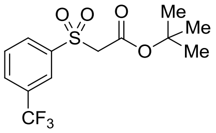

The general procedure was followed with the use of 3-iodobenzotrifluoride (44 µL, 0.30 mmol). Flash column chromatography (5–20% Et_2O in petrol) afforded the titled *sulfone* **3o** as a pale yellow microcrystalline solid (78 mg, 80%); mp 60–61 °C (DCM); ^1H NMR (400 MHz, $CDCl_3$) δ 8.22 (1H, s, Ar-*H*), 8.16 (1H, d, *J* = 8.0, Ar-*H*), 7.95 (1H, d, *J* = 8.0, Ar-*H*), 7.76 (1H, t, *J* = 8.0, Ar-*H*), 4.09 (2H, s, C*H₂*), 1.37 (9H, s, C*Me₃*); ^{13}C (125 MHz, $CDCl_3$) δ 160.9, 140.0, 131.9, 131.8 (q, *J* = 34), 130.8 (q, *J* = 3.5), 130.0, 125.8 (q, *J* = 3.5), 123.0 (q, *J* = 273), 84.1, 61.9, 27.6; ^{19}F {^1H} (376 MHz, $CDCl_3$) δ -62.8 (s); IR υ_{max} (neat)/cm^{-1} 1734 (C=O), 1610, 1373, 1327 (SO_2), 1303, 1290, 1143, 1120 (SO_2), 1102, 1072; LRMS (ESI) *m/z* 342 (100%, $[M+NH_4]^+$), 347 (95%, $[M+Na]^+$), 671 (80%, $[2M+Na]^+$); HRMS (ESI) found *m/z* 347.0541 $[M+Na]^+$, $C_{13}H_{15}O_4SF_3Na^+$ requires *m/z* 347.0535.

***tert*-Butyl 2-((4-bromophenyl)sulfonyl)acetate (3p)**

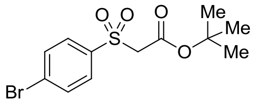

The general procedure was followed with the use of 1-bromo-4-iodobenzene (85 mg, 0.30 mmol). Flash column chromatography (10–30% Et_2O in petrol) afforded the titled *sulfone* **3p** as a pale yellow microcrystalline solid (63 mg, 63%); mp 73–74 °C (DCM); ^1H NMR (400 MHz, $CDCl_3$) δ 7.81 (2H,

d, J = 8.5, Ar-H), 7.73 (2H, d, J = 8.5, Ar-H), 4.04 (2H, s, CH_2), 1.39 (9H, s, CMe_3); ^{13}C (100 MHz, $CDCl_3$) δ 161.1, 137.8, 132.4, 130.1, 129.5, 83.9, 61.9, 27.7; IR υ_{max} (neat)/cm^{-1} 2996, 2937, 1717 (C=O), 1573, 1470, 1392, 1330 (SO_2), 1297, 1249, 1157 (SO_2), 1111, 1082, 1067, 1011; LRMS (ESI) *m/z* 352 (95%, [M(^{79}Br)+NH_4]$^+$), 354 (95%, [M(^{81}Br)+NH_4]$^+$), 357 (100%, [M(^{79}Br)+Na]$^+$), 359 (100%, [M(^{81}Br)+Na]$^+$), 693 (100%, [2M(^{79}Br,^{81}Br)+Na]$^+$); HRMS (ESI) found *m/z* 356.9771 [M(^{79}Br)+Na]$^+$, $C_{12}H_{15}O_4S^{79}BrNa^+$ requires *m/z* 356.9767.

***tert*-Butyl 2-((4-chlorophenyl)sulfonyl)acetate (3q)**

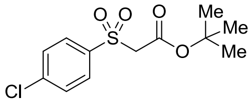

The general procedure was followed with the use of 1-chloro-4-iodobenzene (72 mg, 0.30 mmol). Flash column chromatography (10–30% Et_2O in petrol) afforded the titled *sulfone* **3q** as a pale yellow microcrystalline solid (71 mg, 81%); mp 49–51 °C (DCM); ^{1}H NMR (400 MHz, $CDCl_3$) δ 7.89 (2H, d, J = 8.5, Ar-H), 7.56 (2H, d, J = 8.5, Ar-H), 4.04 (2H, s, CH_2), 1.40 (9H, s, CMe_3); ^{13}C (100 MHz, $CDCl_3$) δ 161.2, 141.0, 137.3, 130.1, 129.4, 83.9, 62.0, 27.7; IR υ_{max} (neat)/cm^{-1} 2981, 1730 (C=O), 1583, 1476, 1395, 1370, 1328 (SO_2), 1292, 1145 (SO_2), 1084, 1014; LRMS (ESI) *m/z* 308 (100%, [M(^{35}Cl)+NH_4]$^+$), 310 (40%, [M(^{37}Cl)+NH_4]$^+$), 313 (95%, [M(^{35}Cl)+Na]$^+$), 315 (35%, [M(^{37}Cl)+Na]$^+$), 603 (90%, [2M(^{35}Cl,^{35}Cl)+Na]$^+$); HRMS (ESI) found *m/z* 313.0278 [M(^{35}Cl)+Na]$^+$, $C_{12}H_{15}O_4S^{35}ClNa^+$ requires *m/z* 313.0272.

***tert*-Butyl 2-((3-fluorophenyl)sulfonyl)acetate (3r)**

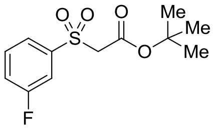

The general procedure was followed with the use of 1-fluoro-3-iodobenzene (35 µL, 0.30 mmol). Flash column chromatography (10–30% Et_2O in petrol) afforded the titled *sulfone* **3r** as a pale yellow oil (58 mg, 70%); ^{1}H NMR (400 MHz, $CDCl_3$) δ 7.76 (1H, ddd, J = 8.0, 1.5, 1.0, Ar-H), 7.66 (1H, dt, J = 8.0, 2.0, Ar-H), 7.59 (1H, td, J = 8.0, 5.0, Ar-H), 7.39 (1H, tdd, J = 8.0, 2.5, 1.0, Ar-H), 4.05 (2H, s, CH_2), 1.38 (9H, s, CMe_3); ^{13}C (100 MHz, $CDCl_3$) δ 162.2 (d, J = 253), 160.9, 140.8 (d, J = 7), 131.0 (d, J = 8), 124.3 (d, J = 3), 121.4 (d, J = 21), 116.0 (d, J = 25), 83.9, 61.9, 27.6; ^{19}F {^{1}H} (376 MHz, $CDCl_3$) δ -109.1 (s); IR υ_{max} (neat)/cm^{-1} 2982, 1731 (C=O), 1594, 1478, 1371, 1331 (SO_2), 1304, 1225, 1138 (SO_2), 1083; LRMS (ESI) *m/z* 292 (55%, [M+NH_4]$^+$), 297 (80%, [M+Na]$^+$), 571 (100%, [2M+Na]$^+$); HRMS (ESI) found *m/z* 297.0565 [M+Na]$^+$, $C_{12}H_{15}O_4SFNa^+$ requires *m/z* 297.0567.

***tert*-Butyl 2-(benzofuran-5-ylsulfonyl)acetate (3s)**

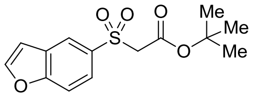

The general procedure was followed with the use of 5-iodobenzofuran (73 mg, 0.30 mmol). Flash column chromatography (20–40% Et_2O in petrol) afforded the titled *sulfone* **3s** as a pale yellow oil (71 mg, 80%); ^{1}H NMR (400 MHz, $CDCl_3$) δ 8.25 (1H, d, *J* = 2.0, Ar-*H*), 7.89 (1H, dd, *J* = 8.5, 2.0, Ar-*H*), 7.79 (1H, d, *J* = 2.0, Ar-*H*), 7.67 (1H, d, *J* = 8.5), 6.91 (1H, dd, *J* = 2.0, 1.0, Ar-*H*), 4.08 (2H, s, C*H₂*), 1.35 (9H, s, C*Me₃*); ^{13}C (100 MHz, $CDCl_3$) δ 161.4, 157.4, 147.5, 133.6, 127.8, 124.6, 123.1, 112.1, 107.1, 83.5, 62.5, 27.6; IR υ_{max} (neat)/cm^{-1} 2981, 1728 (C=O), 1453, 1370, 1318 (SO_2), 1292, 1261, 1125 (SO_2), 1110, 1061, 1027; LRMS (ESI) *m/z* 314 (40%, $[M+NH_4]^+$), 319 (35%, $[M+Na]^+$), 615 (100%, $[2M+Na]^+$); HRMS (ESI) found *m/z* 319.0619 $[M+Na]^+$, $C_{14}H_{16}O_5SNa^+$ requires *m/z* 319.0611.

***tert*-Butyl 2-((1H-indol-5-yl)sulfonyl)acetate (3t)**

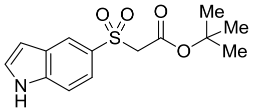

The general procedure was followed with the use of 5-iodoindole (73 mg, 0.30 mmol). Flash column chromatography (30–60% Et_2O in petrol) afforded the titled *sulfone* **3t** as an off-white microcrystalline solid (61 mg, 69%); mp 104–105 ^{o}C (DCM); ^{1}H NMR (400 MHz, $CDCl_3$) δ 9.04 (1H, br. s, N*H*), 8.27 (1H, d, *J* = 1.5, Ar-*H*), 7.70 (1H, dd, *J* = 8.5, 2.0, Ar-*H*), 7.52 (1H, d, *J* = 8.5, Ar-*H*), 7.38 (1H, d, *J* = 3.0), 6.68–6.66 (1H, m, Ar-*H*), 4.09 (2H, s, CH_2), 1.33 (9H, s, CMe_3); ^{13}C (100 MHz, $CDCl_3$) δ 161.7, 138.4, 129.5, 127.4, 127.1, 122.8, 121.1, 111.7, 104.0, 83.4, 62.8, 27.6; IR υ_{max} (neat)/cm^{-1} 3339 (NH), 2983, 1731 (C=O), 1311 (SO_2), 1293, 1140 (SO_2), 1119, 1059; LRMS (ESI) *m/z* 313 (55%, $[M+NH_4]^+$), 318 (30%, $[M+Na]^+$), 613 (100%, $[2M+Na]^+$); HRMS (ESI) found *m/z* 318.0774 $[M+Na]^+$, $C_{14}H_{17}O_4SNNa^+$ requires *m/z* 318.0770.

***tert*-Butyl 2-(benzo[b]thiophen-3-ylsulfonyl)acetate (3u)**

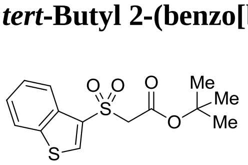

The general procedure was followed with the use of 3-iodobenzothiophene[3] (78 mg, 0.30 mmol). Flash column chromatography (15–30% Et_2O in petrol) afforded the titled *sulfone* **3u** as an off-white microcrystalline solid (50 mg, 53%); mp 116–117 ^{o}C (DCM); ^{1}H NMR (400 MHz, $CDCl_3$) δ 8.40 (1H, s, Ar-*H*), 8.24 (1H, d, *J* = 8.0, Ar-*H*), 7.94 (1H, d, *J* = 8.0, Ar-*H*), 7.59–7.48 (2H, m, Ar-*H*), 4.15 (2H, s, CH_2), 1.32 (9H, s, CMe_3); ^{13}C (100 MHz, $CDCl_3$) δ 161.0, 140.3, 138.0, 133.8, 132.7, 126.1,

126.0, 123.1, 122.8, 83.6, 61.8, 27.6; IR υ_{max} (neat)/cm^{-1} 3081, 1715 (C=O), 1457, 1420, 1367, 1332, 1322 (SO_2), 1310, 1231, 1158 (SO_2), 1104; LRMS (ESI) *m/z* 335 (100%, $[M+Na]^+$), 650 (95%, $[2M+Na]^+$); HRMS (ESI) found *m/z* 335.0390 $[M+Na]^+$, $C_{14}H_{16}O_4S_2Na^+$ requires *m/z* 335.0382.

***tert*-Butyl 2-(thiophen-3-ylsulfonyl)acetate (3v)**

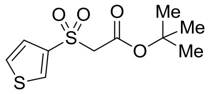

The general procedure was followed with the use of 3-iodothiophene (31 µL, 0.30 mmol). Flash column chromatography (20–40% Et_2O in petrol) afforded the titled *sulfone* **3v** as a pale yellow oil (50 mg, 64%); ^{1}H NMR (400 MHz, $CDCl_3$) δ 8.14 (1H, dd, *J* = 3.0, 1.5, Ar-*H*), 7.48 (1H, dd, *J* = 5.0, 3.0, Ar-*H*), 7.44 (1H, dd, *J* = 5.0, 1.5, Ar-*H*), 4.05 (2H, s, C*H₂*), 1.40 (9H, s, C*Me₃*); ^{13}C (100 MHz, $CDCl_3$) δ 161.3, 139.2, 133.6, 128.0, 126.4, 83.7, 62.2, 27.7; IR υ_{max} (neat)/cm^{-1} 2980, 1728 (C=O), 1370, 1322 (SO_2), 1293, 1208, 1139 (SO_2), 1095; LRMS (ESI) *m/z* 280 (50%, $[M+NH_4]^+$), 285 (45%, $[M+Na]^+$), 547 (100%, $[2M+Na]^+$); HRMS (ESI) found *m/z* 285.0228 $[M+Na]^+$, $C_{10}H_{14}O_4S_2Na^+$ requires *m/z* 285.0226.

***tert*-Butyl 2-((6-methoxypyridin-3-yl)sulfonyl)acetate (3w)**

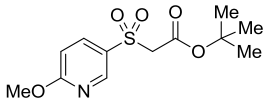

The general procedure was followed with the use of 5-iodo-2-methoxypyridine[6] (71 mg, 0.30 mmol). Flash column chromatography (20–40% Et_2O in petrol) afforded the titled *sulfone* **3w** as a yellow oil (57 mg, 66%); ^{1}H NMR (400 MHz, $CDCl_3$) δ 8.71 (1H, d, *J* = 2.5, Ar-*H*), 8.03, (1H, dd, *J* = 9.0, 2.5, Ar-*H*), 6.87 (1H, d, *J* = 9.0, Ar-*H*), 4.03 (5H, 2 overlapping s, OMe and C*H₂*), 1.42 (9H, s, C*Me₃*); ^{13}C (100 MHz, $CDCl_3$) δ 167.3, 161.4, 149.5, 138.6, 128.1, 111.3, 83.9, 62.4, 54.5, 27.7; IR υ_{max} (neat)/cm^{-1} 2981, 2946, 1730 (C=O), 1590, 1564, 1483, 1372, 1330 (SO_2), 1287, 1151 (SO_2), 1127, 1094, 1010; LRMS (ESI) *m/z* 288 (100%, $[M+H]^+$), 310 (70%, $[M+Na]^+$), 597 (90%, $[2M+Na]^+$); HRMS (ESI) found *m/z* 310.0728 $[M+Na]^+$, $C_{12}H_{17}O_5SNNa^+$ requires *m/z* 310.0720.

***tert*-Butyl 2-((8-chloroquinolin-3-yl)sulfonyl)acetate (3x)**

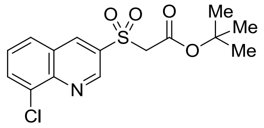

The general procedure was followed with the use of 8-chloro-3-iodoquinoline (87 mg, 0.30 mmol), palladium(II) acetate (7 mg, 30 µmol), CataCXium A® (16 mg, 45 µmol), DABSO (79 mg, 0.33 mmol), triethylamine (125 µL, 0.9 mmol) and 2-propanol (1.5 mL). Flash column chromatography

(0–60% acetone in petrol) afforded the titled *sulfone* **3x** as an off-white microcrystalline solid (70 mg, 68%); mp 124–125 ^{o}C (DCM); ^{1}H NMR (400 MHz, $CDCl_3$) δ 9.41 (1H, d, *J* = 2.0, Ar-*H*), 8.83 (1H, d, *J* = 2.0, Ar-*H*), 8.05 (1H, dd, *J* = 7.5, 1.5, Ar-*H*), 7.94 (1H, dd, *J* = 8.0, 1.0, Ar-*H*), 7.66 (1H, t, *J* = 8.0, Ar-*H*), 4.18 (2H, s, C*H*$_2$), 1.37 (9H, s, C*Me*$_3$); ^{13}C (100 MHz, $CDCl_3$) δ 161.0, 148.0, 146.0, 139.3, 134.2, 133.1, 132.9, 128.5, 128.3, 127.5, 84.3, 62.1, 27.7; IR υ_{max} (neat)/cm^{-1} 1728 (C=O), 1336 (SO_2), 1308, 1215, 1165, 1121 (SO_2), 1083; LRMS (ESI) *m/z* 342 (100%, [M(^{35}Cl)+H]$^+$), 344 (30%, [M(^{37}Cl)+H]$^+$), 364 (30%, [M(^{35}Cl)+Na]$^+$), 366 (10%, [M(^{37}Cl)+Na]$^+$), 705 (45%, [2M(^{35}Cl,^{35}Cl)+Na]$^+$); HRMS (ESI) found *m/z* 364.0390 [M(^{35}Cl)+Na]$^+$, $C_{15}H_{16}O_4SN^{35}ClNa^+$ requires *m/z* 364.0381.

tert-Butyl 2-(cyclohept-1-en-1-ylsulfonyl)acetate (3y)

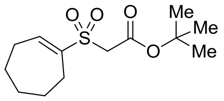

The general procedure was followed with the use of 1-iodocyclohept-1-ene (67 mg, 0.30 mmol). Flash column chromatography (5–20% Et_2O in petrol) afforded the titled *sulfone* **3y** as a yellow oil (58 mg, 70%); ^{1}H NMR (400 MHz, $CDCl_3$) δ 7.13 (1H, t, *J* = 6.5, C=C*H*), 3.83 (2H, s, SO_2C*H*$_2$$CO_2$), 2.61–2.56 (2H, m, C*H*$_2$), 2.38 (2H, app. q, *J* = 6.0, C*H*$_2$), 1.84–1.78 (2H, m, C*H*$_2$), 1.72–1.65 (2H, m, C*H*$_2$), 1.64–1.57 (2H, m, C*H*$_2$), 1.46 (9H, s, C*Me*$_3$); ^{13}C (100 MHz, $CDCl_3$) δ 161.6, 145.9, 142.6, 83.3, 58.5, 31.1, 28.7, 27.9, 27.8, 25.9, 25.2; IR υ_{max} (neat)/cm^{-1} 2929, 1730 (C=O), 1449, 1395, 1369, 1307 (SO_2), 1132 (SO_2); LRMS (ESI) *m/z* 297 (80%, [M+Na]$^+$), 571 (100%, [2M+Na]$^+$); HRMS (ESI) found *m/z* 297.1133 [M+Na]$^+$, $C_{13}H_{22}O_4SNa^+$ requires *m/z* 297.1131.

tert-Butyl 2-((cyclohexylidenemethyl)sulfonyl)acetate (3z)

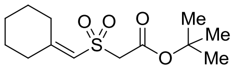

The general procedure was followed with the use of (iodomethylene)cyclohexane[7] (67 mg, 0.30 mmol). Flash column chromatography (5–20% Et_2O in petrol) afforded the titled *sulfone* **3z** as a yellow oil (31 mg, 38%); ^{1}H NMR (400 MHz, $CDCl_3$) δ 6.15 (1H, s, CC*H*SO_2), 3.88 (2H, s, SO_2C*H*$_2$$CO_2$), 2.73 (2H, t, *J* = 6.0, C*H*$_2$CCH), 2.25 (2H, t, *J* = 6.0, C*H*$_2$CCH), 1.75–1.65 (4H, m, 2 × C*H*$_2$), 1.65–1.58 (2H, m, C*H*$_2$), 1.49 (9H, s, C*Me*$_3$); ^{13}C (100 MHz, $CDCl_3$) δ 163.6, 161.9, 121.0, 83.5, 62.0, 37.6, 29.5, 28.3, 27.8, 27.5, 25.7; IR υ_{max} (neat)/cm^{-1} 2938, 1731, 1624, 1540, 1371, 1312 (SO_2), 1133 (SO_2); LRMS (ESI) *m/z* 297 (100%, [M+Na]$^+$), 571 (30%, [2M+Na]$^+$); HRMS (ESI) found *m/z* 297.1126 [M+Na]$^+$, $C_{13}H_{22}O_4SNa^+$ requires *m/z* 297.1131.

1-(Benzylsulfonyl)-3,5-dimethylbenzene (4)

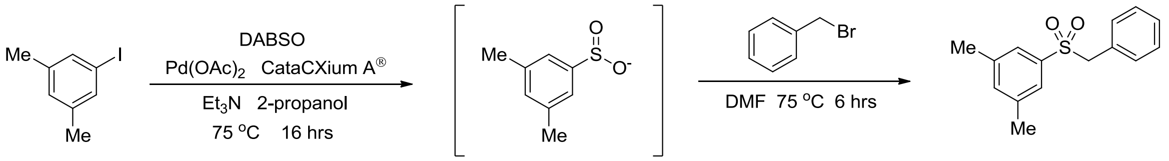

A glass reaction tube was charged with DABSO (43 mg, 0.18 mmol), palladium(II) acetate (4 mg, 15 µmol) and CataCXium A® (8 mg, 23 µmol), sealed with a rubber septum and evacuated and filled with nitrogen four times. 1-iodo-3,5-dimethylbenzene (44 µL, 0.30 mmol), anhydrous triethylamine (125 µL, 0.9 mmol) and anhydrous 2-propanol (1.5 mL) were added sequentially through the septum and the reaction mixture stirred under positive pressure of nitrogen in a preheated oil bath at 75 °C for 16 hrs. After cooling to room temperature, a solution of benzyl bromide (106 µL, 0.9 mmol) in anhydrous DMF (0.5 mL) was added dropwise and the resultant solution stirred at 75 °C for 6 hrs or until consumption of the sulfinate was observed by HPLC. Upon completion, the reaction mixture was poured onto water (20 mL) and extracted with EtOAc (3 × 20 mL). The combined organic fractions were dried ($MgSO_4$), filtered and concentrated *in vacuo*. Flash column chromatography (5–25% Et_2O in petrol) afforded the titled *sulfone* **4** as an off-white microcrystalline solid (66 mg, 85%); mp 75–76 °C (DCM); ^{1}H NMR (400 MHz, $CDCl_3$) δ 7.36–7.20 (6H, m, Ar-*H*), 7.13–7.09 (2H, m, Ar-*H*), 4.28 (2H, s, C*H₂*), 2.31 (6H, s, 2 × Ar-*Me*); ^{13}C (100 MHz, $CDCl_3$) δ 138.9, 137.5, 135.2, 130.8, 128.6, 128.4, 128.2, 126.1, 62.8, 21.0; IR υ_{max} (neat)/cm^{-1} 2922, 1457, 1291 (SO_2), 1260, 1155 (SO_2), 1128, 1105; LRMS (ESI) *m/z* 278 (90%, $[M+NH_4]^+$), 283 (30%, $[M+Na]^+$), 543 (100%, $[2M+Na]^+$); HRMS (ESI) found *m/z* 283.0764 $[M+Na]^+$, $C_{15}H_{16}O_2SNa^+$ requires *m/z* 283.0763.

1,3-Dimethyl-5-(phenylsulfonyl)benzene (5)

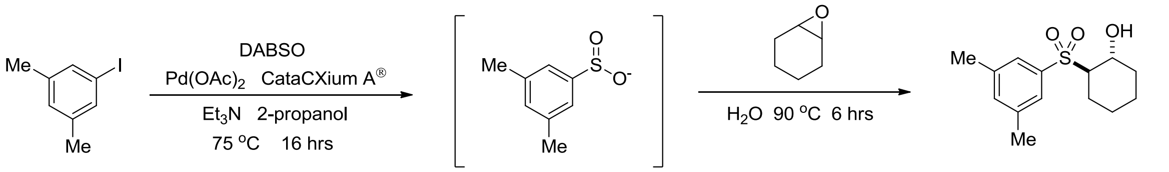

A glass reaction tube was charged with DABSO (43 mg, 0.18 mmol), palladium(II) acetate (4 mg, 15 µmol) and CataCXium A® (8 mg, 23 µmol), sealed with a rubber septum and evacuated and filled with nitrogen four times. 1-iodo-3,5-dimethylbenzene (44 µL, 0.30 mmol), anhydrous triethylamine (125 µL, 0.9 mmol) and anhydrous 2-propanol (1.5 mL) were added sequentially through the septum and the reaction mixture stirred under positive pressure of nitrogen in a preheated oil bath at 75 °C for 16 hrs. After cooling to room temperature, the reaction mixture was concentrated *in vacuo*. H_2O (1.5 mL) and cyclohexene oxide (300 µL, 3.0 mmol) were added and the resultant suspension stirred at 90 °C for 6 hrs or until consumption of the sulfinate was observed by HPLC. Upon completion, the reaction mixture was poured onto $NH_4Cl_{(aq)}$ (20 mL) and extracted with DCM (3 × 20 mL). The

combined organic fractions were dried ($MgSO_4$), filtered and concentrated *in vacuo*. Flash column chromatography (20–40% Et_2O in petrol) afforded the titled *sulfone* **5** as an off-white microcrystalline solid (61 mg, 76%); mp 126–127 °C (DCM); ^{1}H NMR (400 MHz, $CDCl_3$) δ 7.49 (2H, s, Ar-*H*), 7.30 (1H, s, Ar-*H*), 4.37 (1H, br. s, O*H*), 3.93 (1H, app. td, *J* = 10.0, 5.0, C*H*OH), 2.96 (1H, ddd, *J* = 12.5, 9.5, 4.0, C*H*SO_2), 2.41 (6H, s, 2 × Ar-*Me*), 2.17–2.10 (1H, m, CHOHC*H*H'), 1.92–1.85 (1H, m, $CHSO_2$C*H*H'), 1.76–1.67 (2H, m, CH_2), 1.40–1.10 (4H, m, CHOHCH*H'*, $CHSO_2$CH*H'* and CH_2); ^{13}C (100 MHz, $CDCl_3$) δ 139.3, 136.4, 135.8, 126.4, 68.8, 68.1, 34.1, 25.7, 24.5, 23.5, 21.2; IR υ_{max} (neat)/cm^{-1} 3513 (OH), 2944, 2867, 1609, 1445, 1271 (SO_2), 1133 (SO_2), 1104, 1065, 1041; LRMS (ESI) *m/z* 269 (40%, $[M+H]^+$), 286 (75%, $[M+NH_4]^+$), 291 (35%, $[M+Na]^+$), 559 (100%, $[2M+Na]^+$); HRMS (ESI) found *m/z* 291.1023 $[M+Na]^+$, $C_{14}H_{20}O_3SNa^+$ requires *m/z* 291.1025.

1,3-Dimethyl-5-(phenylsulfonyl)benzene (6)

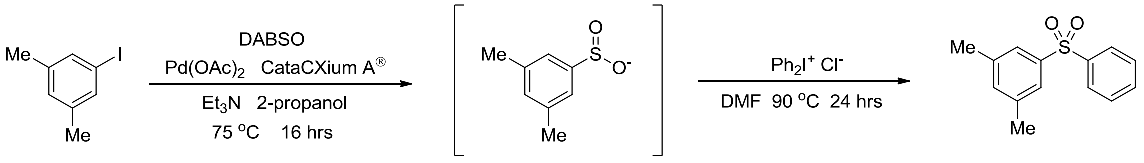

A glass reaction tube was charged with DABSO (43 mg, 0.18 mmol), palladium(II) acetate (4 mg, 15 µmol) and CataCXium A® (8 mg, 23 µmol), sealed with a rubber septum and evacuated and filled with nitrogen four times. 1-iodo-3,5-dimethylbenzene (44 µL, 0.30 mmol), anhydrous triethylamine (125 µL, 0.9 mmol) and anhydrous 2-propanol (1.5 mL) were added sequentially through the septum and the reaction mixture stirred under positive pressure of nitrogen in a preheated oil bath at 75 °C for 16 hrs. After cooling to room temperature, the reaction mixture was concentrated *in vacuo*. Diphenyl iodonium chloride (190 mg, 0.6 mmol) and DMF (1.5 mL) were added and the resultant suspension stirred at 90 °C for 24 hrs or until consumption of the sulfinate was observed by HPLC. Upon completion, the reaction mixture was poured onto water (20 mL) and extracted with Et_2O (3 × 20 mL). The combined organic fractions were dried ($MgSO_4$), filtered and concentrated *in vacuo*. Flash column chromatography (5–20% Et_2O in petrol) afforded the titled *sulfone* **6** as an off-white microcrystalline solid (56 mg, 76%); mp 87–88 °C (DCM); ^{1}H NMR (400 MHz, $CDCl_3$) δ 7.97–7.93 (2H, m, Ar-*H*), 7.59–7.47 (5H, m, Ar-*H*), 7.17 (1H, s, Ar-*H*), 2.36 (6H, s, 2 × Ar-*Me*); ^{13}C (100 MHz, $CDCl_3$) δ 141.8, 141.2, 139.3, 134.9, 133.0, 129.2, 127.5, 125.1, 21.2; IR υ_{max} (neat)/cm^{-1} 1608, 1447, 1299 (SO_2), 1149 (SO_2), 1112, 1082; LRMS (ESI) *m/z* 247 (35%, $[M+H]^+$), 264 (95%, $[M+NH_4]^+$), 269 (55%, $[M+Na]^+$), 515 (100%, $[2M+Na]^+$); HRMS (ESI) found *m/z* 269.0606 $[M+Na]^+$, $C_{14}H_{14}O_2SNa^+$ requires *m/z* 269.0607.

2-((3,5-Dimethylphenyl)sulfonyl)benzo[d]thiazole (7)

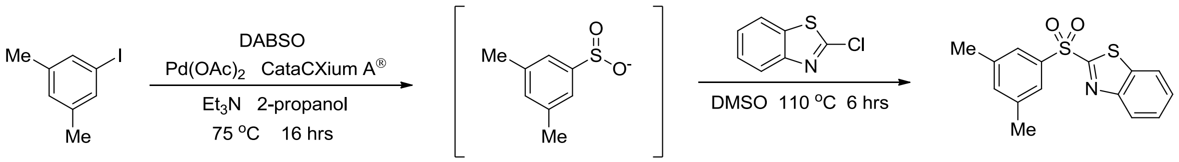

A glass reaction tube was charged with DABSO (43 mg, 0.18 mmol), palladium(II) acetate (4 mg, 15 µmol) and CataCXium A® (8 mg, 23 µmol), sealed with a rubber septum and evacuated and filled with nitrogen four times. 1-iodo-3,5-dimethylbenzene (44 µL, 0.30 mmol), anhydrous triethylamine (125 µL, 0.9 mmol) and anhydrous 2-propanol (1.5 mL) were added sequentially through the septum and the reaction mixture stirred under positive pressure of nitrogen in a preheated oil bath at 75 °C for 16 hrs. After cooling to room temperature, the reaction mixture was concentrated *in vacuo*. DMSO (1.5 mL) and 2-chlorobenzothiazole (117 µL, 0.9 mmol) were added and the resultant solution stirred at 100 °C for 6 hrs or until consumption of the sulfinate was observed by HPLC. Upon completion, the reaction mixture was diluted with EtOAc (30 mL) and washed with brine (3 × 20 mL). The organic layer was dried ($MgSO_4$), filtered and concentrated *in vacuo*. Flash column chromatography (15–50% Et_2O in petrol) afforded the titled *sulfone* **7** as an off-white microcrystalline solid (50 mg, 55%); mp 175–176 °C (DCM); ^{1}H NMR (400 MHz, $CDCl_3$) δ 8.17 (1H, d, *J* = 8.0, Ar-*H*), 7.96 (1H, d, *J* = 8.0, Ar-*H*), 7.77 (2H, s, Ar-*H*), 7.56 (2H, overlapping td, *J* = 7.0, 1.5, Ar-*H*), 7.27 (1H, s, Ar-*H*), 2.40 (6H, s, 2 × Ar-*Me*); ^{13}C (100 MHz, $CDCl_3$) δ 167.6, 152.9, 139.7, 138.1, 137.1, 136.3, 127.8, 127.4, 126.4, 125.5, 122.2, 21.2; IR υ_{max} (neat)/cm^{-1} 1468, 1334 (SO_2), 1305, 1270, 1152 (SO_2), 1107, 1023; LRMS (ESI) *m/z* 304 (90%, $[M+H]^+$), 326 (80%, $[M+Na]^+$), 629 (100%, $[2M+Na]^+$); HRMS (ESI) found *m/z* 326.0285 $[M+Na]^+$, $C_{15}H_{13}O_2S_2NNa^+$ requires *m/z* 326.0280.

3,5-Dimethylbenzene-1-sulfonyl chloride (8)

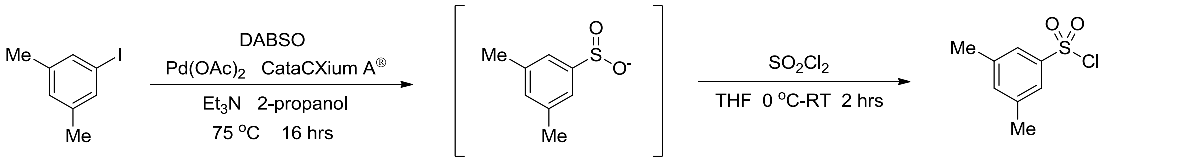

A glass reaction tube was charged with DABSO (43 mg, 0.18 mmol), palladium(II) acetate (4 mg, 15 µmol) and CataCXium A® (8 mg, 23 µmol), sealed with a rubber septum and evacuated and filled with nitrogen four times. 1-iodo-3,5-dimethylbenzene (44 µL, 0.30 mmol), anhydrous triethylamine (125 µL, 0.9 mmol) and anhydrous 2-propanol (1.5 mL) were added sequentially through the septum and the reaction mixture stirred under positive pressure of nitrogen in a preheated oil bath at 75 °C for 16 hrs. After cooling to room temperature, the reaction mixture was concentrated *in vacuo*. THF (1.5 mL) was added, the resultant suspension cooled to 0 °C and sulfuryl chloride (73 µL, 0.9 mmol) added dropwise. The reaction mixture was allowed to warm to room temperature and stirred for 2 hrs

or until consumption of the sulfinate was observed by HPLC. Upon completion, the reaction mixture was poured onto NH_4Cl (20 mL) and extracted with DCM (3 × 20 mL). The combined organic fractions were dried ($MgSO_4$), filtered and concentrated *in vacuo*. Flash column chromatography (0–5% Et_2O in petrol) afforded the titled *sulfonyl chloride* **8** as a white microcrystalline solid (37 mg, 60%); mp 89–90 °C (DCM); ^{1}H NMR (400 MHz, $CDCl_3$) δ 7.66 (2H, s, Ar-*H*), 7.36 (1H, s, Ar-*H*), 2.45 (6H, s, 2 × Ar-*Me*); ^{13}C (100 MHz, $CDCl_3$) δ 144.2, 140.0, 136.9, 124.4, 21.2; IR υ_{max} (neat)/cm^{-1} 1608, 1445, 1364 (SO_2), 1309, 1267, 1165 (SO_2), 1106; HRMS (FI) found *m/z* 204.0009 [M(^{35}Cl)]$^+$, $C_8H_9O_2S^{35}Cl^+$ requires *m/z* 204.0012.

4-((3,5-Dimethylphenyl)sulfonyl)morpholine (9)

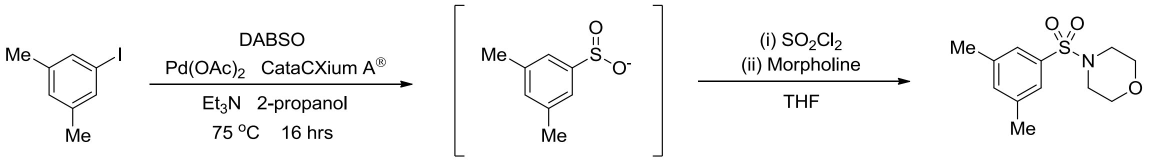

A glass reaction tube was charged with DABSO (43 mg, 0.18 mmol), palladium(II) acetate (4 mg, 15 µmol) and CataCXium A® (8 mg, 23 µmol), sealed with a rubber septum and evacuated and filled with nitrogen four times. 1-iodo-3,5-dimethylbenzene (44 µL, 0.30 mmol), anhydrous triethylamine (125 µL, 0.9 mmol) and anhydrous 2-propanol (1.5 mL) were added sequentially through the septum and the reaction mixture stirred under positive pressure of nitrogen in a preheated oil bath at 75 °C for 16 hrs. After cooling to room temperature, the reaction mixture was concentrated *in vacuo*. THF (1.5 mL) was added, the resultant suspension cooled to 0 °C and sulfuryl chloride (73 µL, 0.9 mmol) added dropwise. The reaction mixture was allowed to warm to room temperature and stirred for 2 hrs or until consumption of the sulfinate was observed by HPLC. Morpholine (175 µL, 2 mmol) was added dropwise and the reaction mixture stirred for a further 2 hrs at room temperature or until consumption of the sulfonyl chloride was observed by HPLC. Upon completion, the reaction mixture was poured onto water (20 mL) and extracted with DCM (3 × 20 mL). The combined organic fractions were dried ($MgSO_4$), filtered and concentrated *in vacuo*. Flash column chromatography (20–60% Et_2O in petrol) afforded the titled *sulfonamide* **9** as an off-white microcrystalline solid (65 mg, 85%); mp 144–145 °C (DCM); ^{1}H NMR (400 MHz, $CDCl_3$) δ 7.35 (2H, s, Ar-*H*), 7.24 (1H, s, Ar-*H*), 3.75 (4H, t, *J* = 4.5, N(CH$_2$C*H*$_2$)$_2$O), 2.99 (4H, t, *J* = 4.5, N(C*H*$_2$CH$_2$)$_2$O), 2.40 (6H, s, 2 × Ar-*Me*); ^{13}C (100 MHz, $CDCl_3$) δ 139.1, 134.74, 134.65, 125.3, 66.1, 46.0, 21.2; IR υ_{max} (neat)/cm^{-1} 2857, 1607, 1455, 1343 (SO_2), 1328, 1296, 1261, 1158 (SO_2), 1112, 1066; LRMS (ESI) *m/z* 256 (100%, [M+H]$^+$), 278 (80%, [M+Na]$^+$), 533 (45%, [2M+Na]$^+$); HRMS (ESI) found *m/z* 278.0824 [M+Na]$^+$, $C_{12}H_{17}O_3SNNa^+$ requires *m/z* 278.0821.

1,2-Bis(3,5-dimethylphenyl)disulfane (10)

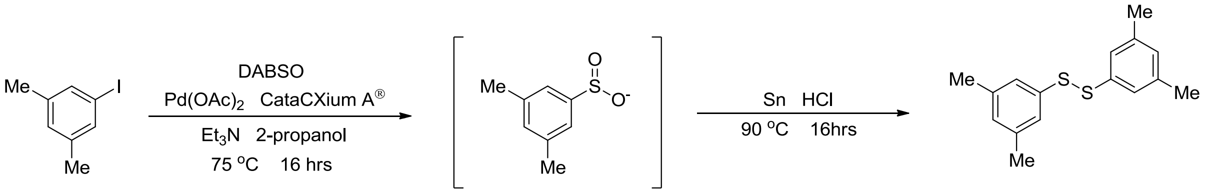

A glass reaction tube was charged with DABSO (43 mg, 0.18 mmol), palladium(II) acetate (4 mg, 15 µmol) and CataCXium A® (8 mg, 23 µmol), sealed with a rubber septum and evacuated and filled with nitrogen four times. 1-iodo-3,5-dimethylbenzene (44 µL, 0.30 mmol), anhydrous triethylamine (125 µL, 0.9 mmol) and anhydrous 2-propanol (1.5 mL) were added sequentially through the septum and the reaction mixture stirred under positive pressure of nitrogen in a preheated oil bath at 75 ^{o}C for 16 hrs. After cooling to room temperature, the reaction mixture was concentrated *in vacuo*. Powdered elemental tin (1.0 g, 8.5 mmol) and water (1 mL) were added, then conc. $HCl_{(aq)}$ was added dropwise. The resultant suspension was stirred at 90 ^{o}C for 16hrs or until consumption of the sulfinate was observed by HPLC. Upon completion, the reaction mixture was diluted with water (25 mL) and DCM (25 mL) and filtered through Celite. The organic layer was separated and the aqueous layer extracted with further DCM (2 x 30 mL). The combiner organic layers were dried ($MgSO_4$), filtered and concentrated *in vacuo*. Flash column chromatography (100% petrol) afforded the titled *disulfane* **10** as a colourless oil (29 mg, 70%); ^{1}H NMR (400 MHz, $CDCl_3$) δ 7.14 (4H, s, Ar-*H*), 6.87 (2H, s, Ar-*H*), 2.30 (12H, s, 4 × Ar-*Me*); ^{13}C (100 MHz, $CDCl_3$) δ 138.7, 136.8, 129.0, 125.1, 21.2; HRMS (FI) found *m/z* 274.0854 $[M]^+$, $C_{16}H_{18}S_2^+$ requires *m/z* 274.0850. Data consistent with literature reported values.[8]

3. References

1. B. Nguyen, E. J. Emmett, M. C. Willis *J. Am. Chem. Soc.* **2010**, *132*, 16372-16373.
2. A. E. Gladwin (Glaxo Group Ltd.), WO2005040124 (A1), **2005**
3. A. Klapars, S. L. Buchwald *J. Am. Chem. Soc.* **2002**, *124*, 14844-14845.
4. K. Itami, K. Terakawa, J.-i. Yoshida, O. Kajimoto *J. Am. Chem. Soc.* **2003**, *125*, 6058-6059.
5. K. Lee, D. F. Wiemer *Tetrahedron Lett.* **1993**, *34*, 2433-2436.
6. S. J. Nara, M. Jha, J. Brinkhorst, T. J. Zemanek, D. A. Pratt *J. Org. Chem.* **2008**, *73*, 9326-9333.
7. Y.-Y. Lin, Y.-J. Wang, C.-H. Lin, J.-H. Cheng, C.-F. Lee *J. Org. Chem.* **2012**, *77*, 6100-6106.
8. A. Spurg, G. Schnakenburg, S. R. Waldvogel *Chem. -Eur. J.* **2009**, *15*, 13313-13317.

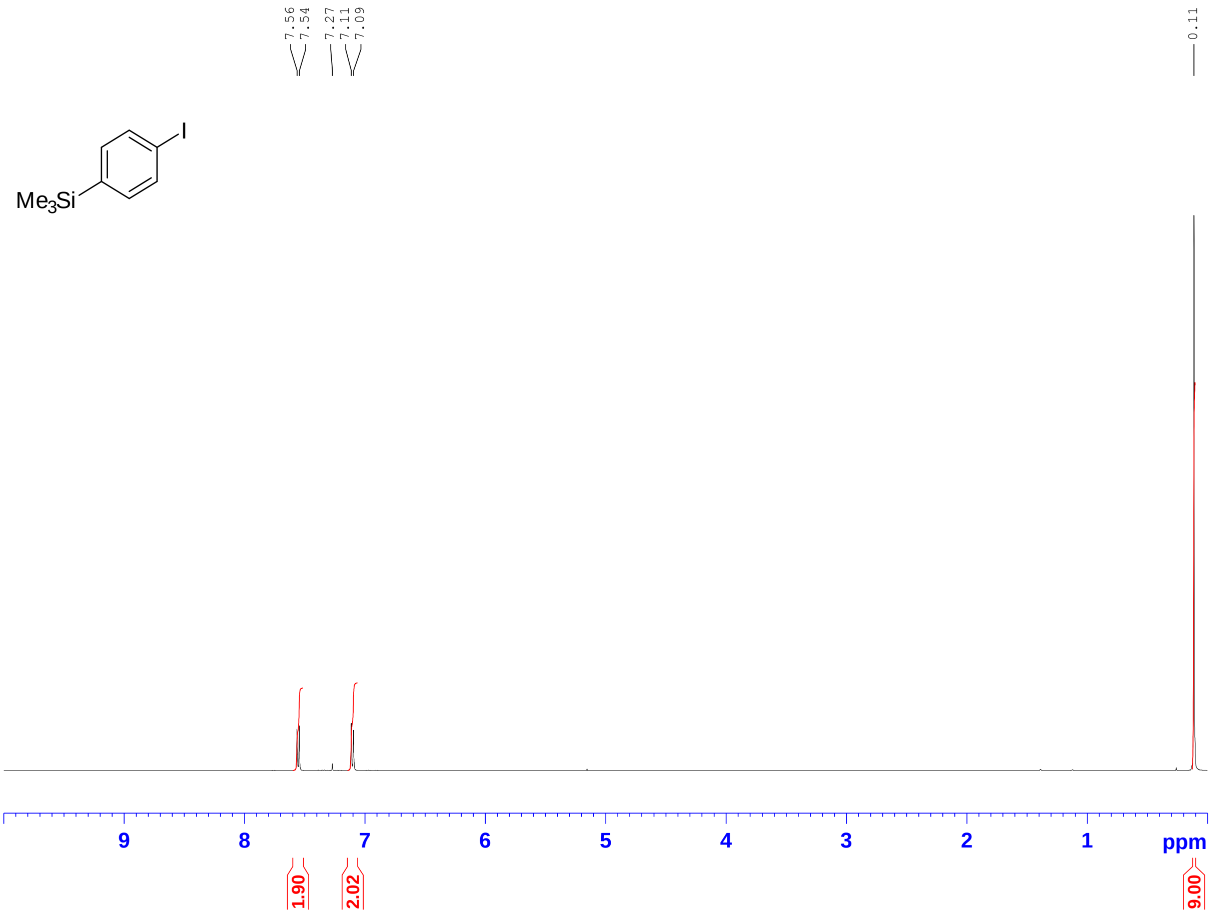
7.56
7.54
7.27
7.11
7.09
0.11
Me3Si
I
9
8
7
6
5
4
3
2
1
ppm
1.90
2.02
9.00

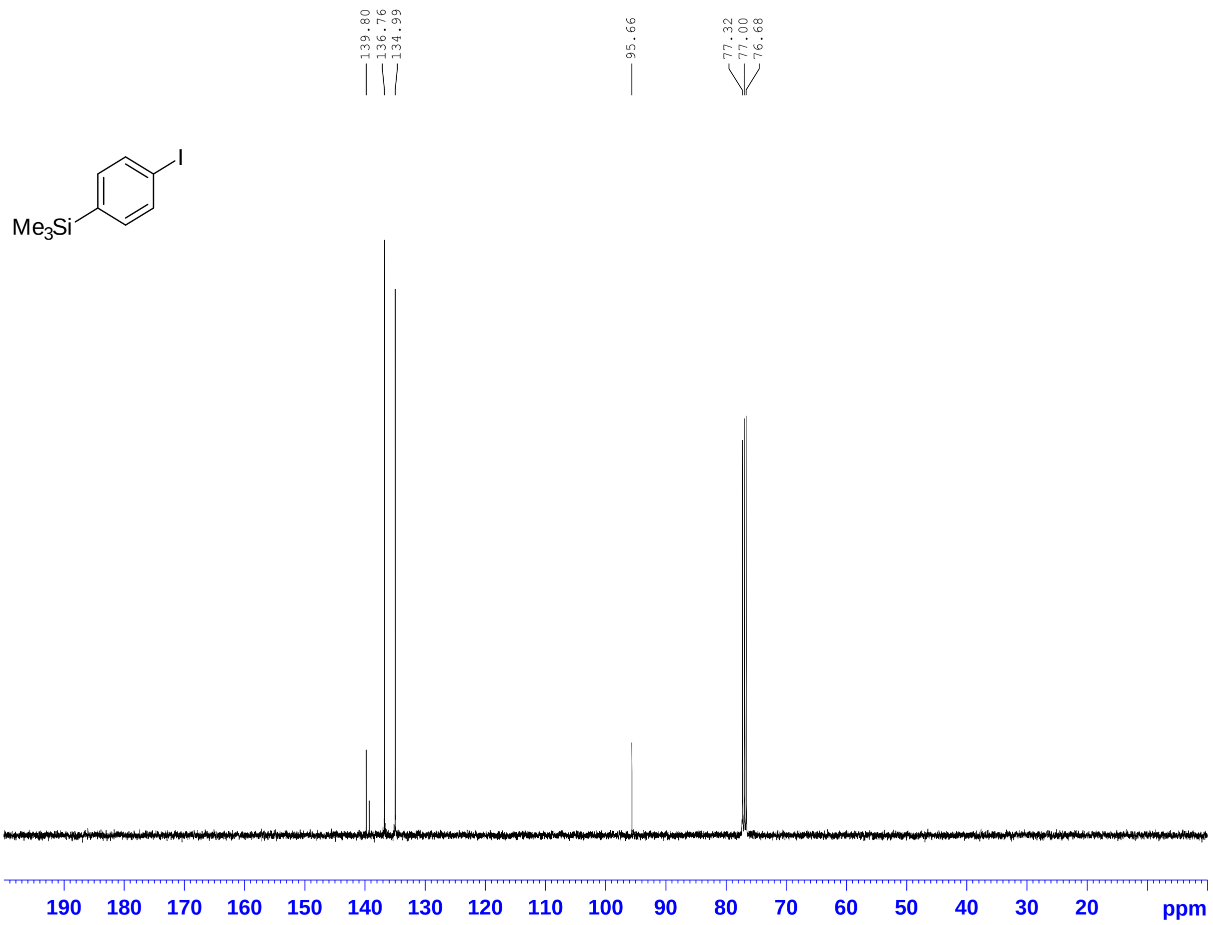
139.80
136.76
134.99
95.66
77.32
77.00
76.68
Me3Si
I
190
180
170
160
150
140
130
120
110
100
90
80
70
60
50
40
30
20
ppm

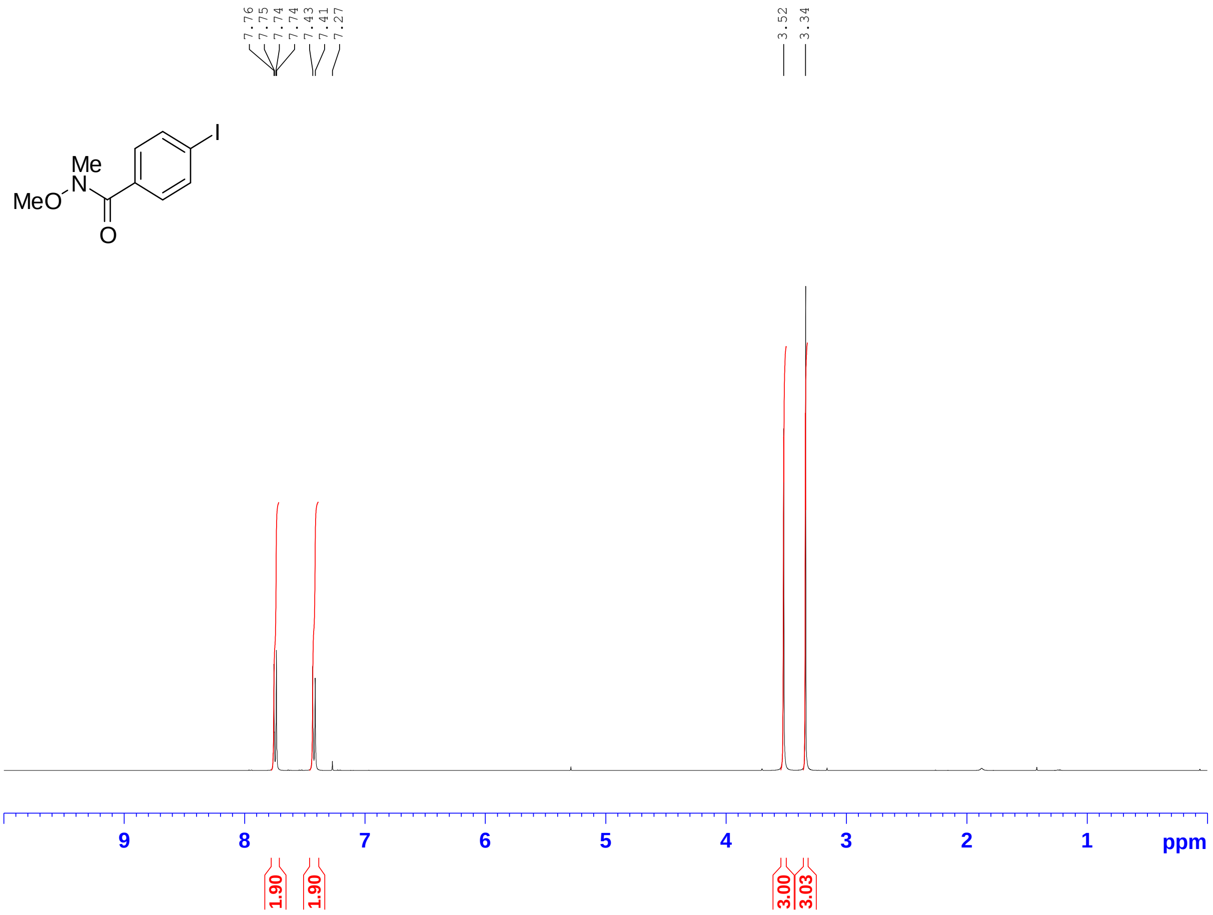
7.76
7.75
7.74
7.74
7.43
7.41
7.27
3.52
3.34
Me
N
MeO
O
I
1.90
1.90
3.00
3.03
ppm

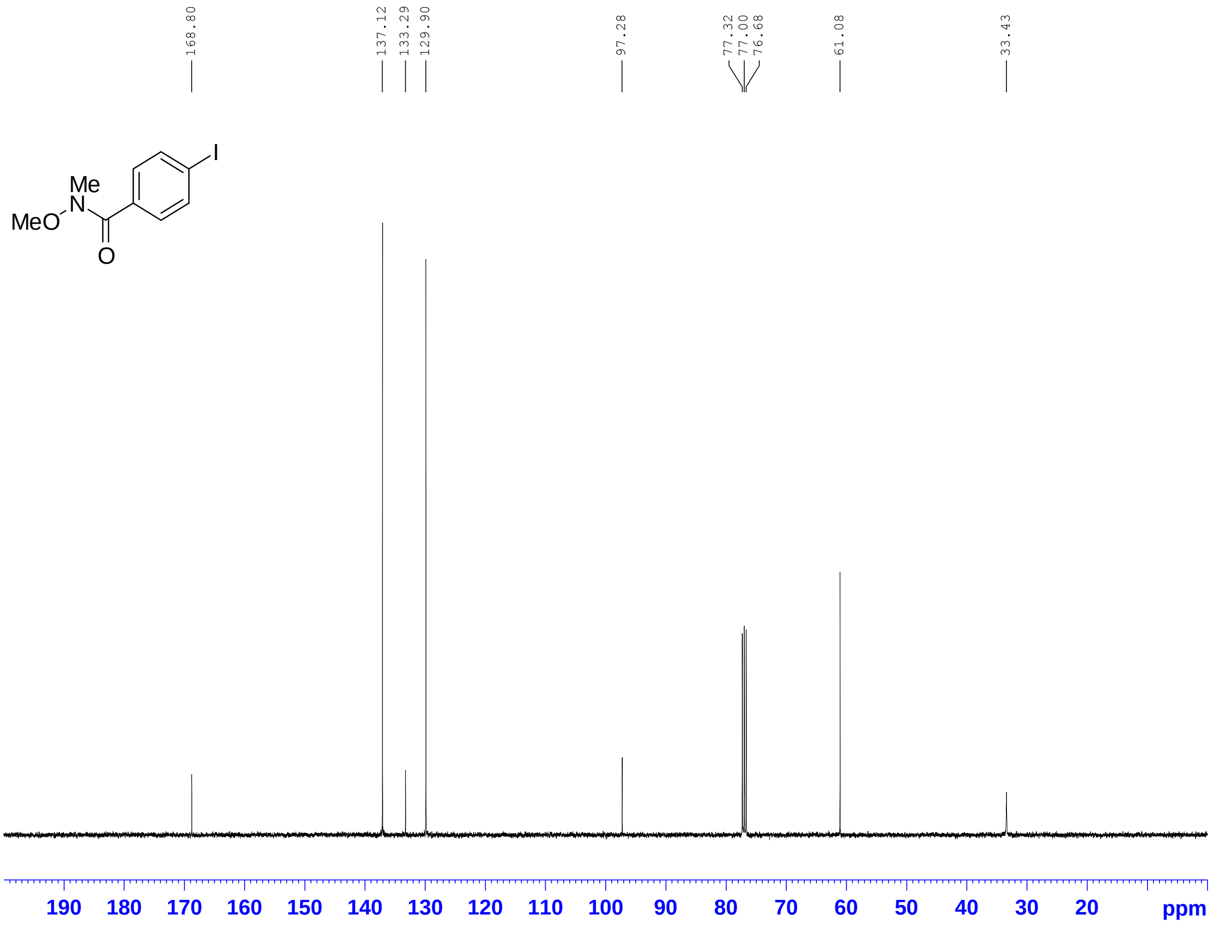
168.80
137.12
133.29
129.90
97.28
77.32
77.00
76.68
61.08
33.43
Me
N
MeO
O
I
ppm

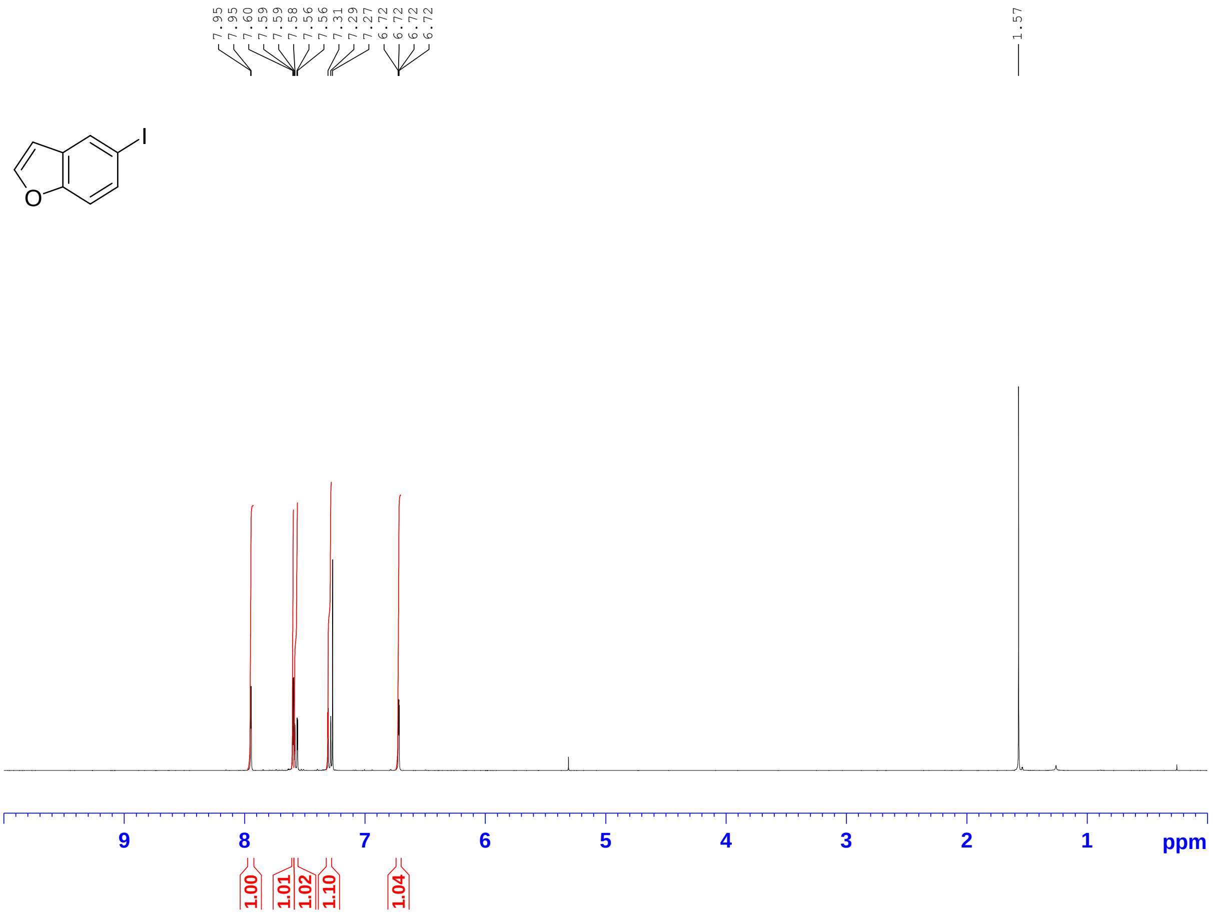
7.95
7.95
7.60
7.59
7.59
7.58
7.56
7.56
7.31
7.29
7.27
6.72
6.72
6.72
6.72
1.57
I
O
9 8 7 6 5 4 3 2 1 ppm
1.00 1.01 1.02 1.10 1.04

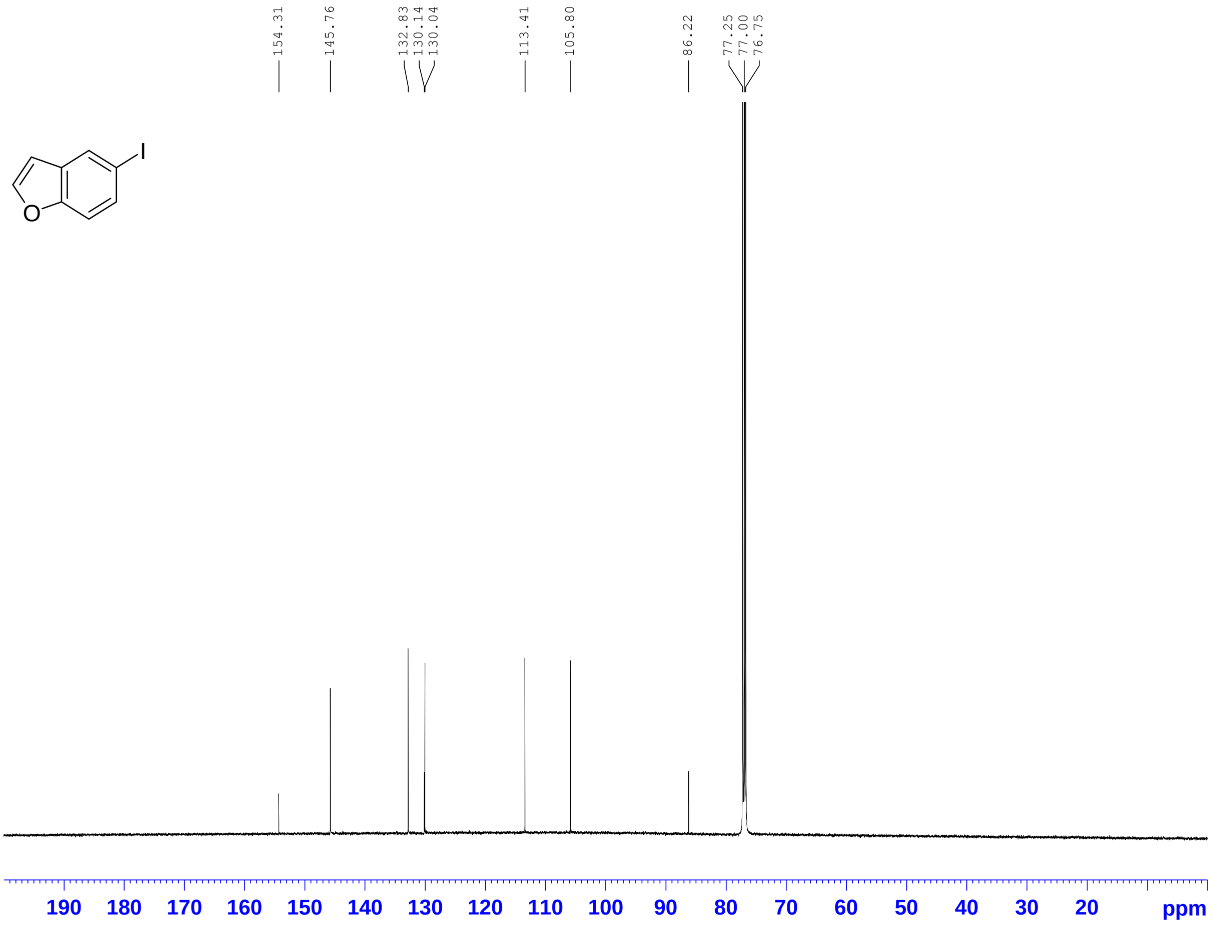
154.31
145.76
132.83
130.14
130.04
113.41
105.80
86.22
77.25
77.00
76.75
I
O
190 180 170 160 150 140 130 120 110 100 90 80 70 60 50 40 30 20 ppm

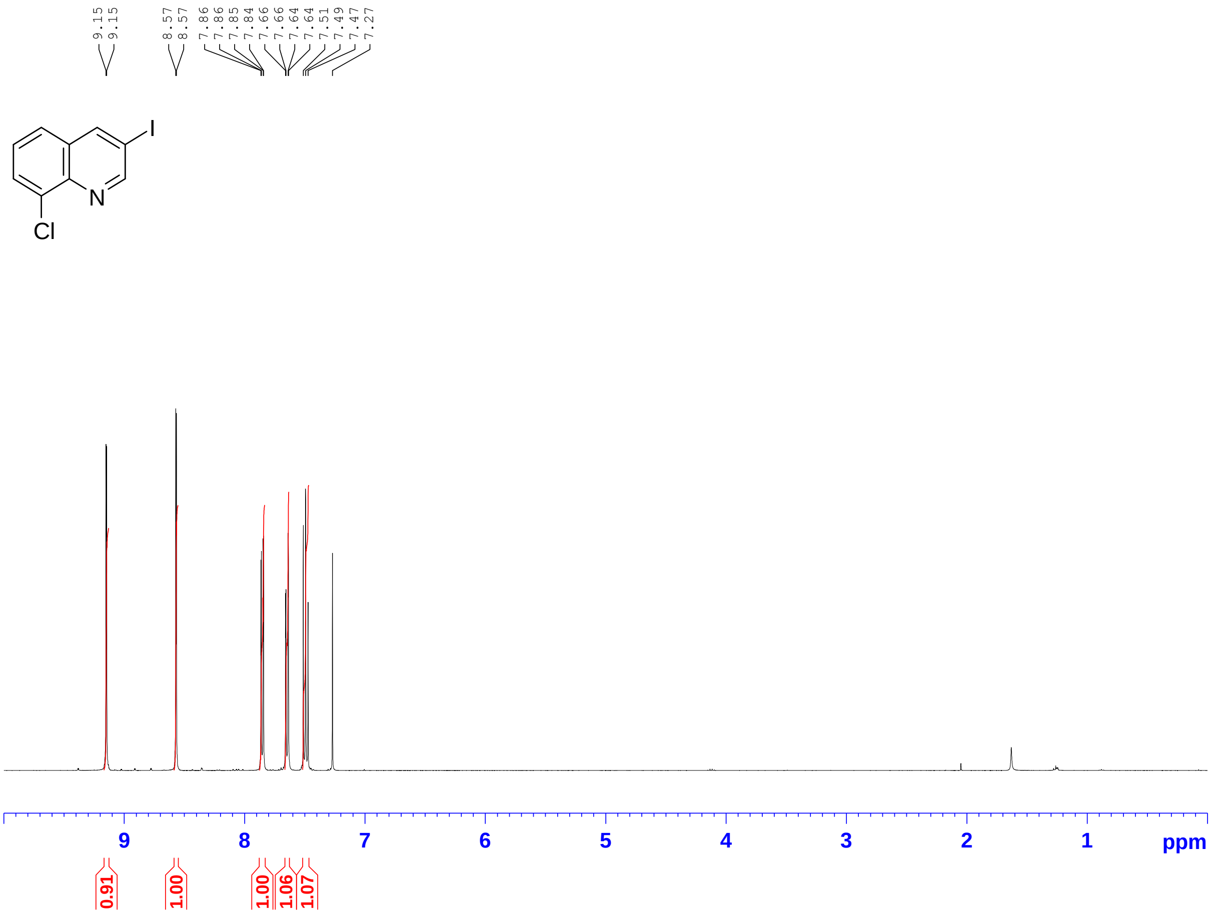
9.15
9.15
8.57
8.57
7.86
7.86
7.85
7.84
7.66
7.66
7.64
7.64
7.51
7.49
7.47
7.27
I
N
Cl
9
8
7
6
5
4
3
2
1
ppm
0.91
1.00
1.00
1.06
1.07

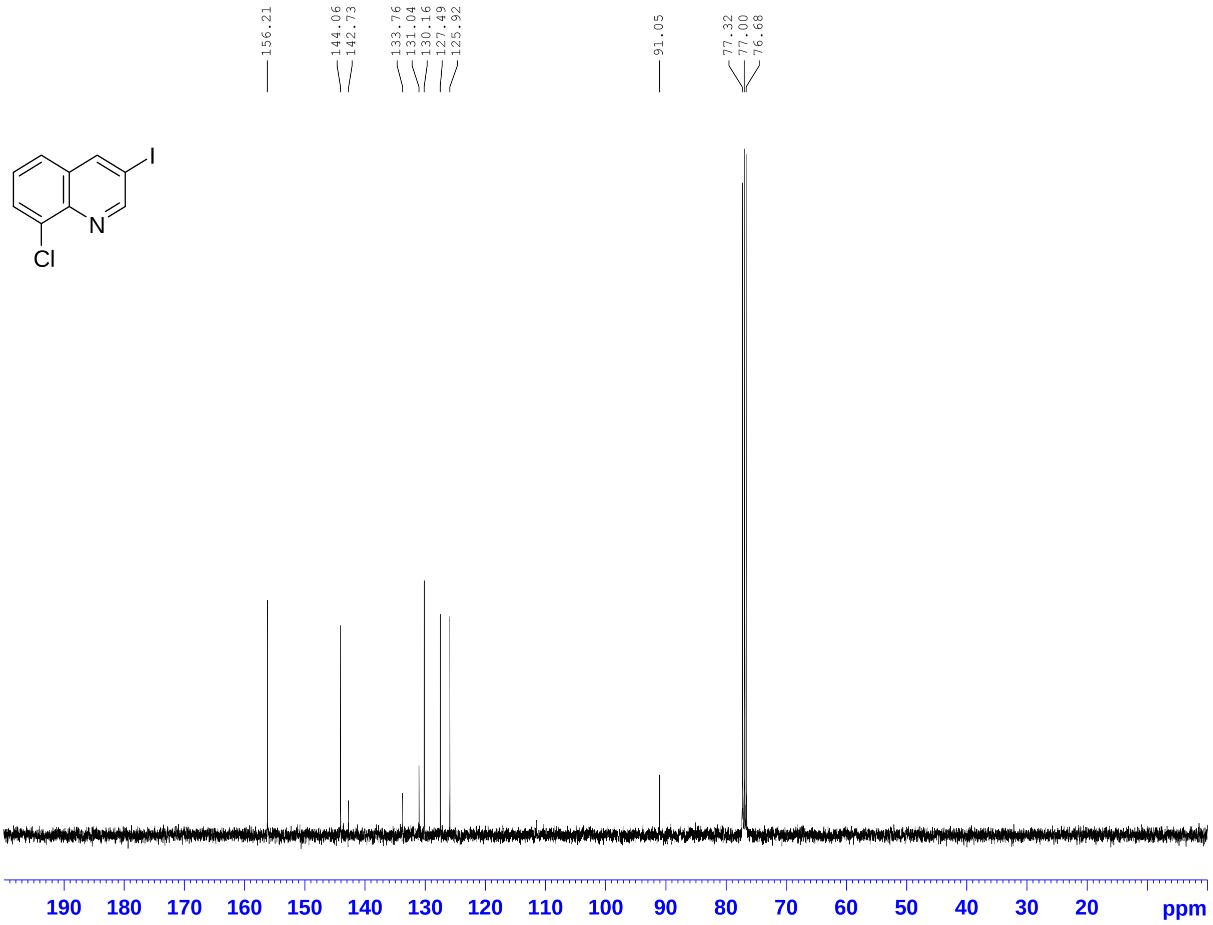
156.21
144.06
142.73
133.76
131.04
130.16
127.49
125.92
91.05
77.32
77.00
76.68
I
N
Cl
190
180
170
160
150
140
130
120
110
100
90
80
70
60
50
40
30
20
ppm

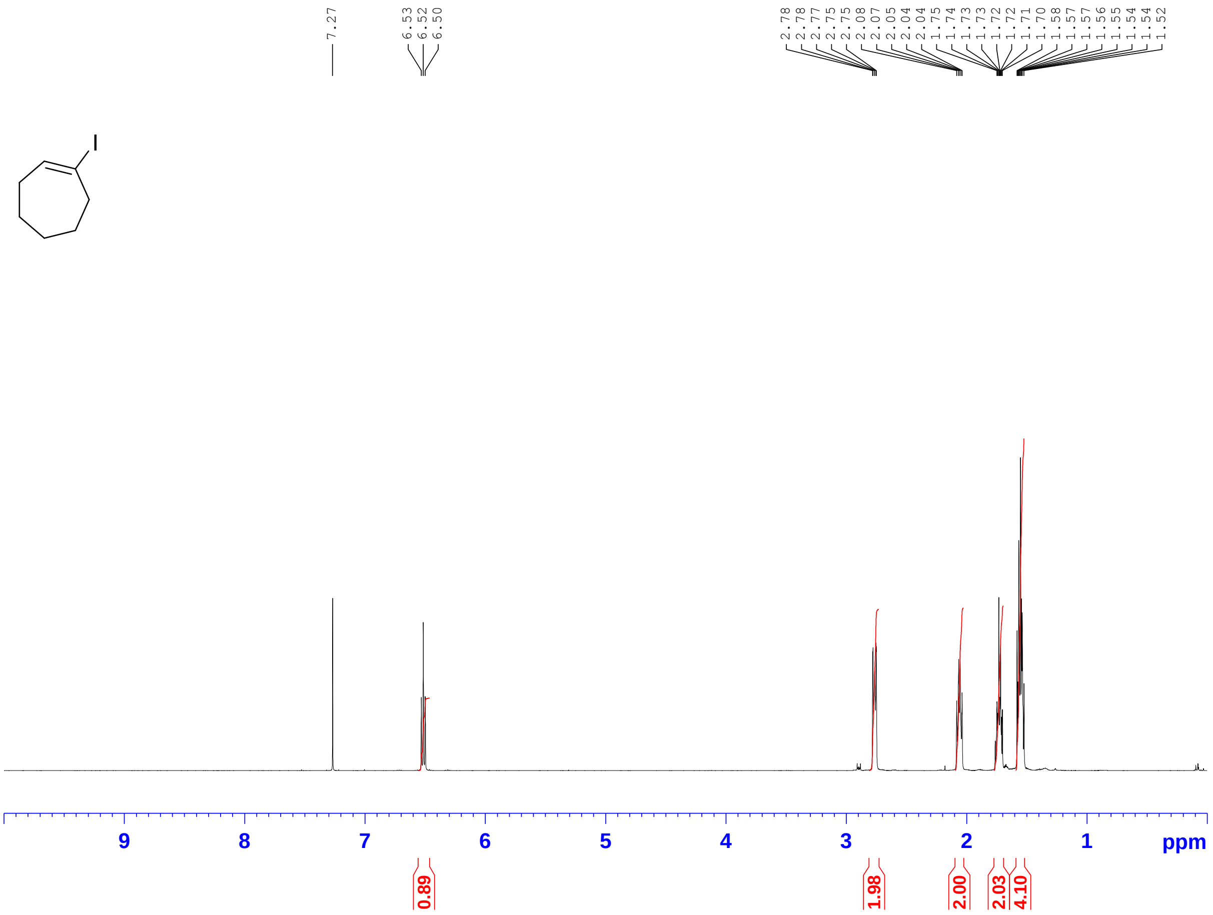

7.27
6.53
6.52
6.50
2.78
2.78
2.77
2.75
2.75
2.08
2.07
2.05
2.04
2.04
1.75
1.74
1.73
1.73
1.72
1.72
1.71
1.70
1.58
1.57
1.57
1.56
1.55
1.54
1.54
1.52
I
9
8
7
6
5
4
3
2
1
ppm
0.89
1.98
2.00
2.03
4.10

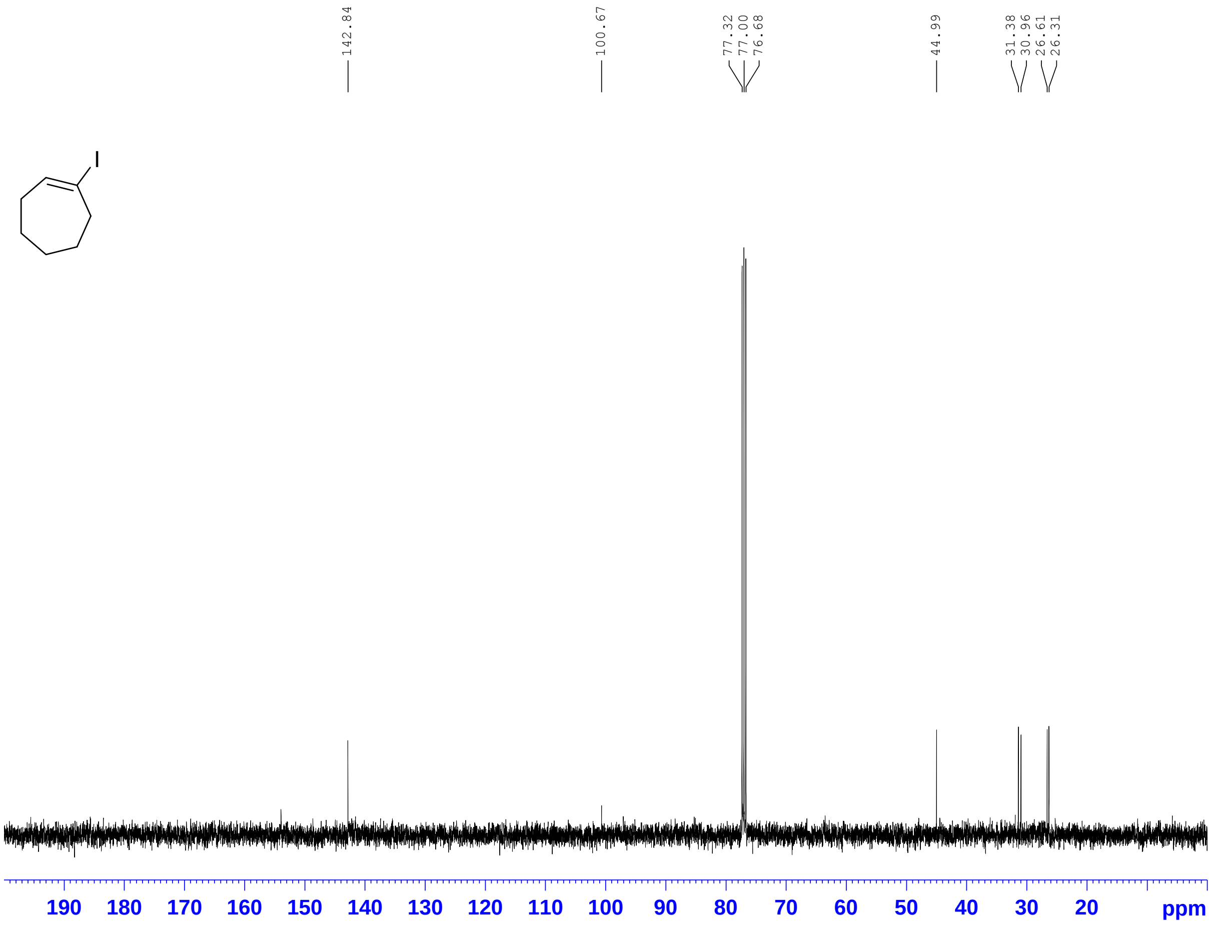

142.84
100.67
77.32
77.00
76.68
44.99
31.38
30.96
26.61
26.31
I
190
180
170
160
150
140
130
120
110
100
90
80
70
60
50
40
30
20
ppm

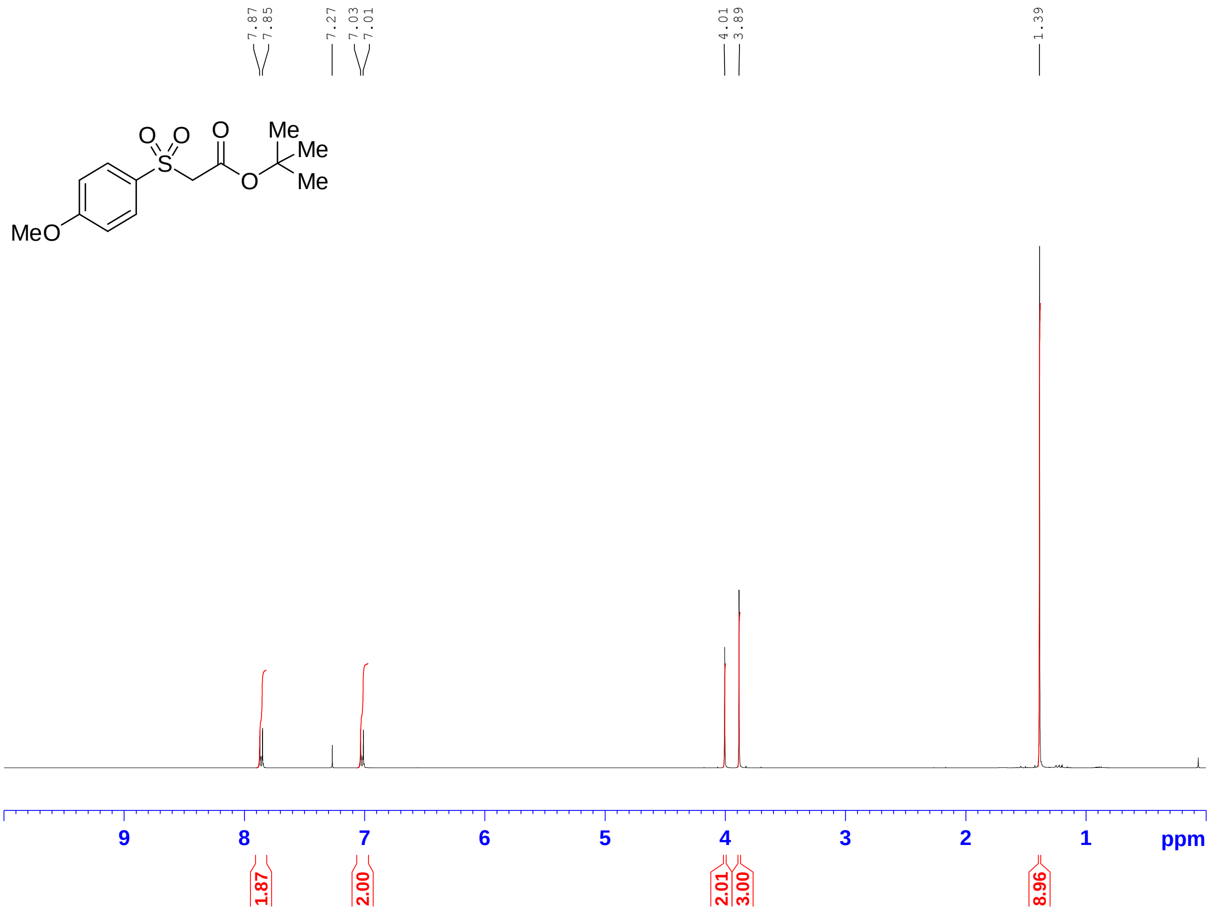
7.87
7.85
7.27
7.03
7.01
4.01
3.89
1.39
MeO
Me
ppm
1.87
2.00
2.01
3.00
8.96

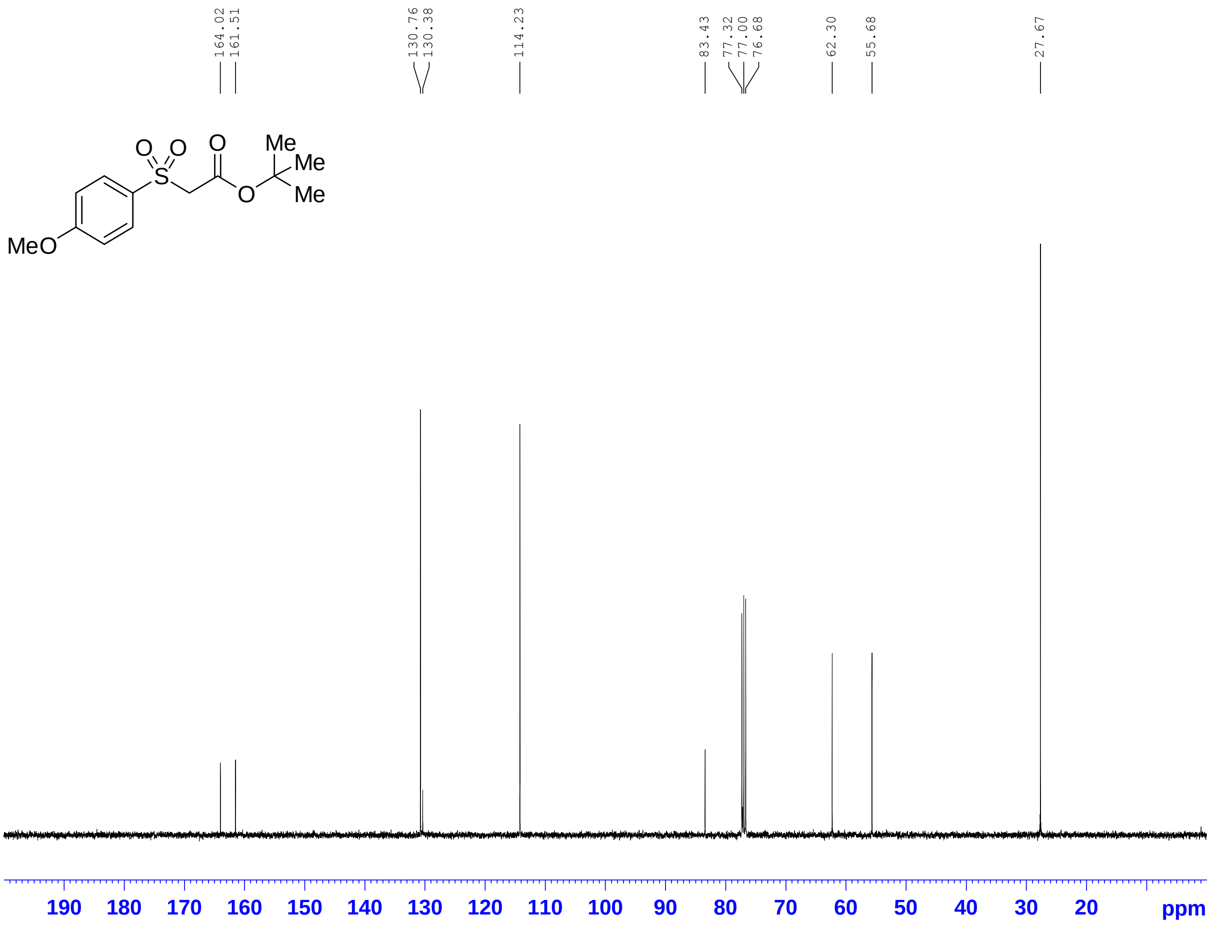
164.02
161.51
130.76
130.38
114.23
83.43
77.32
77.00
76.68
62.30
55.68
27.67
MeO
Me
ppm

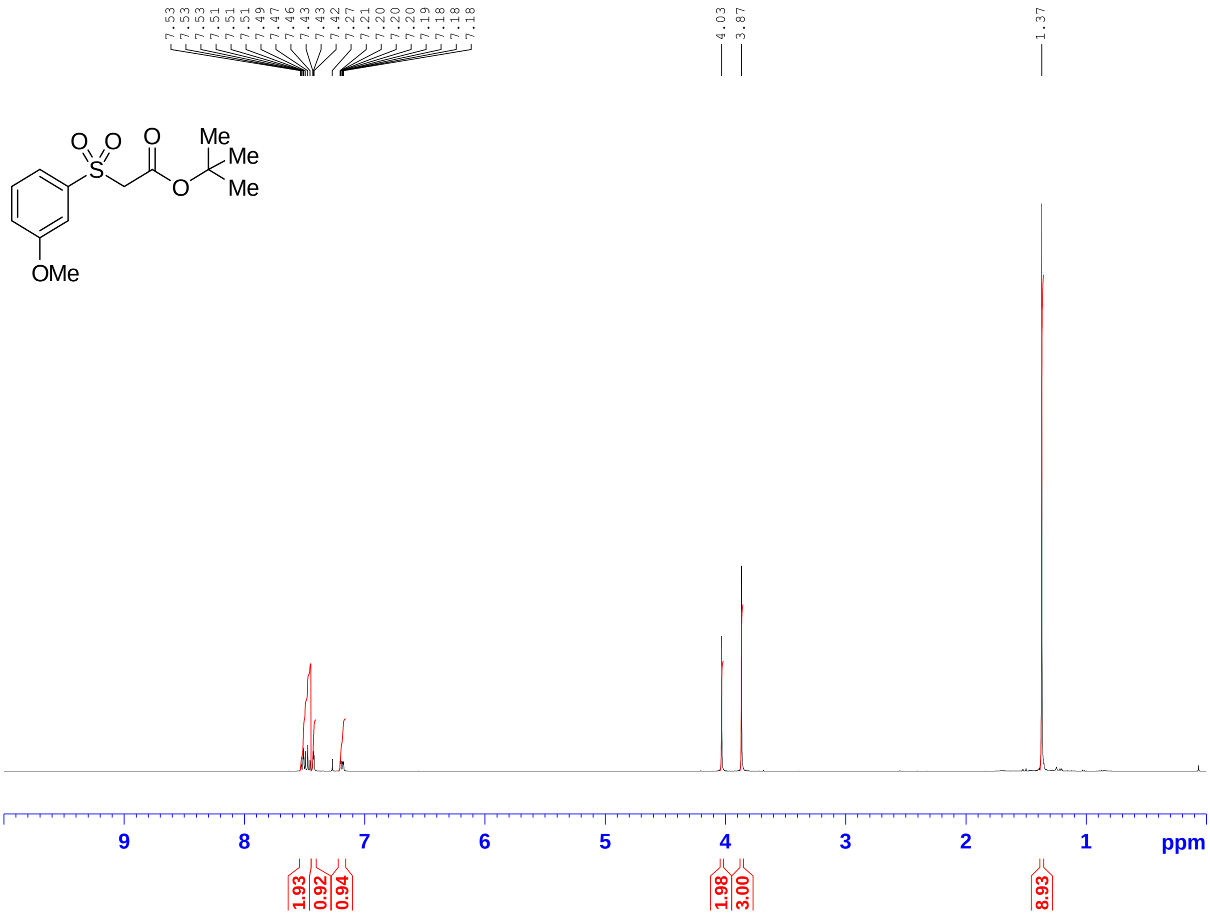

7.53
7.53
7.53
7.51
7.51
7.51
7.49
7.47
7.46
7.43
7.43
7.43
7.42
7.27
7.21
7.20
7.20
7.20
7.19
7.18
7.18
7.18
4.03
3.87
1.37
Me
Me
Me
OMe
9
8
7
6
5
4
3
2
1
ppm
1.93
0.92
0.94
1.98
3.00
8.93

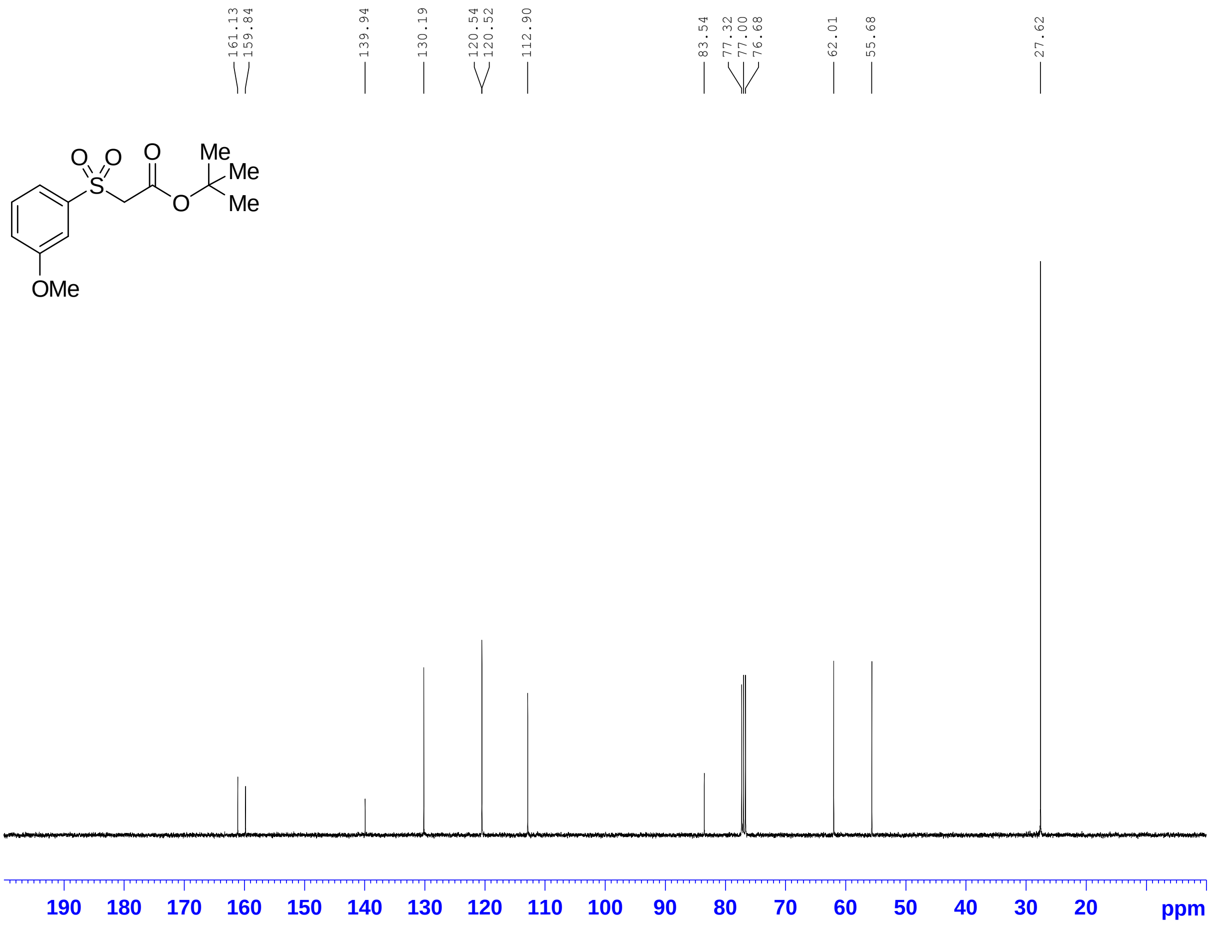

161.13
159.84
139.94
130.19
120.54
120.52
112.90
83.54
77.32
77.00
76.68
62.01
55.68
27.62
Me
Me
Me
OMe
190
180
170
160
150
140
130
120
110
100
90
80
70
60
50
40
30
20
ppm

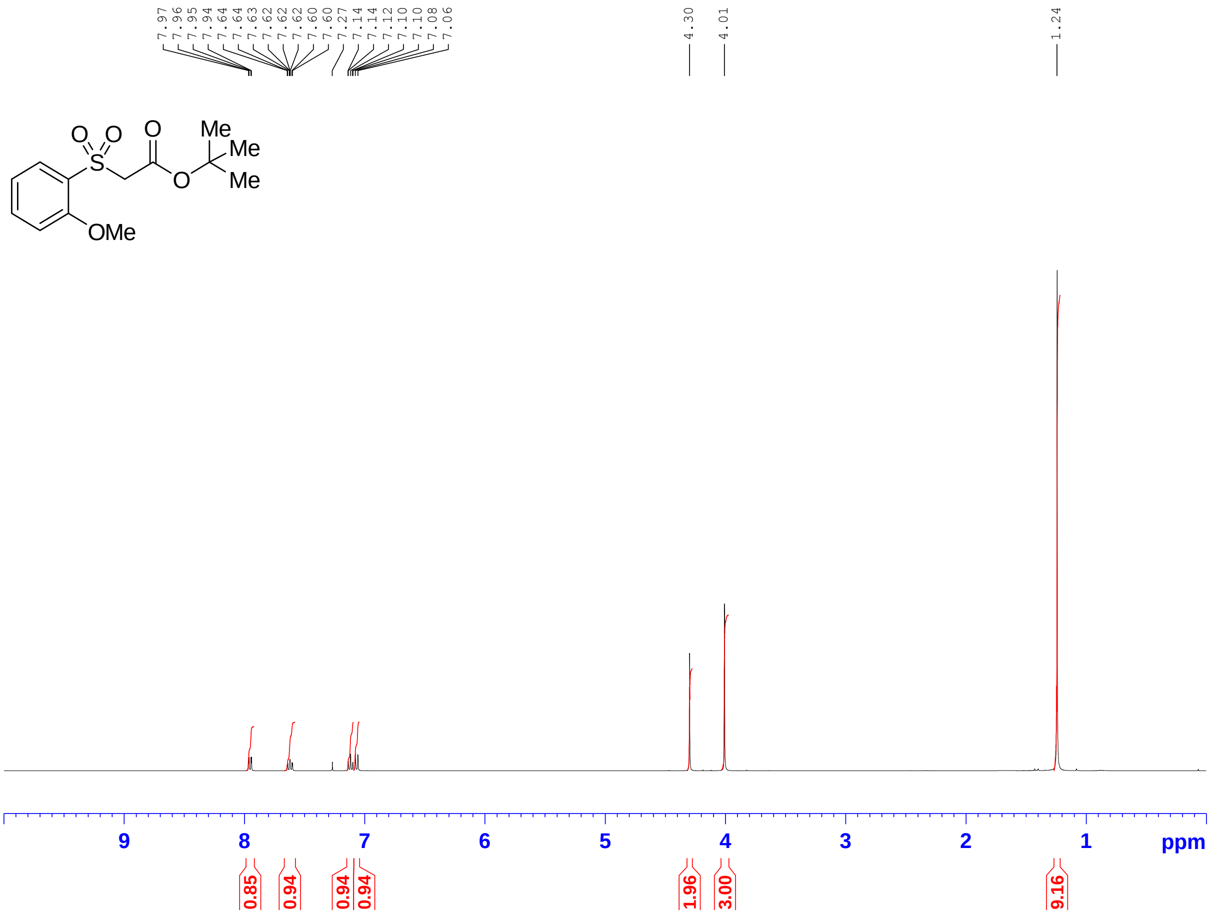
7.97
7.96
7.95
7.94
7.64
7.64
7.63
7.62
7.62
7.62
7.60
7.60
7.27
7.14
7.14
7.12
7.10
7.10
7.08
7.06
4.30
4.01
1.24
O
O
O
S
O
Me
Me
Me
OMe
9
8
7
6
5
4
3
2
1
ppm
0.85
0.94
0.94
0.94
1.96
3.00
9.16

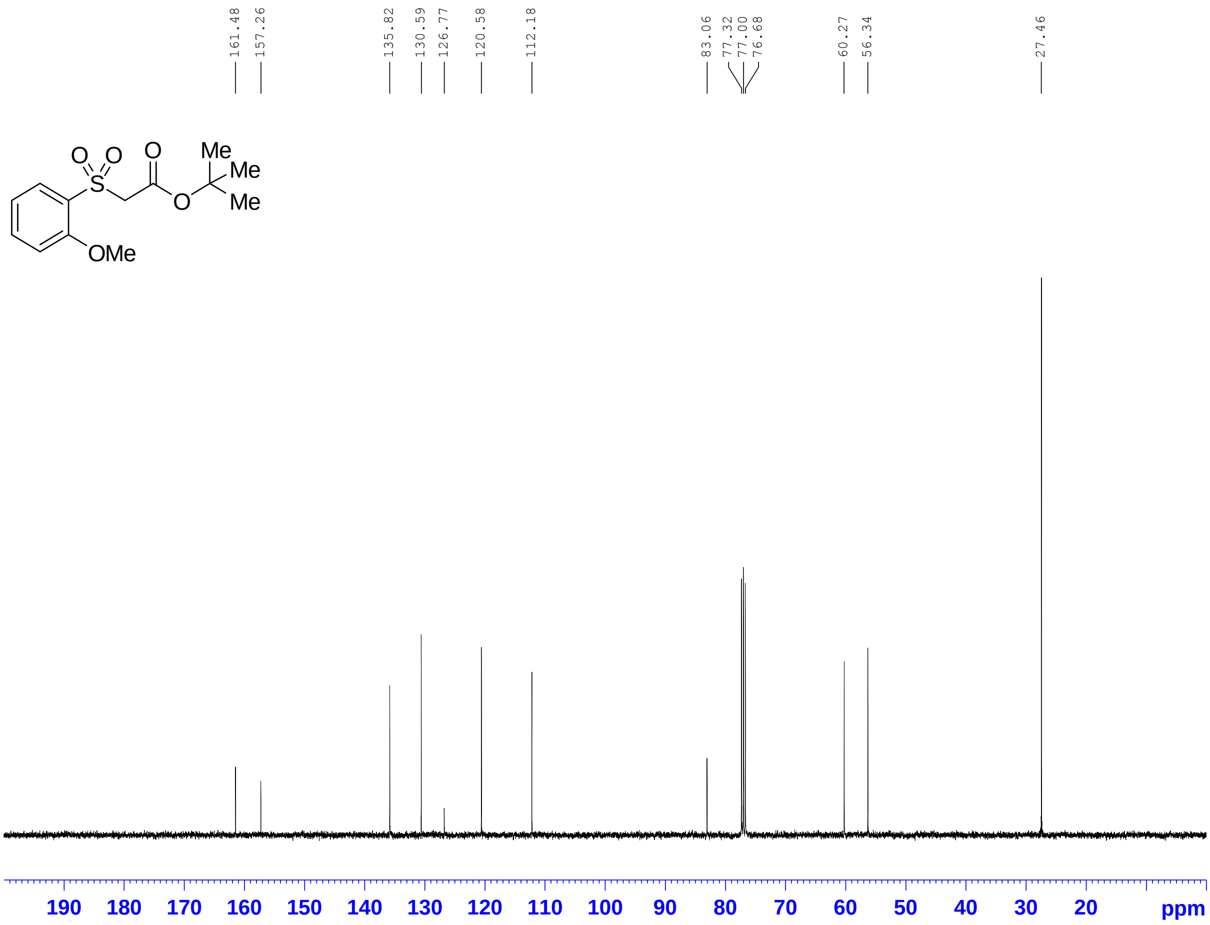
161.48
157.26
135.82
130.59
126.77
120.58
112.18
83.06
77.32
77.00
76.68
60.27
56.34
27.46
O
O
O
S
O
Me
Me
Me
OMe
190
180
170
160
150
140
130
120
110
100
90
80
70
60
50
40
30
20
ppm

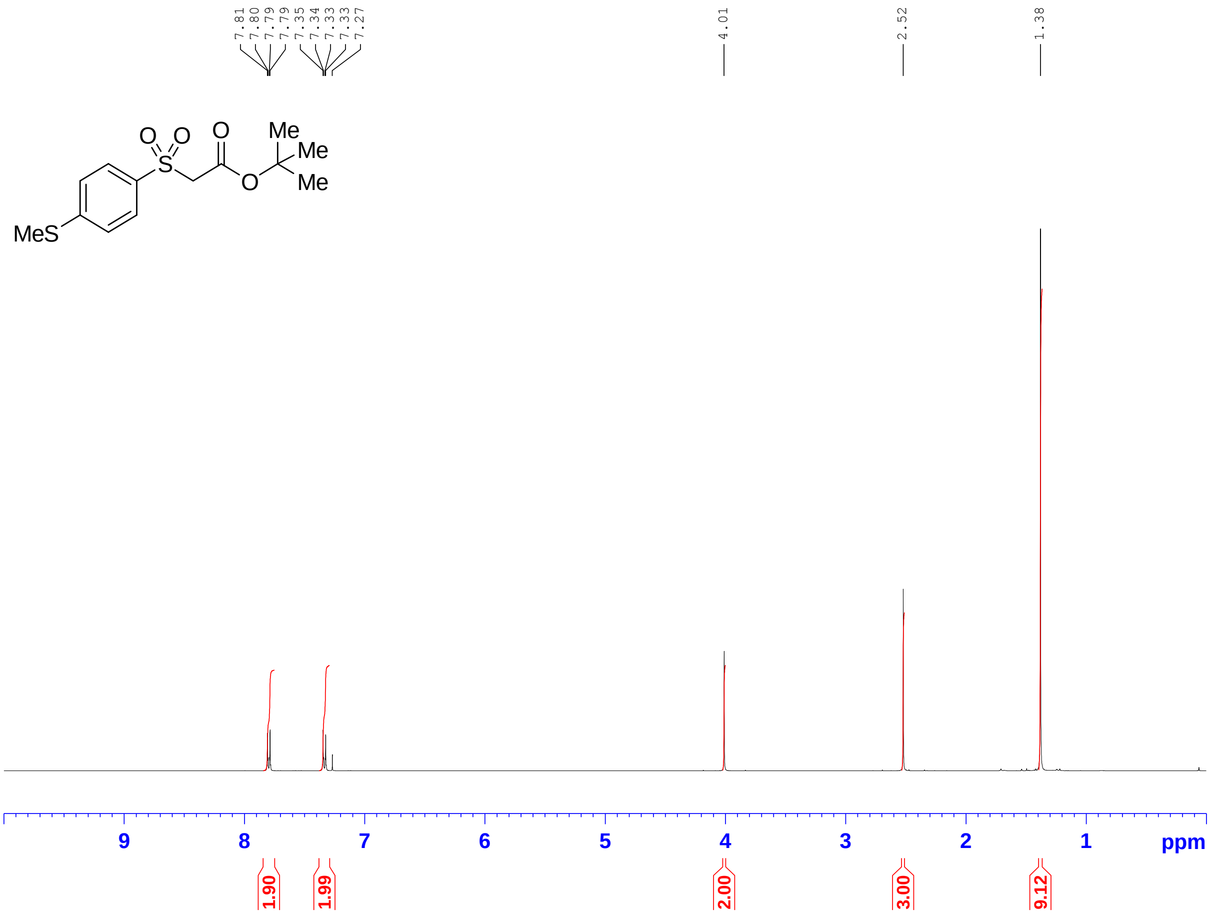
7.81
7.80
7.79
7.79
7.35
7.34
7.33
7.33
7.27
4.01
2.52
1.38
MeS
O
O
S
O
O
Me
Me
Me
9
8
7
6
5
4
3
2
1
ppm
1.90
1.99
2.00
3.00
9.12

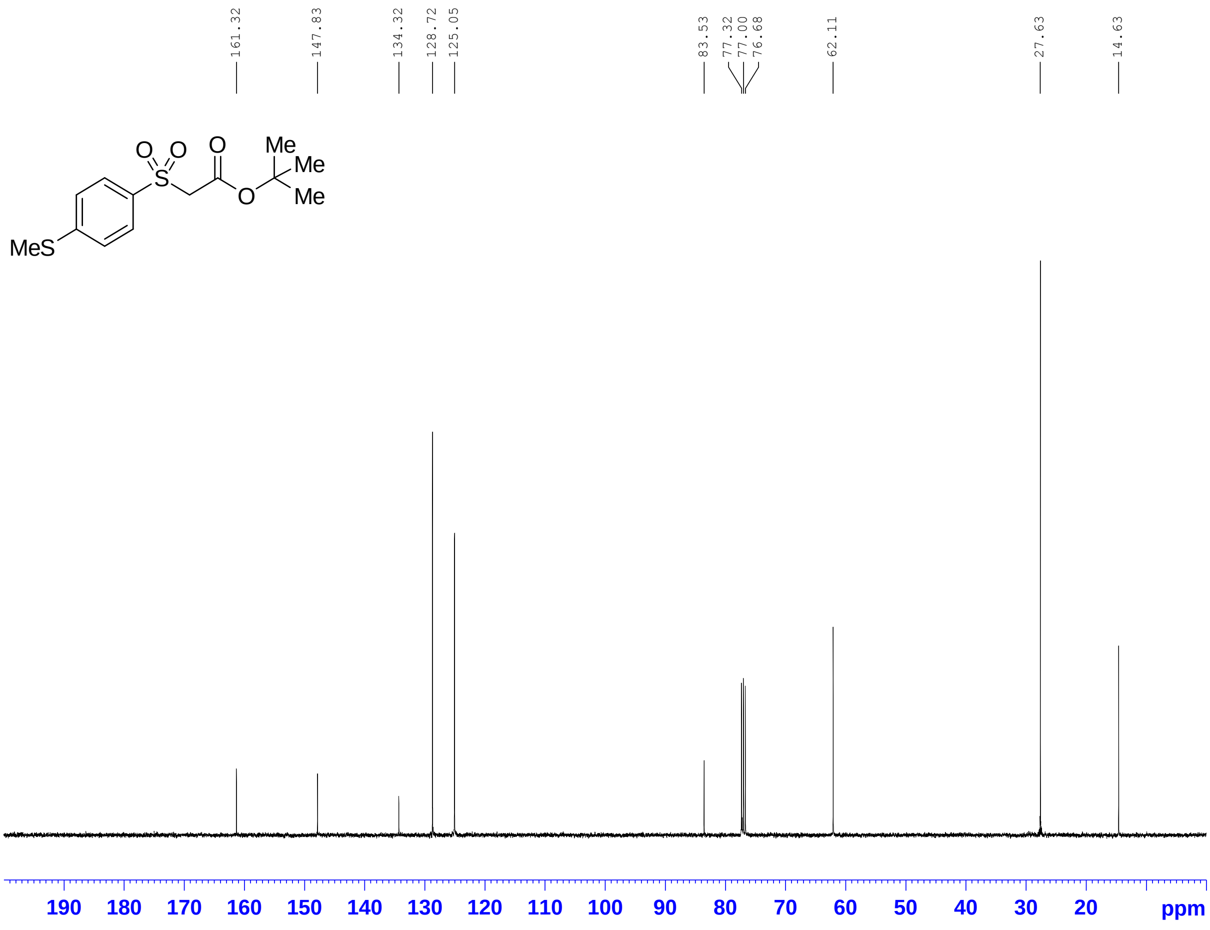
161.32
147.83
134.32
128.72
125.05
83.53
77.32
77.00
76.68
62.11
27.63
14.63
MeS
O
O
S
O
O
Me
Me
Me
190
180
170
160
150
140
130
120
110
100
90
80
70
60
50
40
30
20
ppm

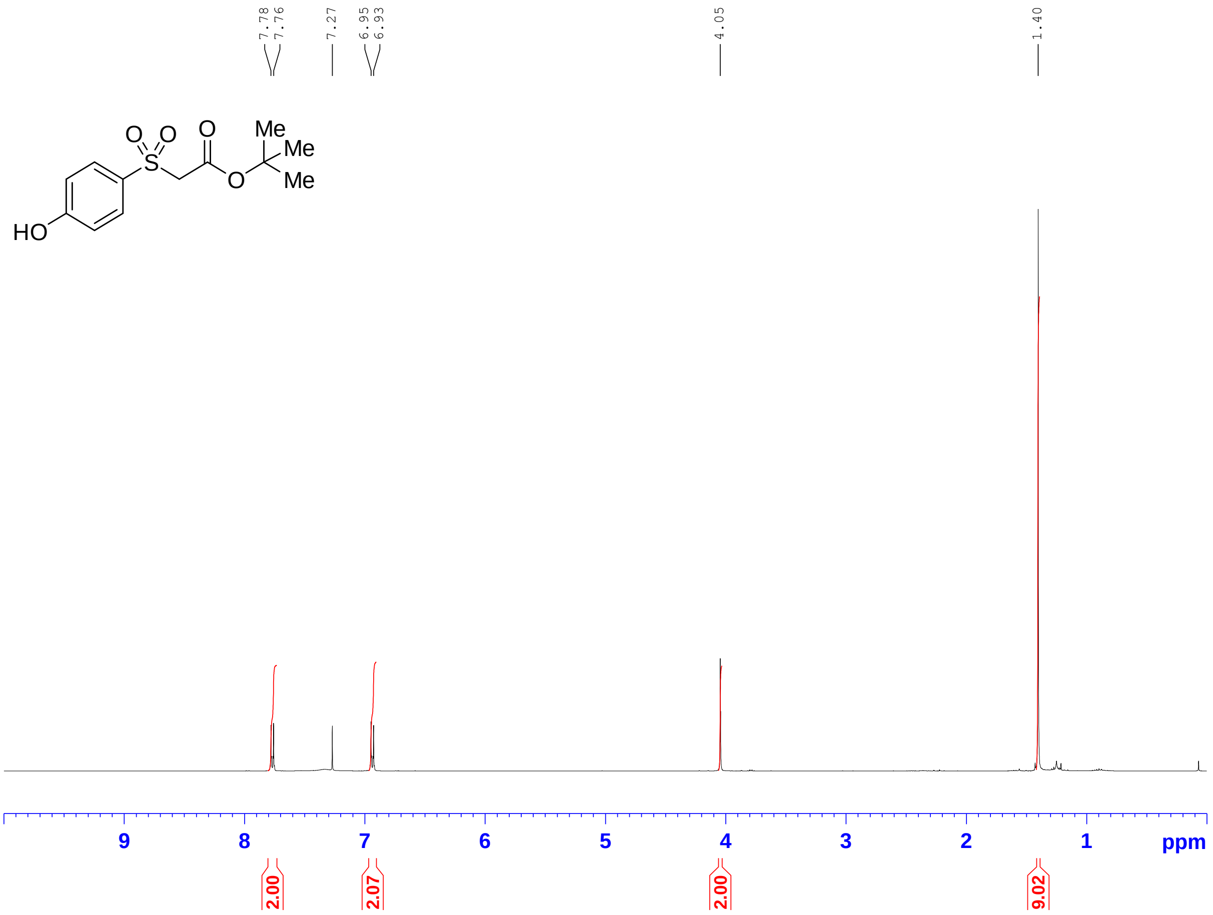
7.78
7.76
7.27
6.95
6.93
4.05
1.40
HO
O
O
S
O
O
Me
Me
Me
9
8
7
6
5
4
3
2
1
ppm
2.00
2.07
2.00
9.02

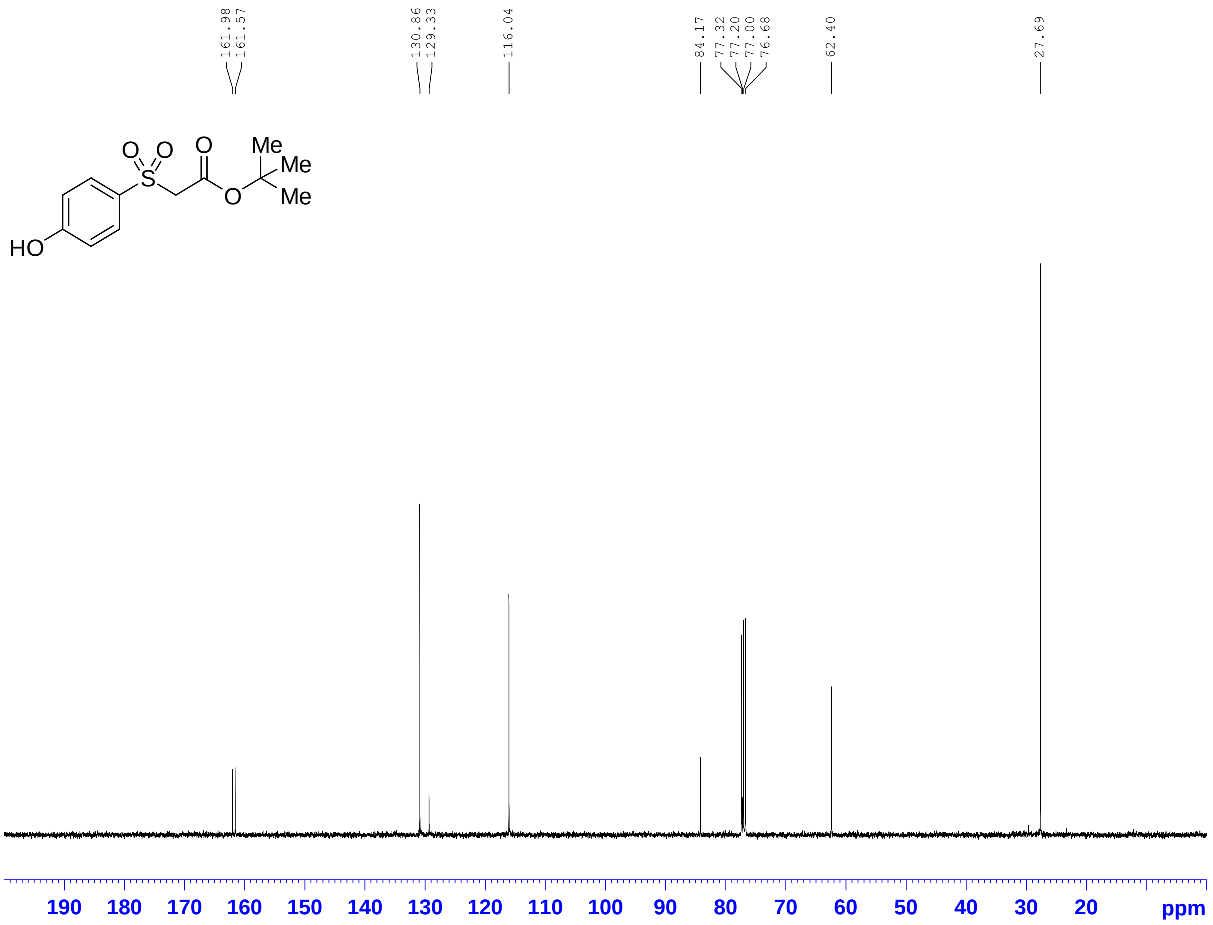
161.98
161.57
130.86
129.33
116.04
84.17
77.32
77.20
77.00
76.68
62.40
27.69
HO
O
O
S
O
O
Me
Me
Me
190
180
170
160
150
140
130
120
110
100
90
80
70
60
50
40
30
20
ppm

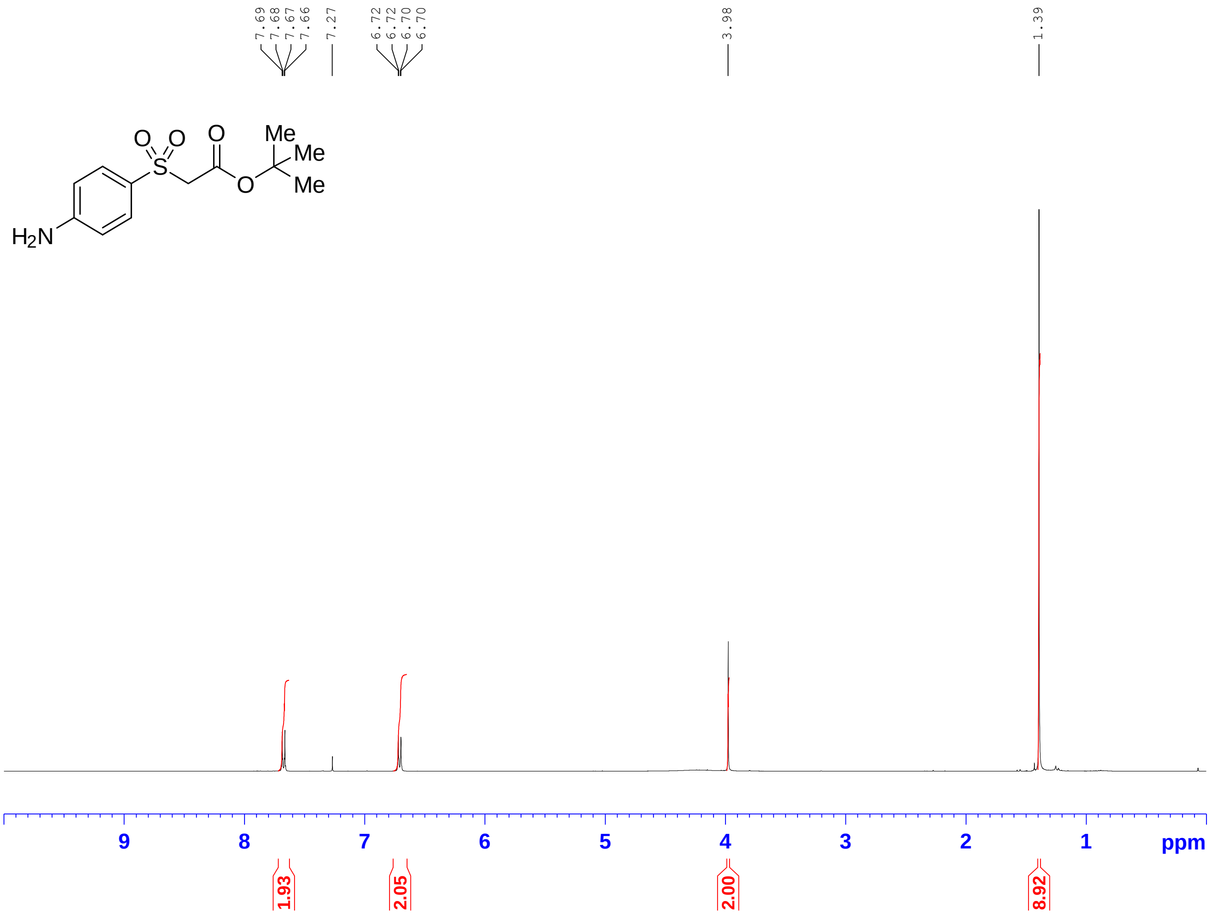
7.69
7.68
7.67
7.66
7.27
6.72
6.72
6.70
6.70
3.98
1.39
H2N
O
O
S
O
O
Me
Me
Me
9
8
7
6
5
4
3
2
1
ppm
1.93
2.05
2.00
8.92

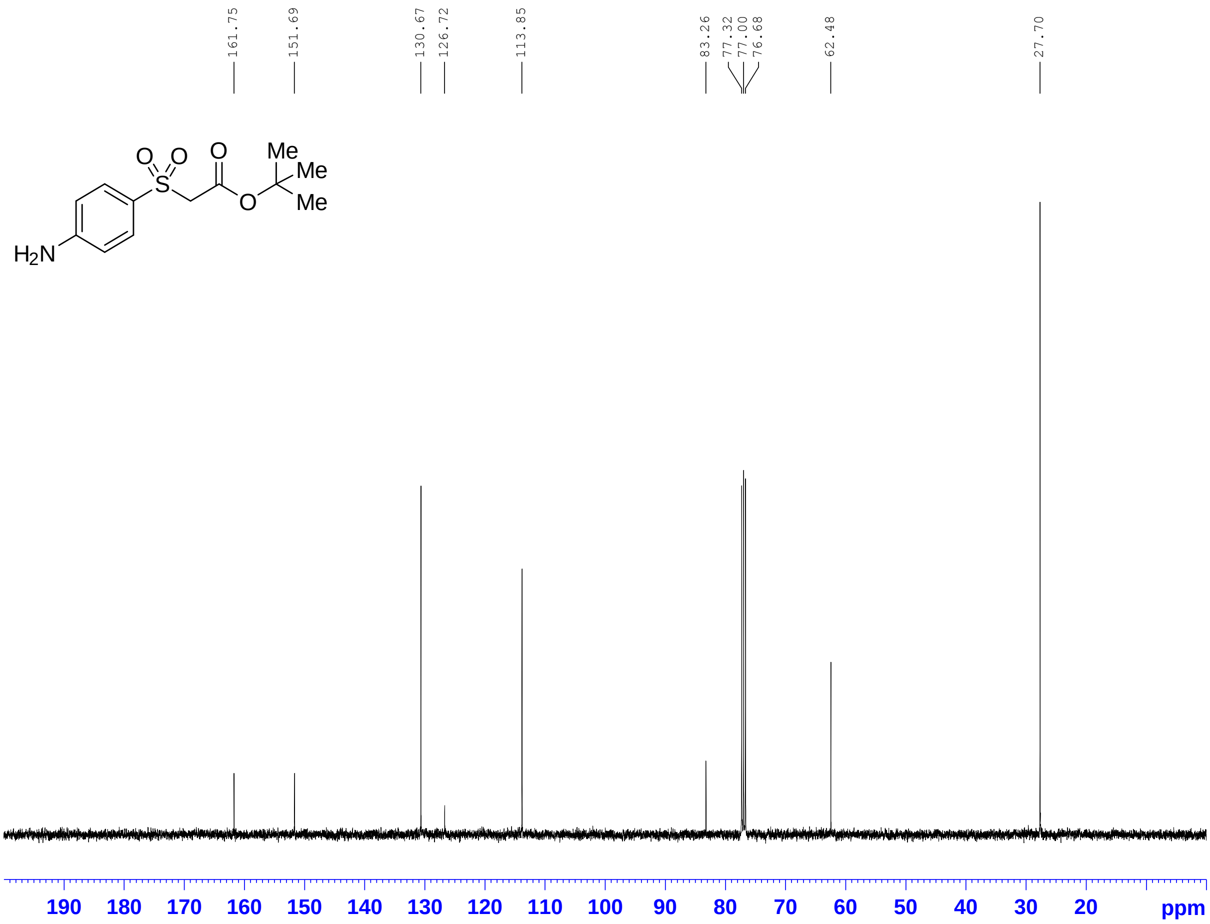
161.75
151.69
130.67
126.72
113.85
83.26
77.32
77.00
76.68
62.48
27.70
H2N
O
O
S
O
O
Me
Me
Me
190
180
170
160
150
140
130
120
110
100
90
80
70
60
50
40
30
20
ppm

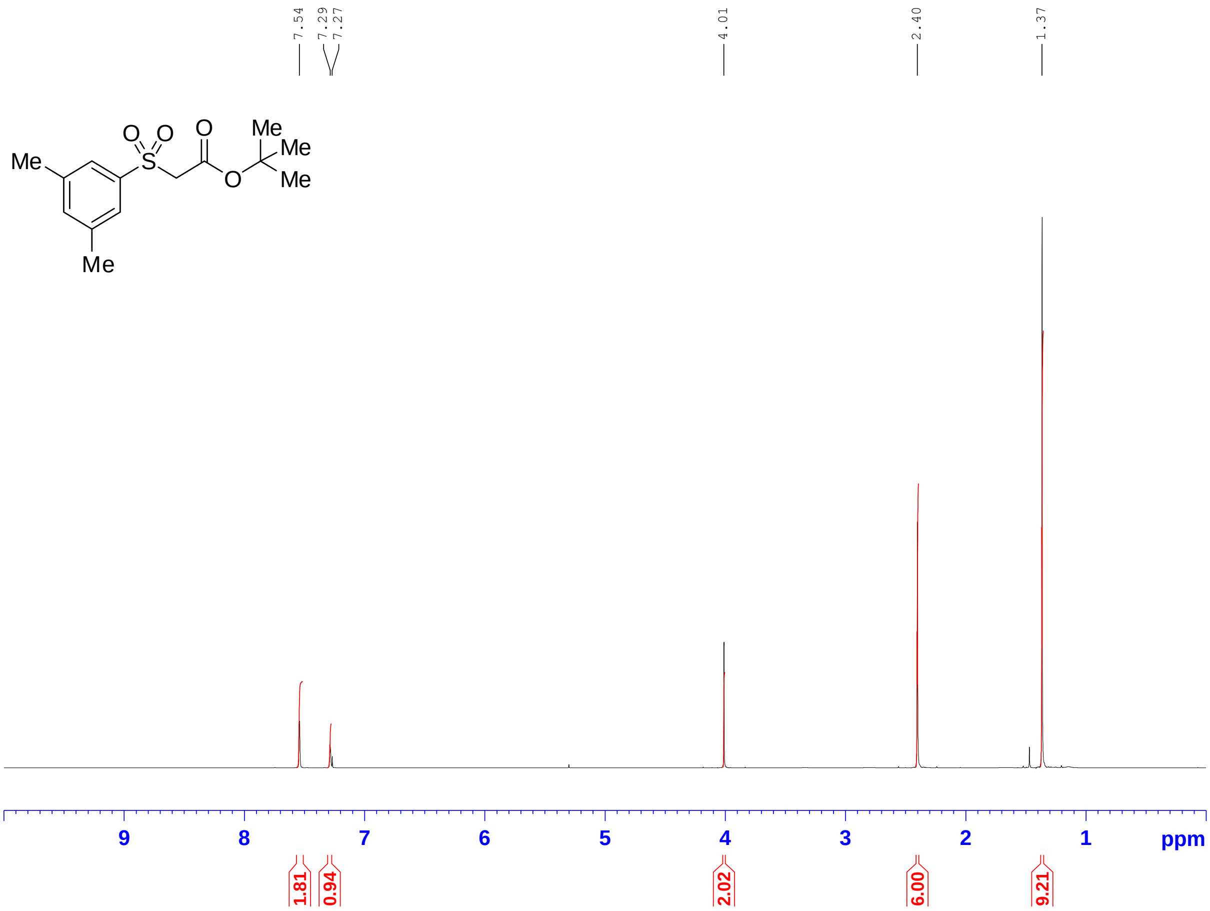
7.54
7.29
7.27
4.01
2.40
1.37
Me
O
O
S
O
O
Me
Me
Me
Me
9
8
7
6
5
4
3
2
1
ppm
1.81
0.94
2.02
6.00
9.21

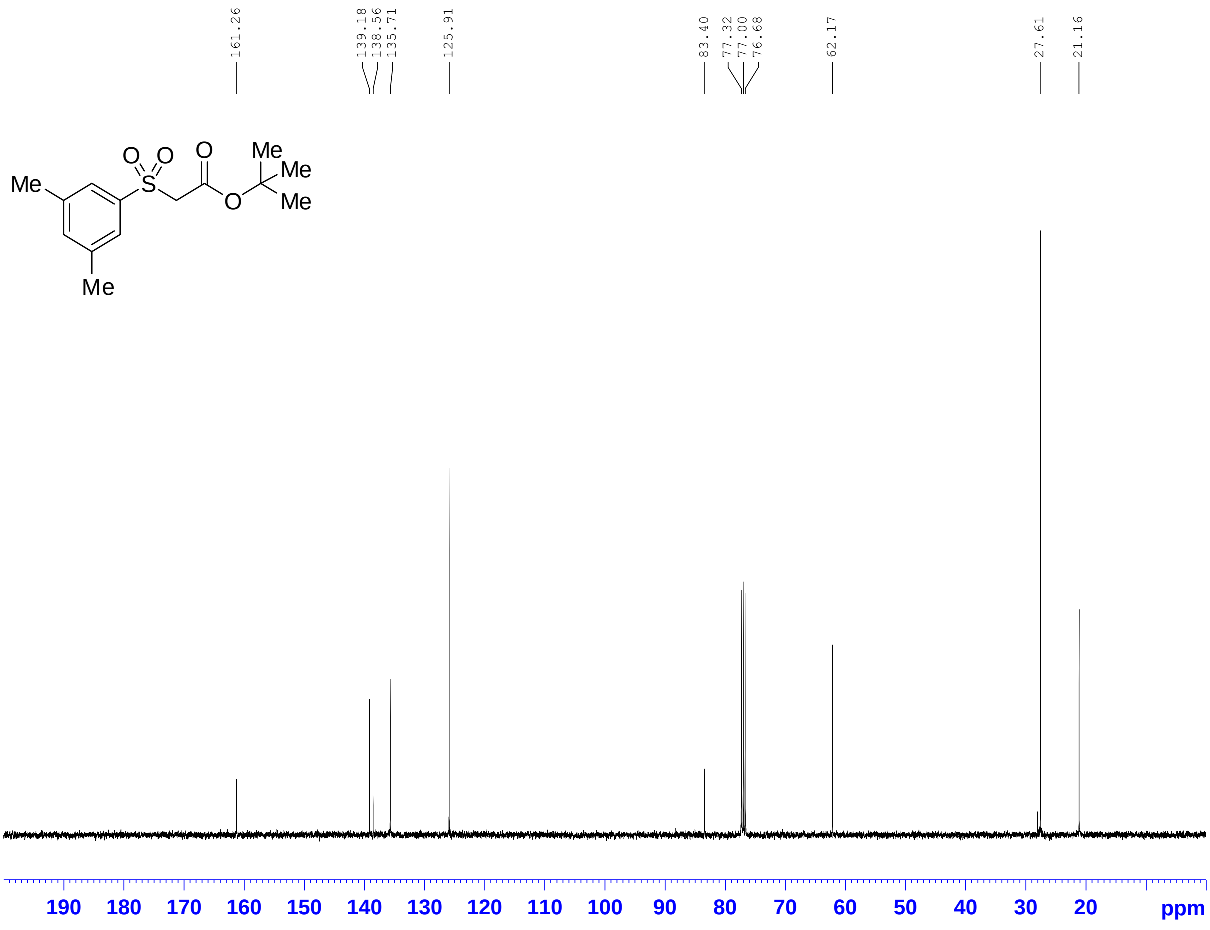
161.26
139.18
138.56
135.71
125.91
83.40
77.32
77.00
76.68
62.17
27.61
21.16
Me
O
O
S
O
O
Me
Me
Me
Me
190
180
170
160
150
140
130
120
110
100
90
80
70
60
50
40
30
20
ppm

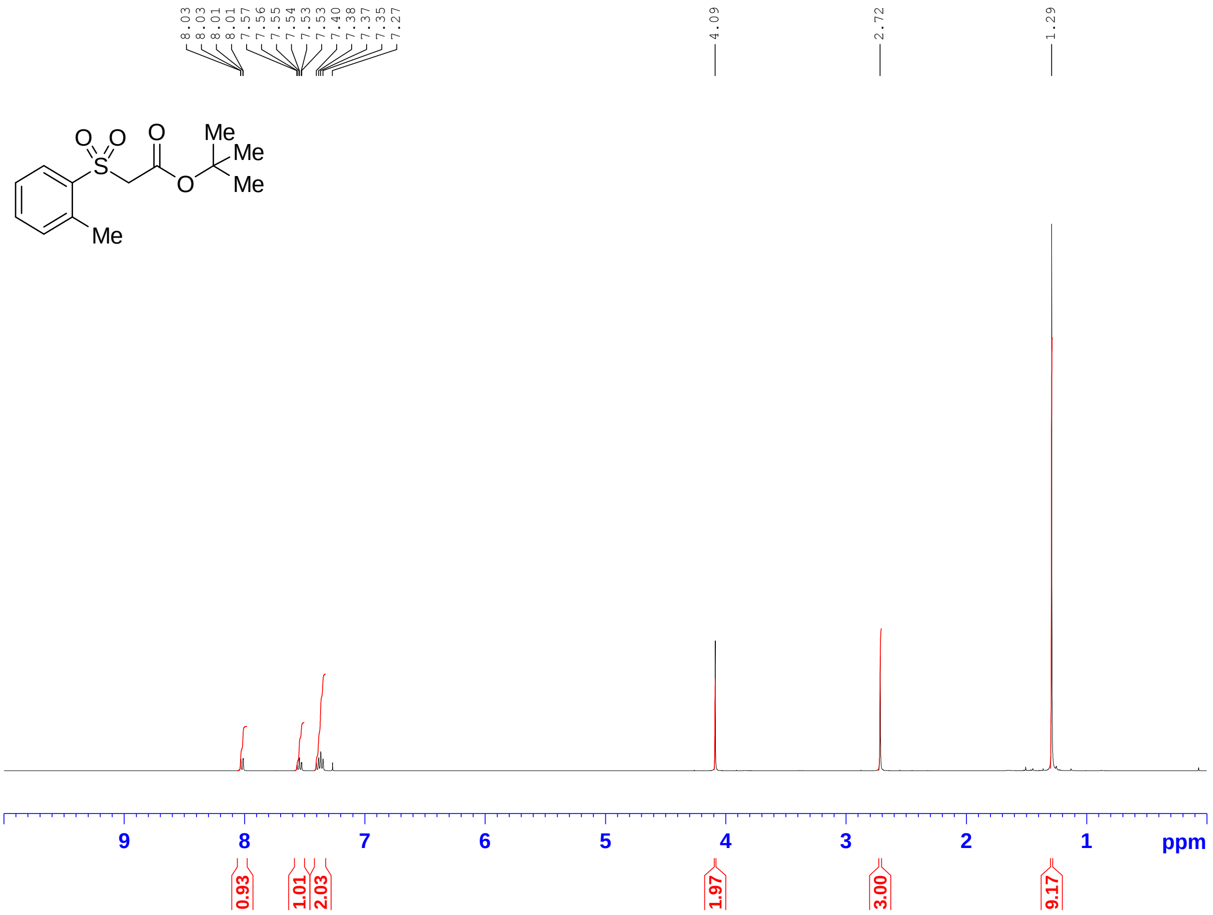
8.03
8.03
8.01
8.01
7.57
7.56
7.55
7.54
7.53
7.53
7.40
7.38
7.37
7.35
7.27
4.09
2.72
1.29
O
O
S
O
O
Me
Me
Me
Me
9
8
7
6
5
4
3
2
1
ppm
0.93
1.01
2.03
1.97
3.00
9.17

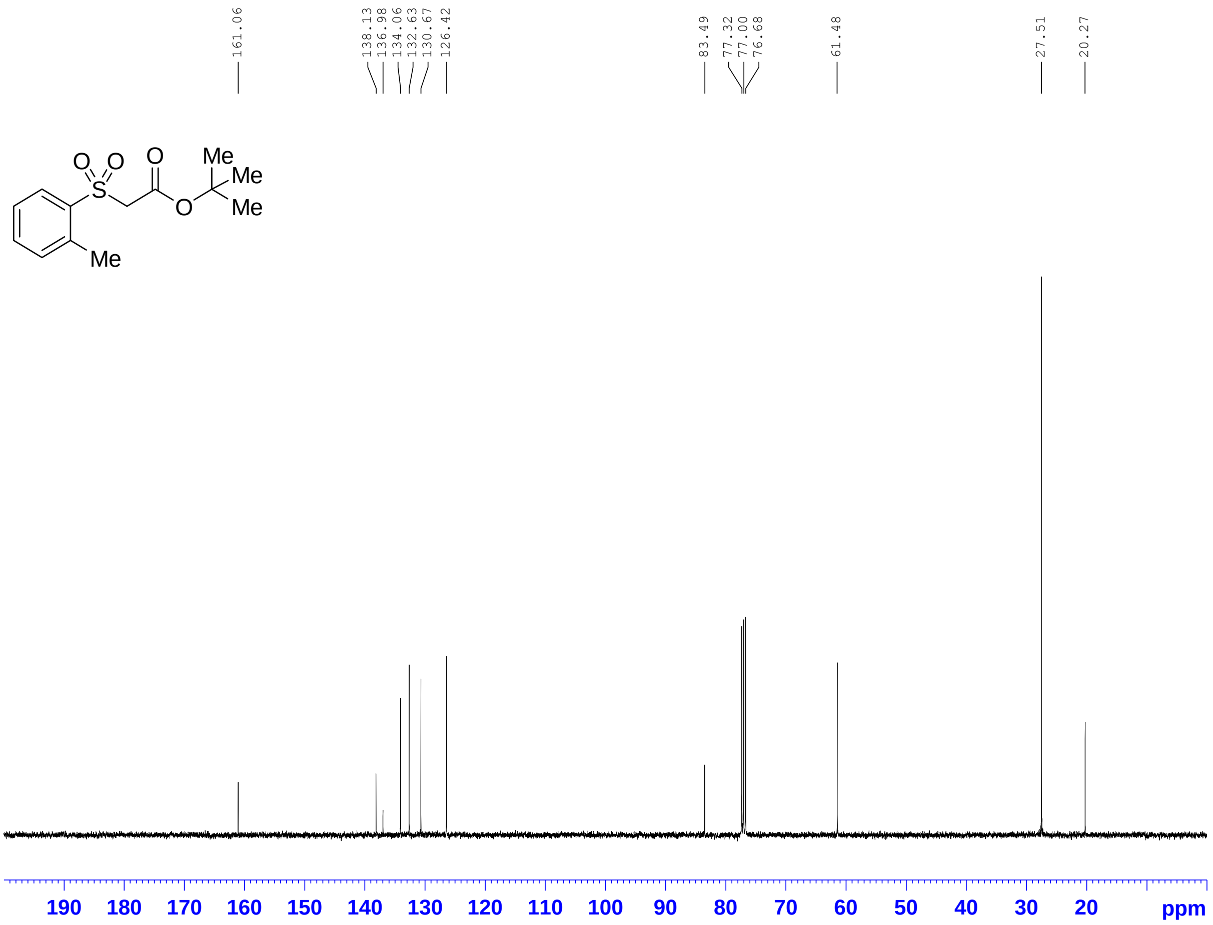
161.06
138.13
136.98
134.06
132.63
130.67
126.42
83.49
77.32
77.00
76.68
61.48
27.51
20.27
O
O
S
O
O
Me
Me
Me
Me
190
180
170
160
150
140
130
120
110
100
90
80
70
60
50
40
30
20
ppm

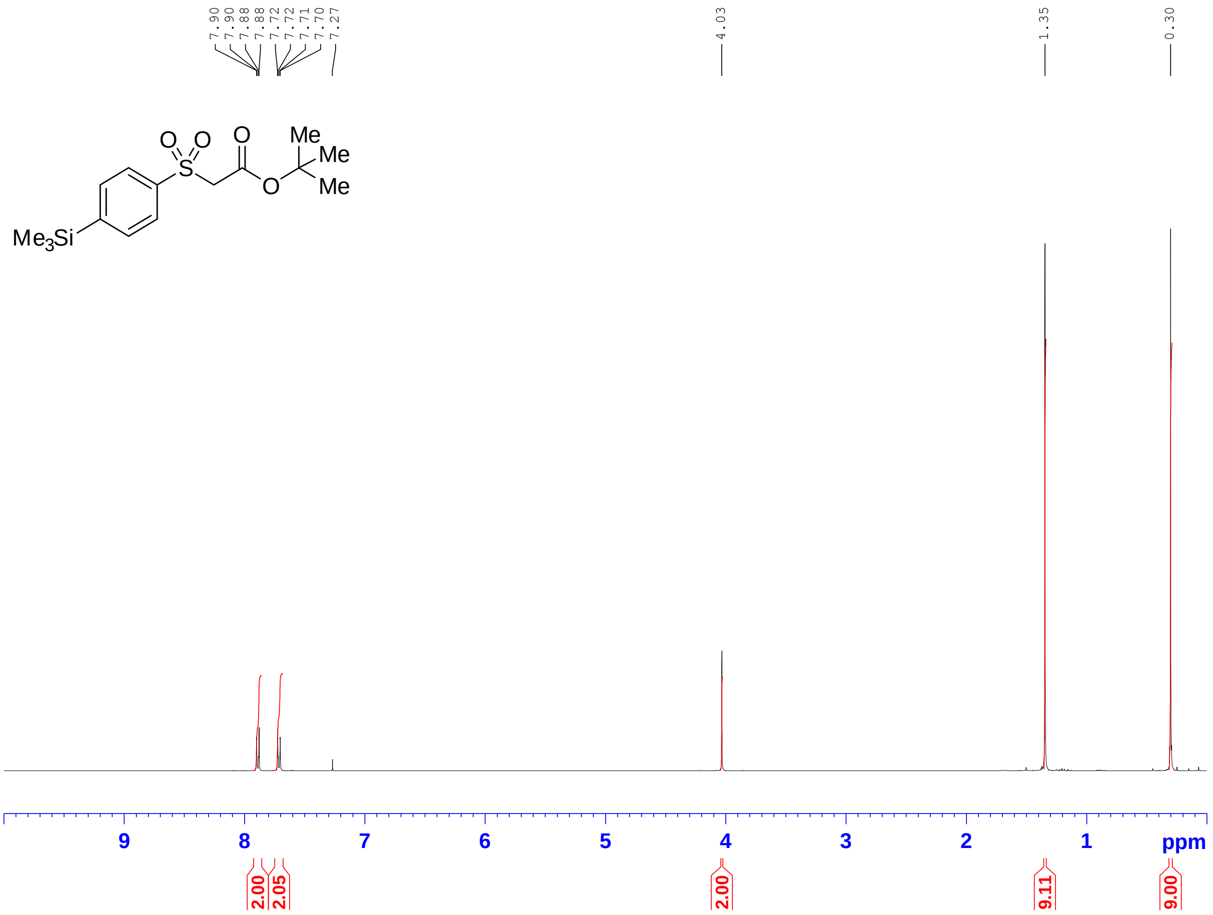
7.90
7.90
7.88
7.88
7.72
7.72
7.71
7.70
7.27
4.03
1.35
0.30
Me3Si
Me
Me
Me
ppm
2.00
2.05
2.00
9.11
9.00

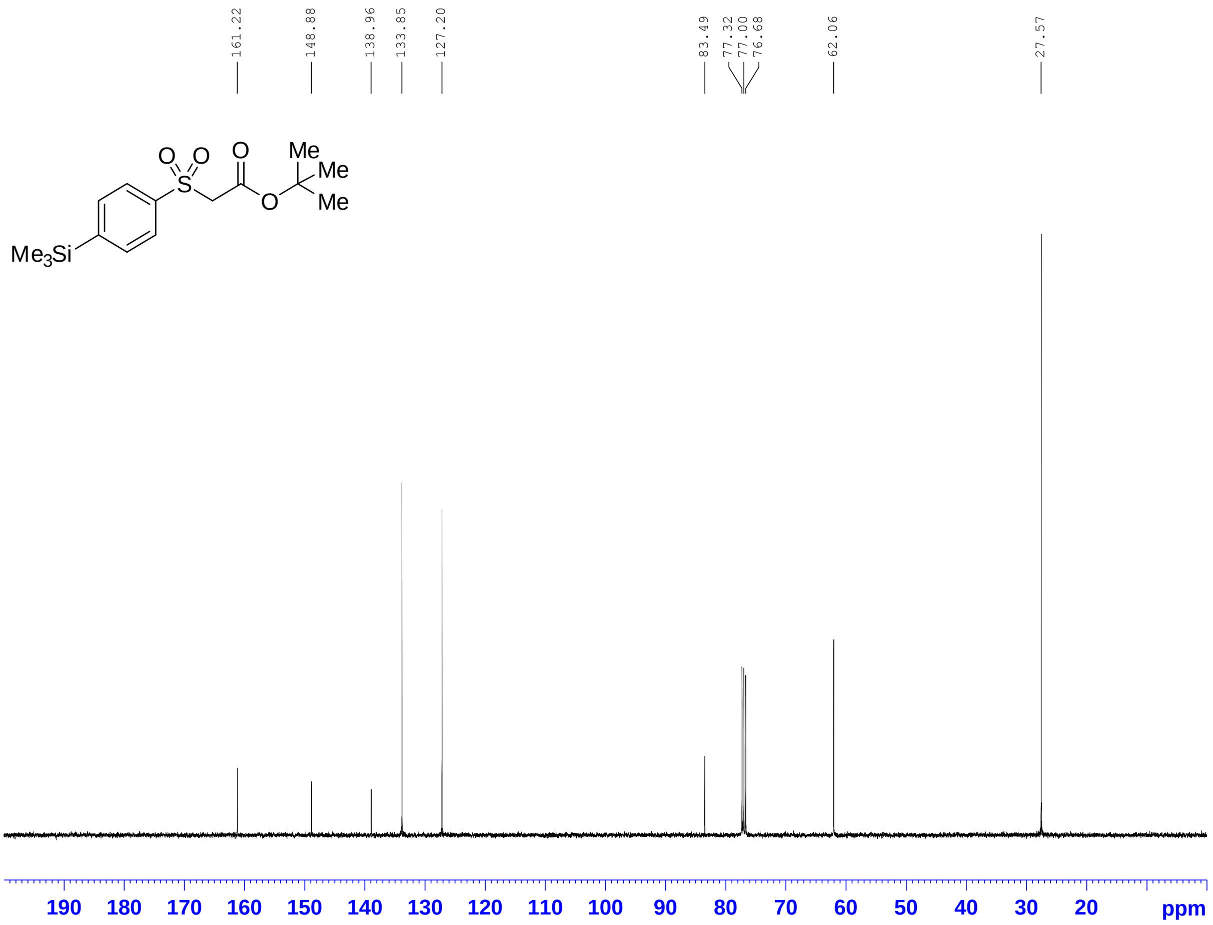
161.22
148.88
138.96
133.85
127.20
83.49
77.32
77.00
76.68
62.06
27.57
Me3Si
Me
Me
Me
ppm

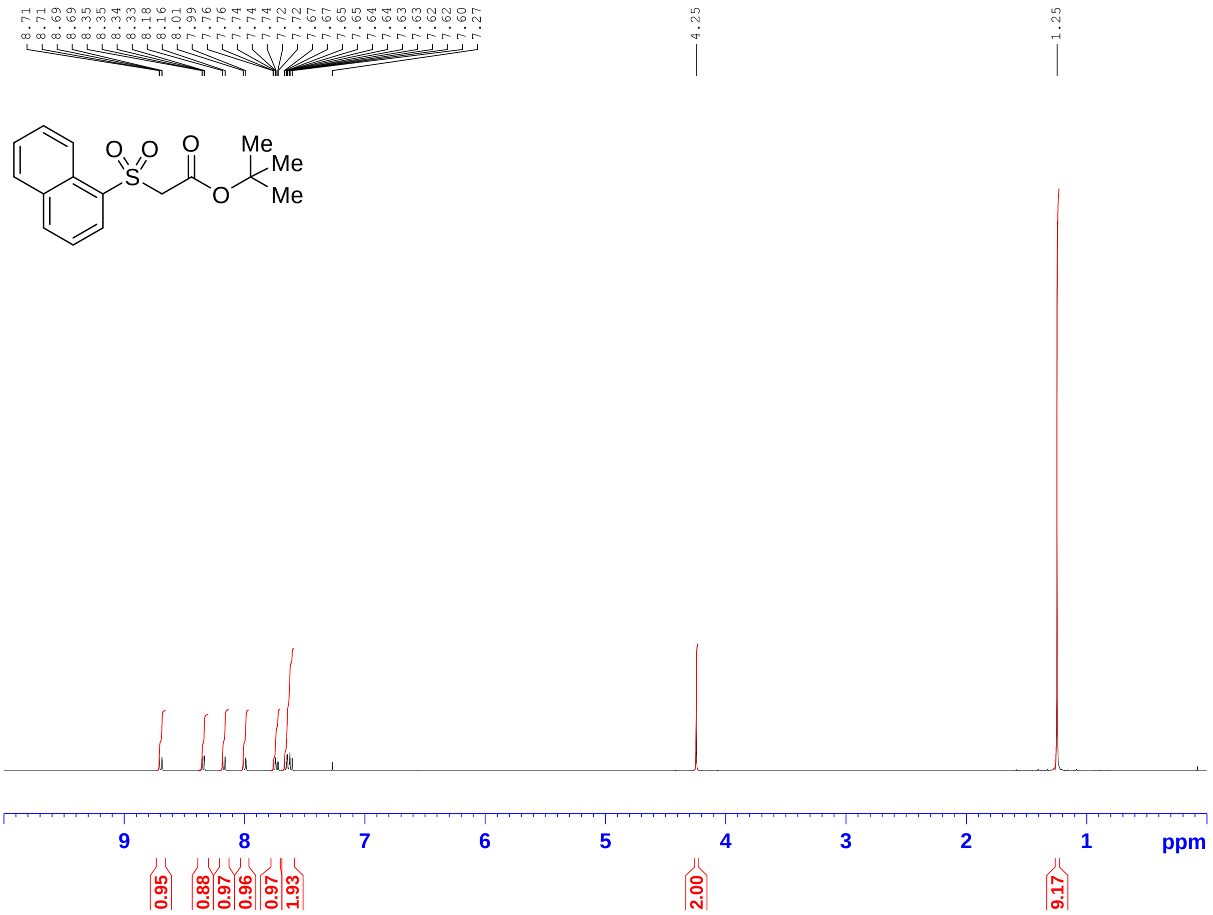
8.71
8.71
8.69
8.69
8.35
8.35
8.34
8.33
8.18
8.16
8.01
7.99
7.76
7.76
7.74
7.74
7.74
7.72
7.72
7.67
7.67
7.65
7.65
7.64
7.64
7.63
7.63
7.62
7.62
7.60
7.27
4.25
1.25
Me
Me
Me
O
O
S
O
O
9
8
7
6
5
4
3
2
1
ppm
0.95
0.88
0.97
0.96
0.97
1.93
2.00
9.17

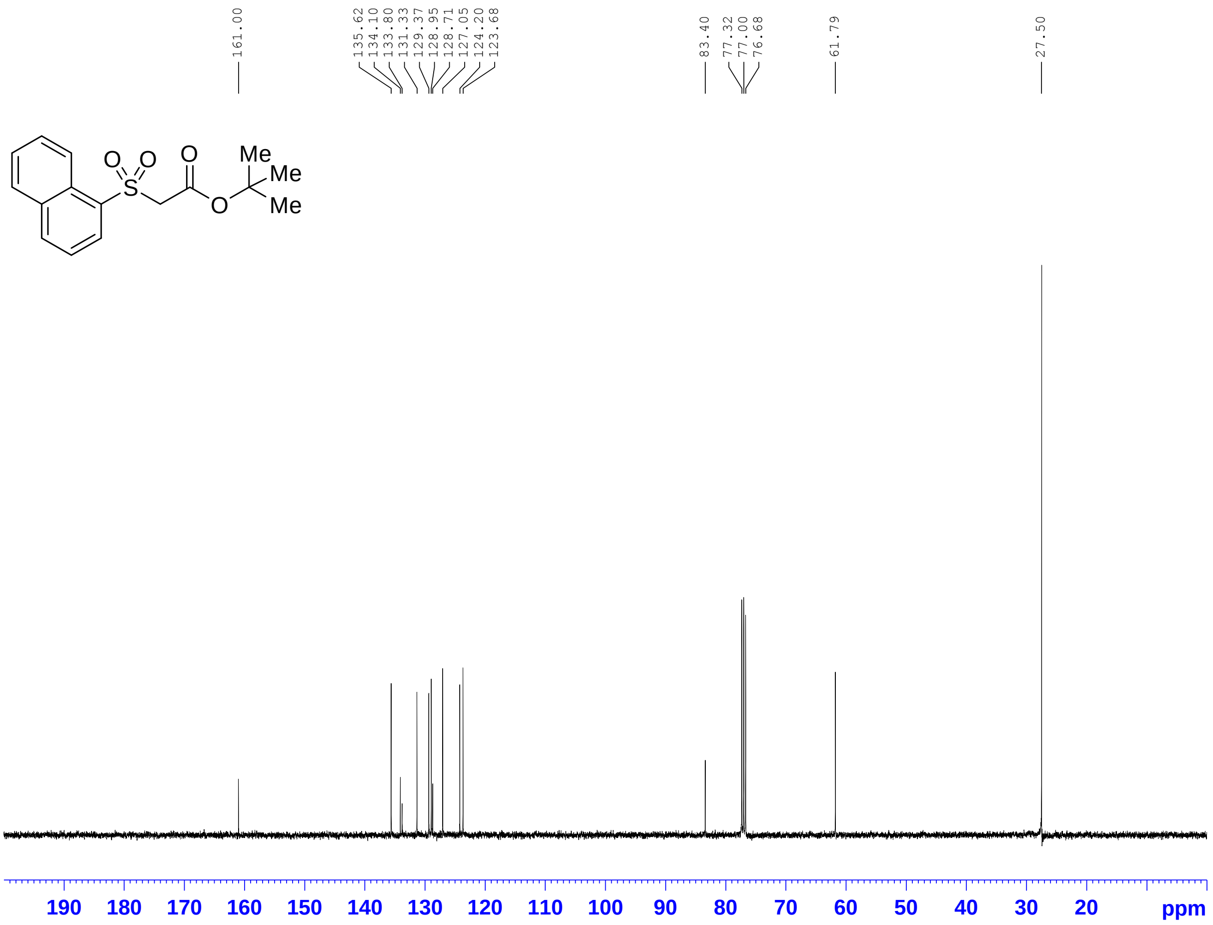
161.00
135.62
134.10
133.80
131.33
129.37
128.95
128.71
127.05
124.20
123.68
83.40
77.32
77.00
76.68
61.79
27.50
190
180
170
160
150
140
130
120
110
100
90
80
70
60
50
40
30
20
ppm

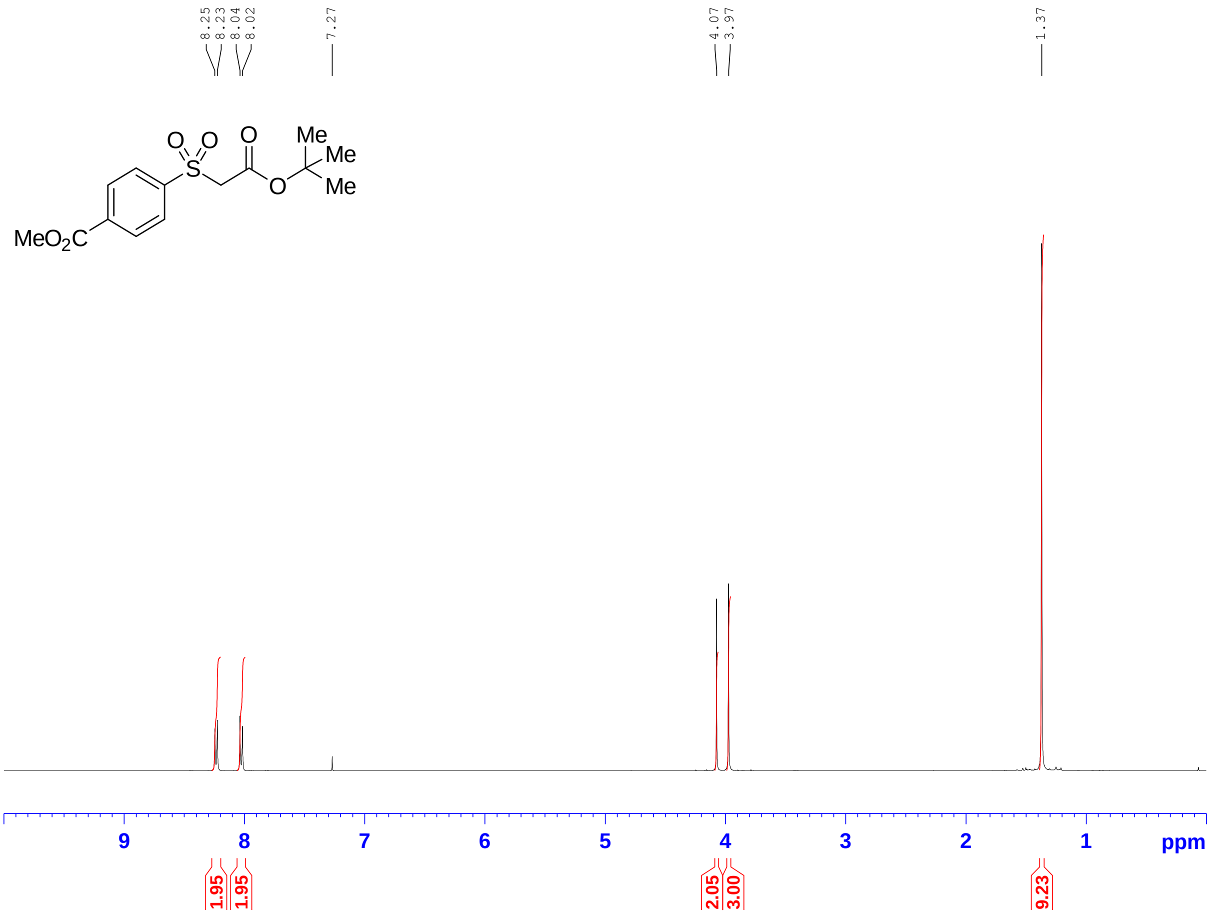
8.25
8.23
8.04
8.02
7.27
4.07
3.97
1.37
MeO2C
O
O
S
O
O
Me
Me
Me
9
8
7
6
5
4
3
2
1
ppm
1.95
1.95
2.05
3.00
9.23

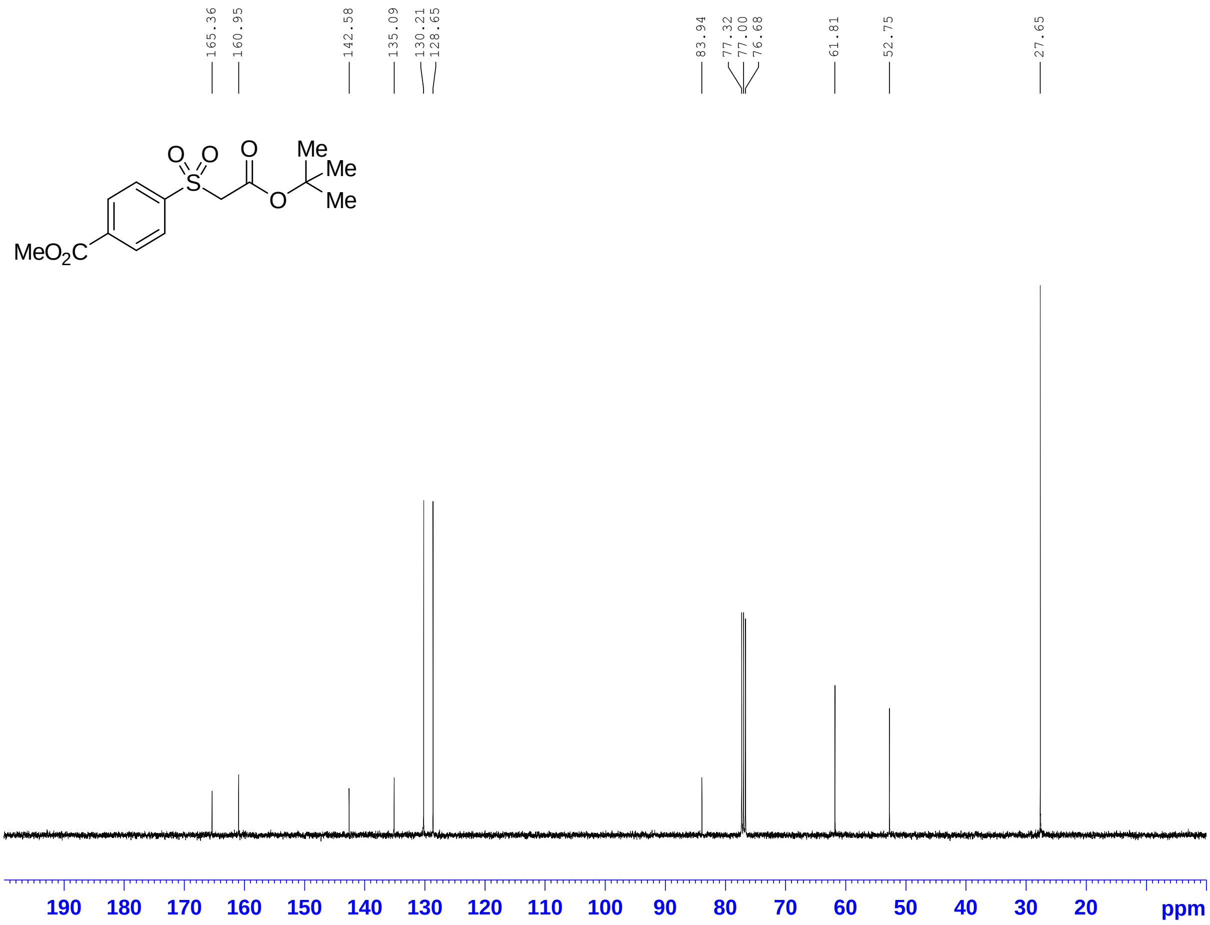
165.36
160.95
142.58
135.09
130.21
128.65
83.94
77.32
77.00
76.68
61.81
52.75
27.65
MeO2C
O
O
S
O
O
Me
Me
Me
190
180
170
160
150
140
130
120
110
100
90
80
70
60
50
40
30
20
ppm

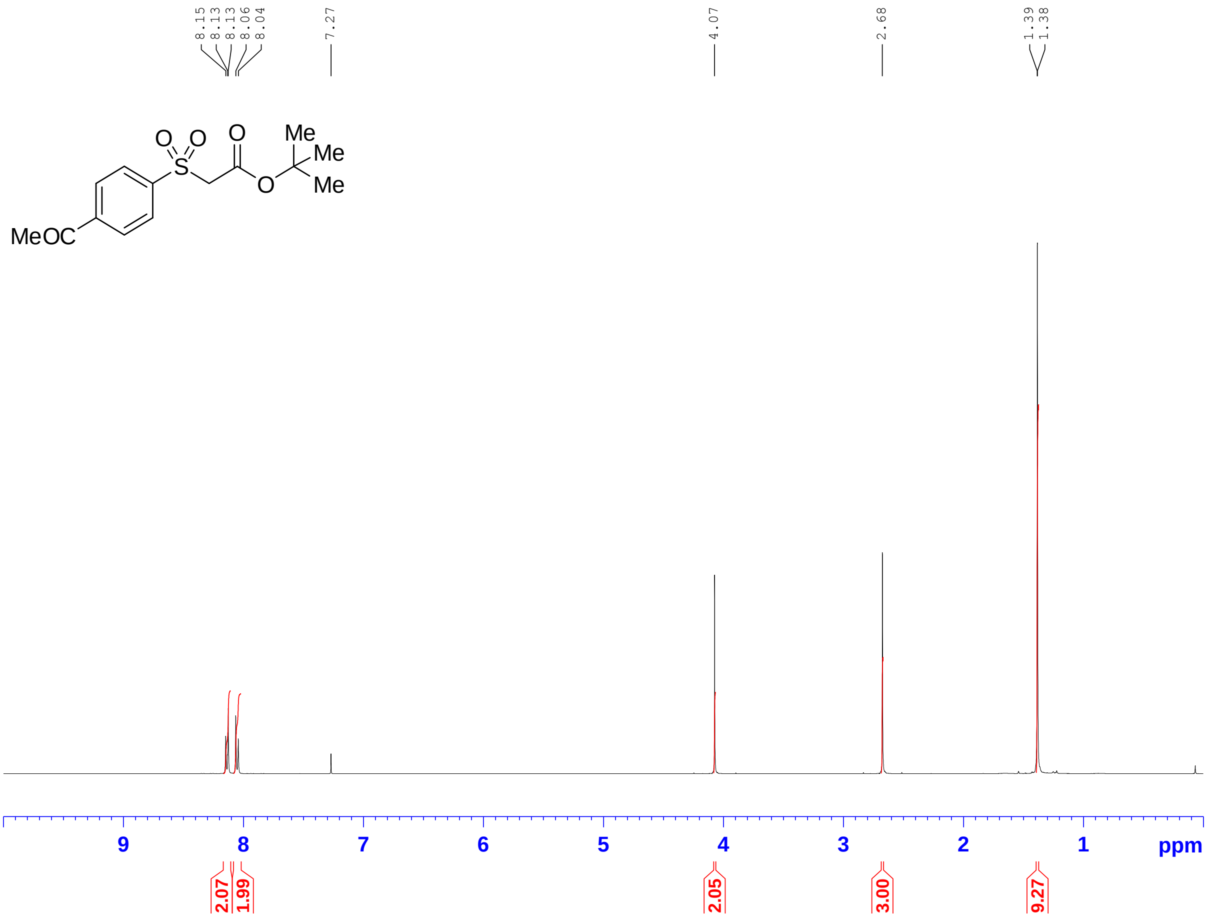
8.15
8.13
8.13
8.06
8.04
7.27
4.07
2.68
1.39
1.38
MeOC
O
O
S
O
O
Me
Me
Me
9
8
7
6
5
4
3
2
1
ppm
2.07
1.99
2.05
3.00
9.27

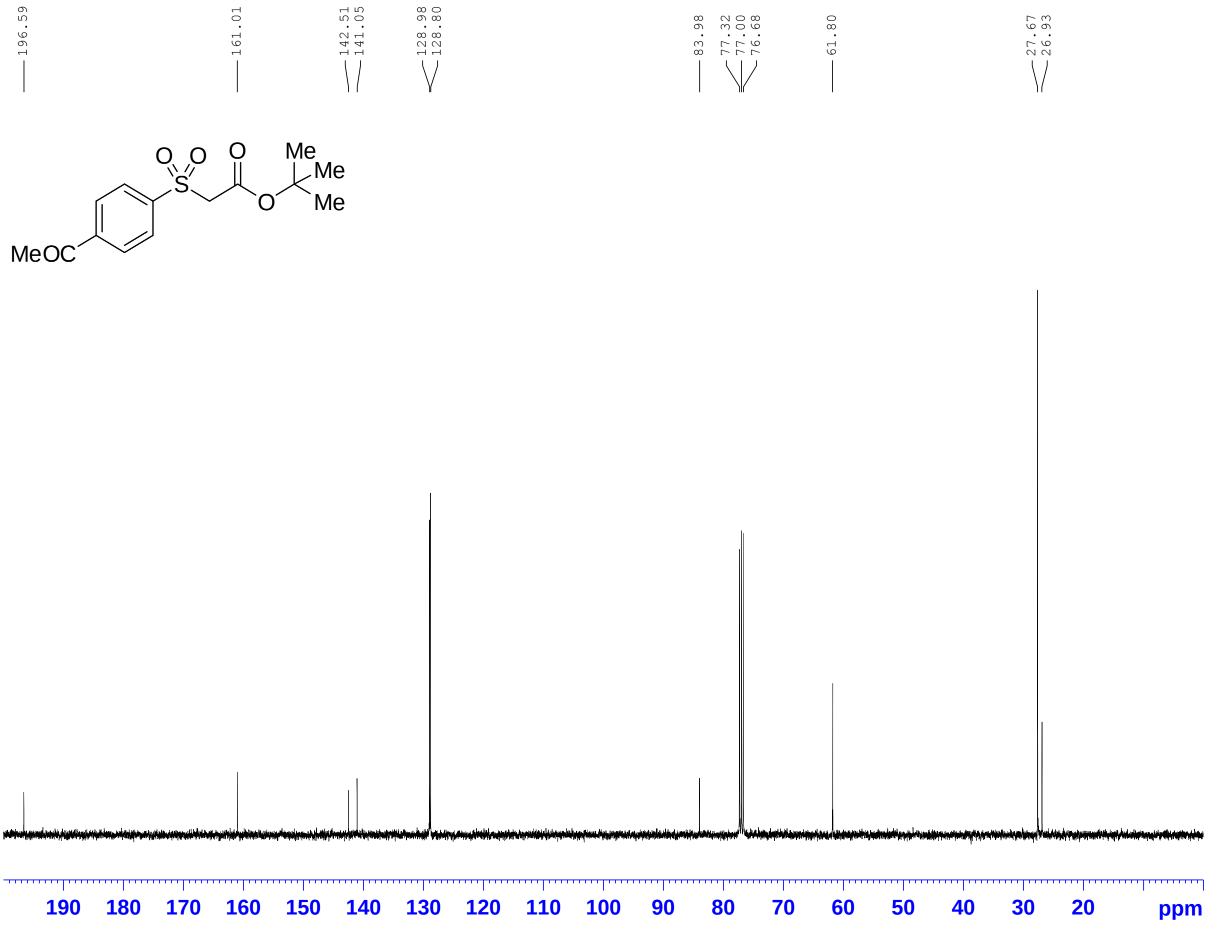
196.59
161.01
142.51
141.05
128.98
128.80
83.98
77.32
77.00
76.68
61.80
27.67
26.93
MeOC
O
O
S
O
O
Me
Me
Me
190
180
170
160
150
140
130
120
110
100
90
80
70
60
50
40
30
20
ppm

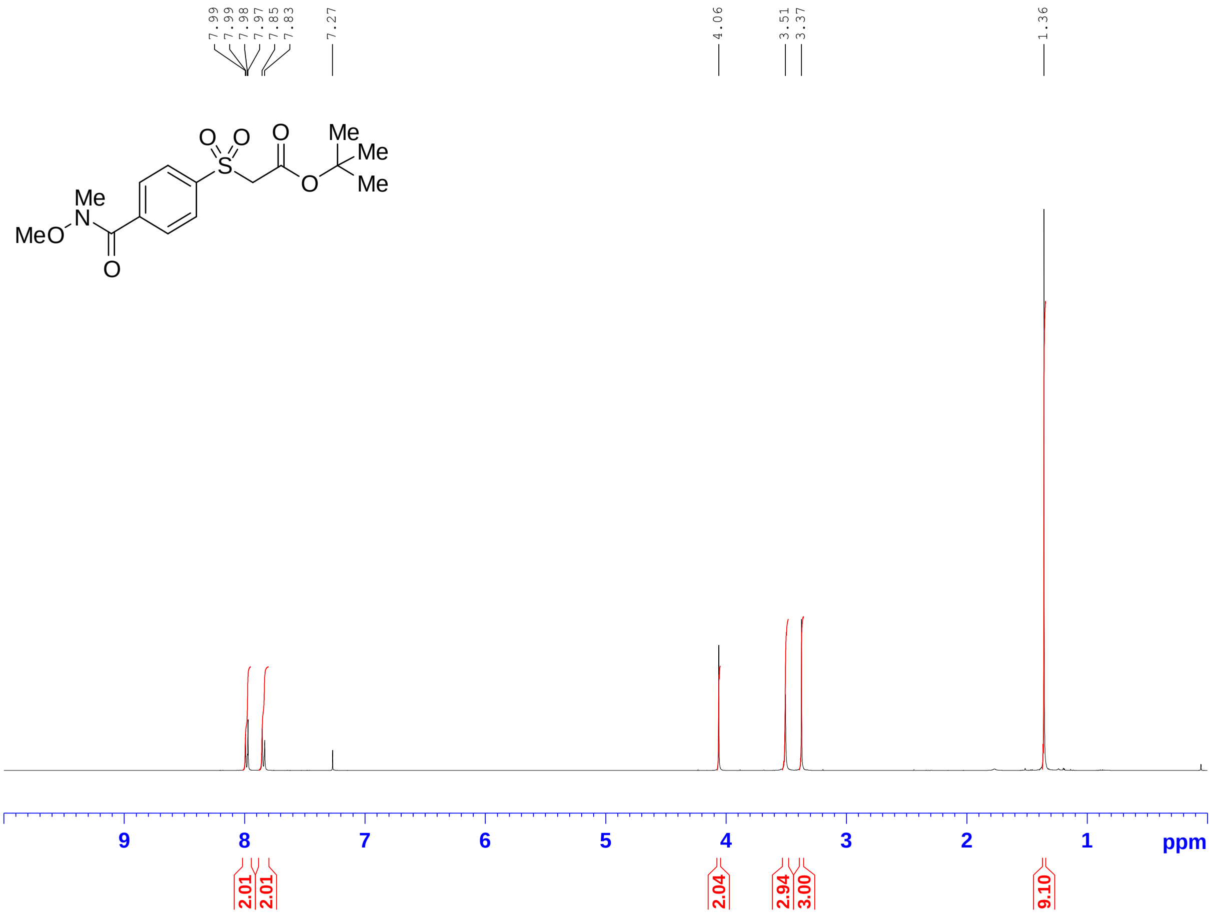
7.99
7.99
7.98
7.97
7.85
7.83
7.27
4.06
3.51
3.37
1.36
MeO
N
Me
O
S
O O
O
O
Me
Me
Me
9
8
7
6
5
4
3
2
1
ppm
2.01
2.01
2.04
2.94
3.00
9.10

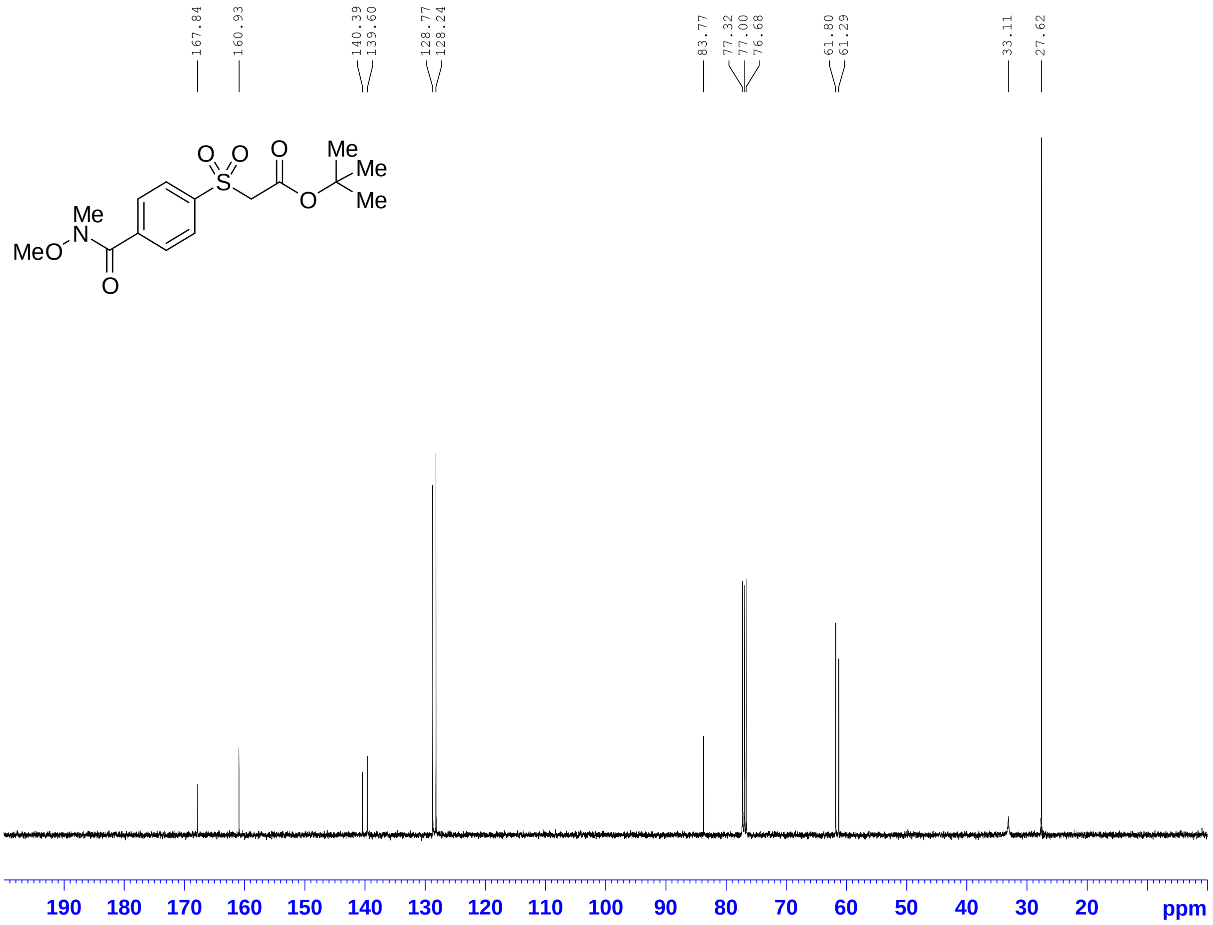
167.84
160.93
140.39
139.60
128.77
128.24
83.77
77.32
77.00
76.68
61.80
61.29
33.11
27.62
MeO
N
Me
O
S
O O
O
O
Me
Me
Me
190
180
170
160
150
140
130
120
110
100
90
80
70
60
50
40
30
20
ppm

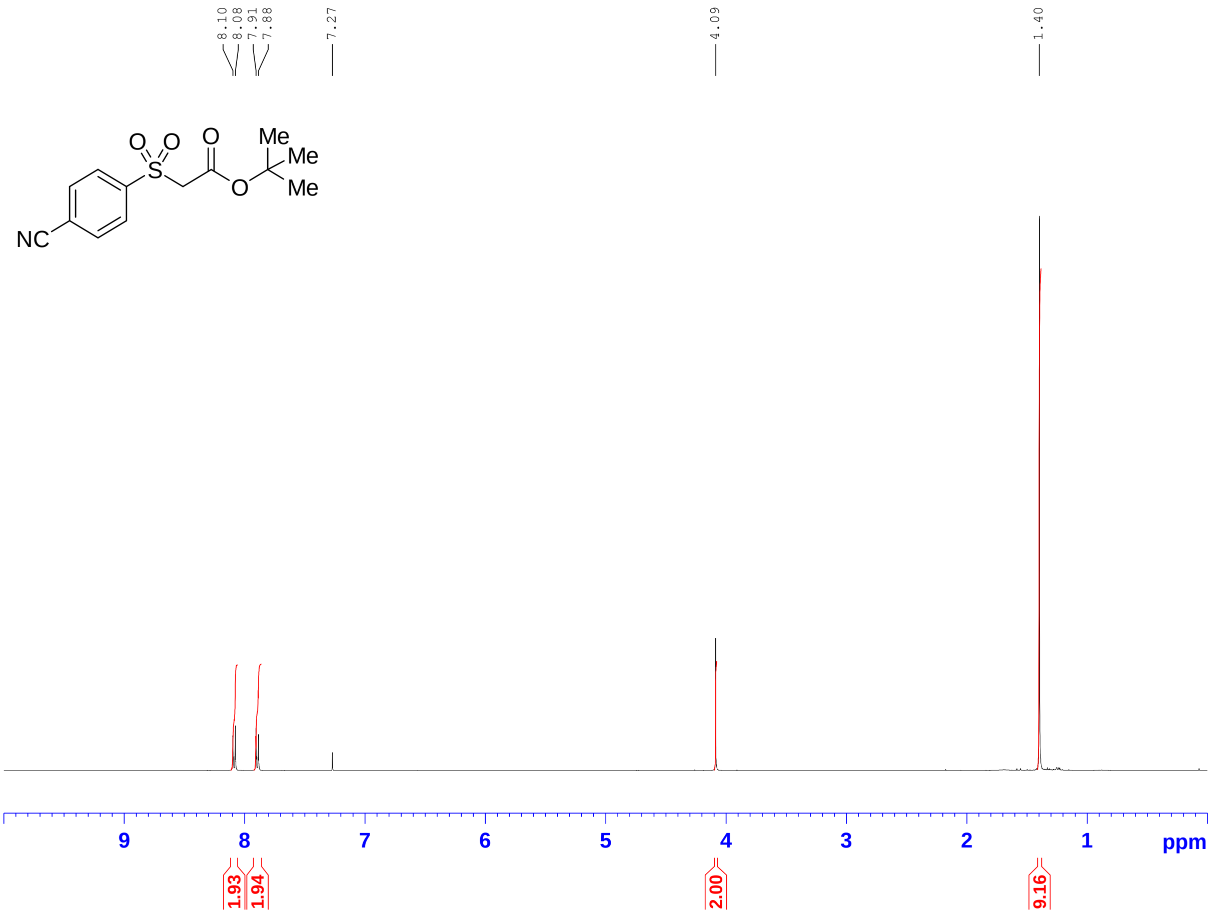
8.10
8.08
7.91
7.88
7.27
4.09
1.40
NC
O
O
S
O
O
Me
Me
Me
9
8
7
6
5
4
3
2
1
ppm
1.93
1.94
2.00
9.16

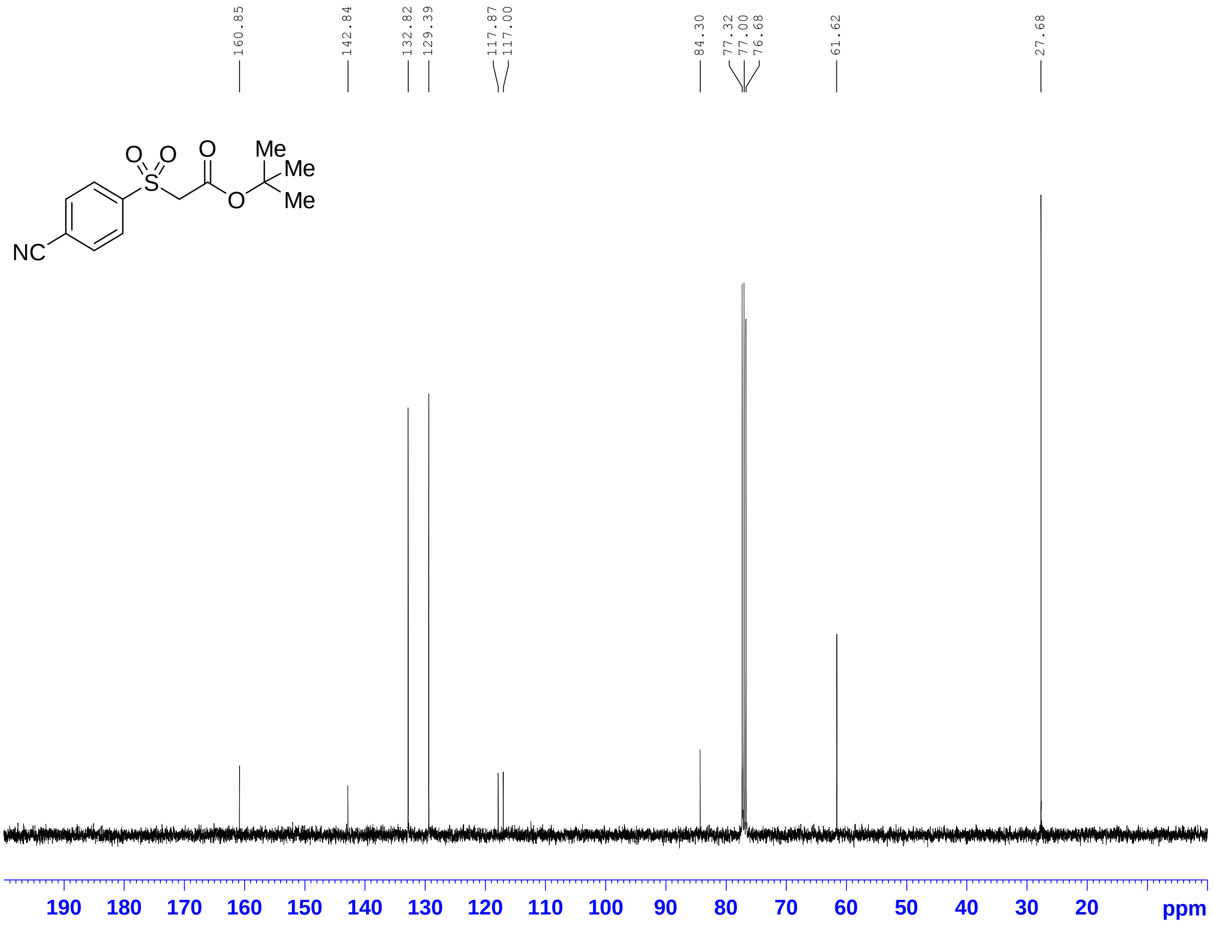
160.85
142.84
132.82
129.39
117.87
117.00
84.30
77.32
77.00
76.68
61.62
27.68
NC
O
O
S
O
O
Me
Me
Me
190
180
170
160
150
140
130
120
110
100
90
80
70
60
50
40
30
20
ppm

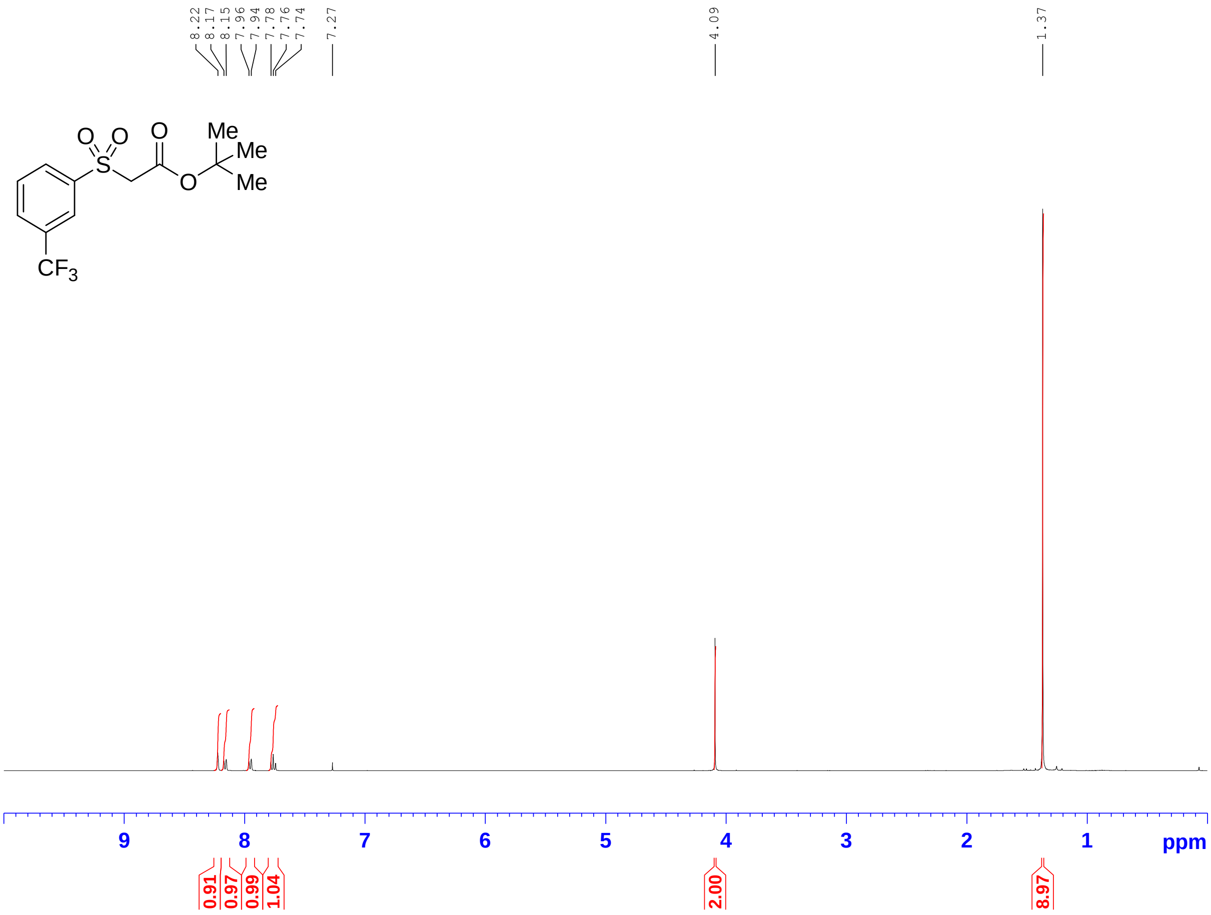
8.22
8.17
8.15
7.96
7.94
7.78
7.76
7.74
7.27
4.09
1.37
Me
Me
Me
O
O
O
S
O
CF3
9
8
7
6
5
4
3
2
1
ppm
0.91
0.97
0.99
1.04
2.00
8.97

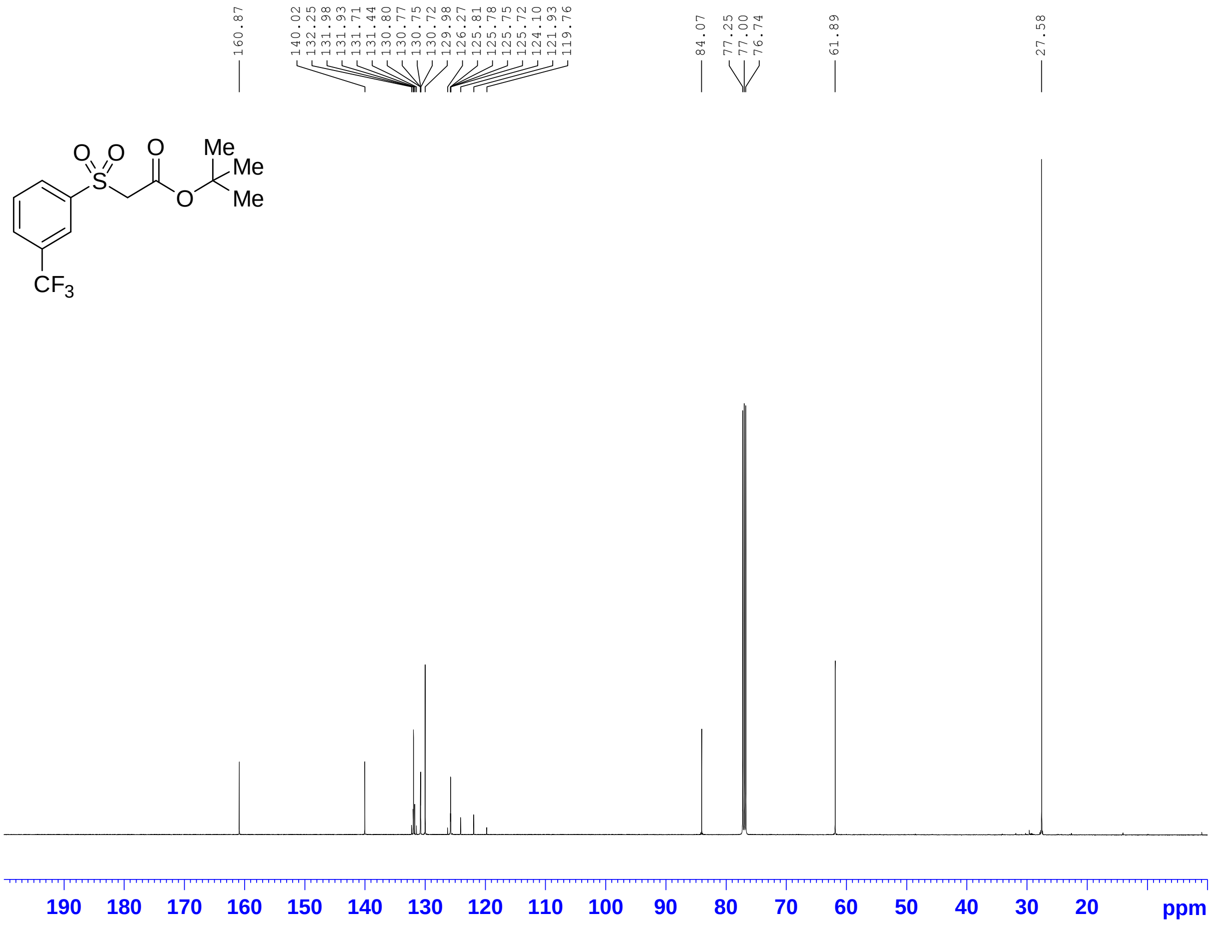
160.87
140.02
132.25
131.98
131.93
131.71
131.44
130.80
130.77
130.75
130.72
129.98
126.27
125.81
125.78
125.75
125.72
124.10
121.93
119.76
84.07
77.25
77.00
76.74
61.89
27.58
Me
Me
Me
O
O
O
S
O
CF3
190
180
170
160
150
140
130
120
110
100
90
80
70
60
50
40
30
20
ppm

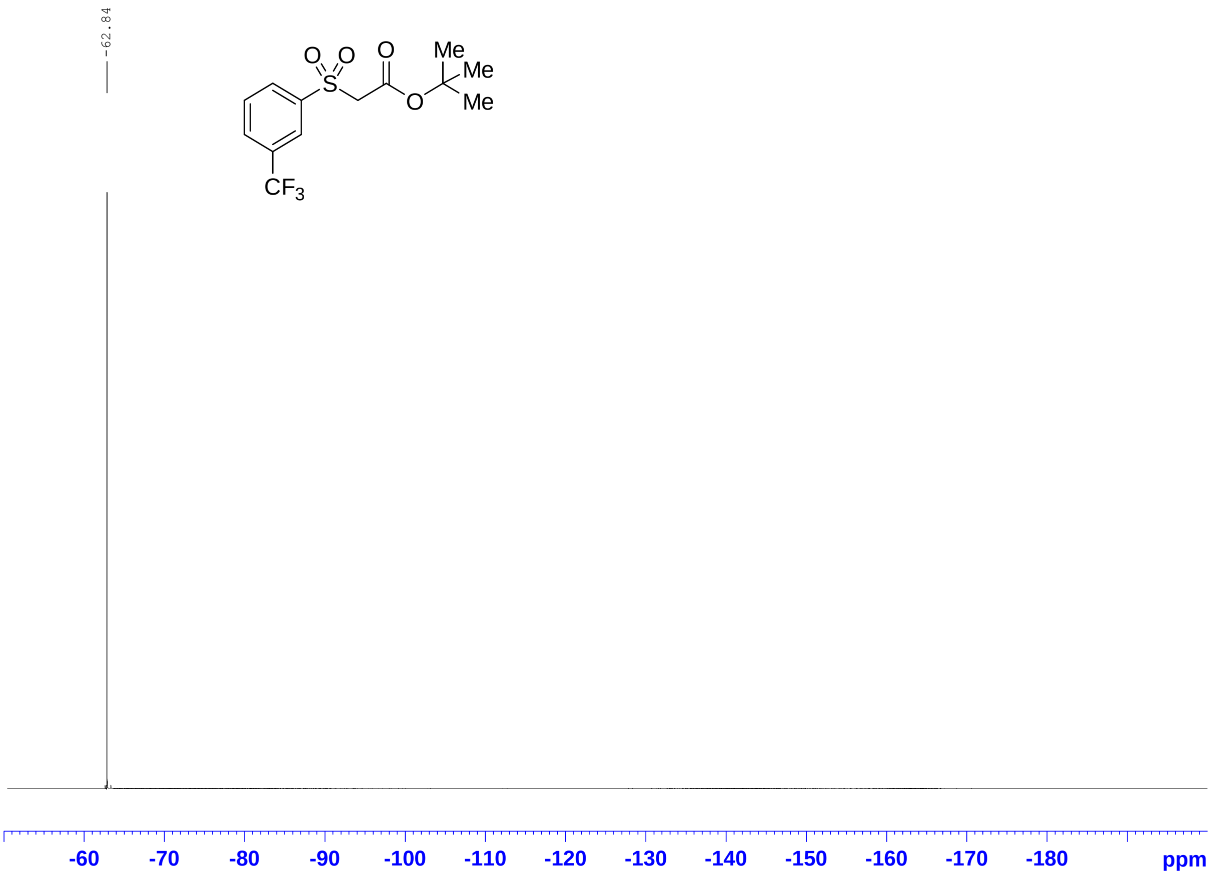
-62.84
O O O Me
S Me
O Me
CF3
-60 -70 -80 -90 -100 -110 -120 -130 -140 -150 -160 -170 -180 ppm

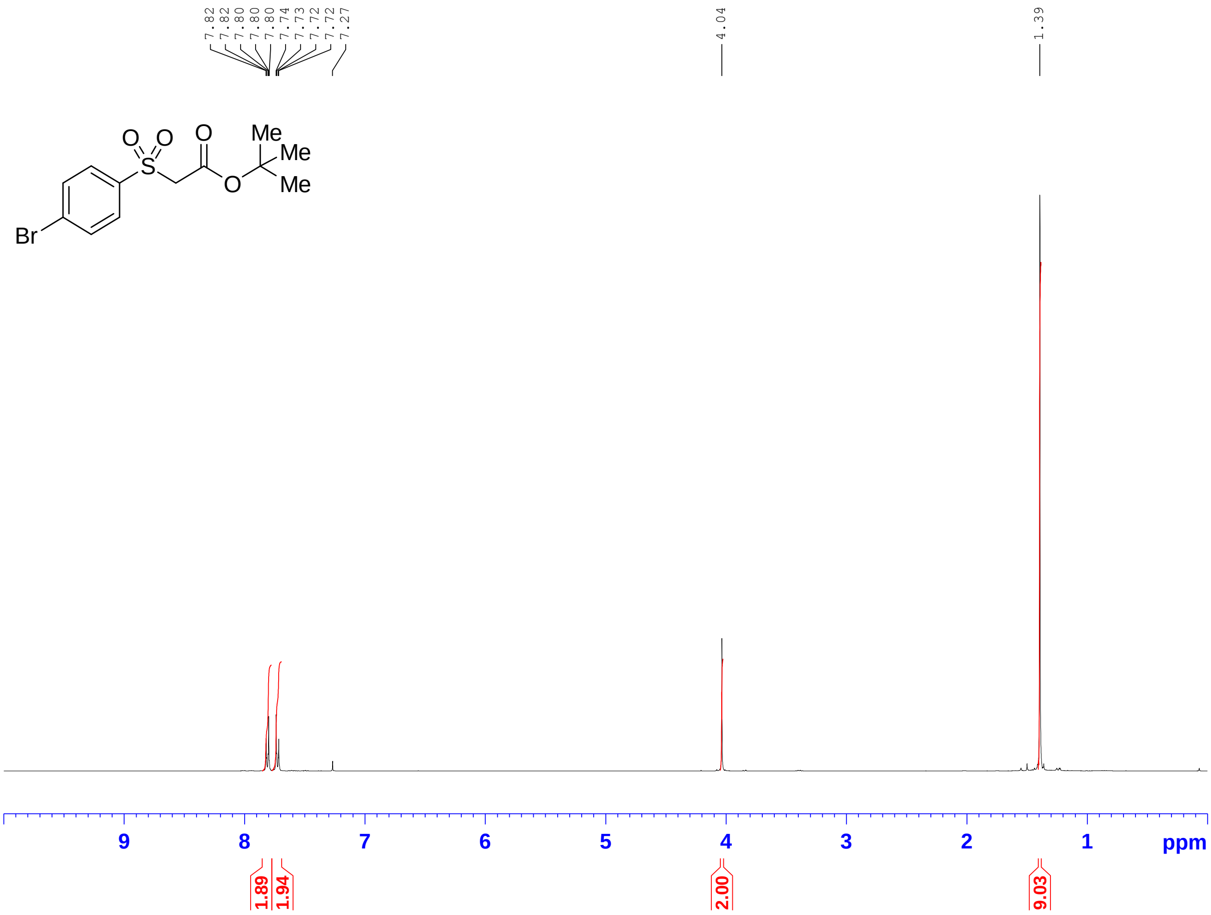
7.82
7.82
7.80
7.80
7.80
7.74
7.73
7.72
7.72
7.27
4.04
1.39
Br
O
O
S
O
O
Me
Me
Me
9
8
7
6
5
4
3
2
1
ppm
1.89
1.94
2.00
9.03

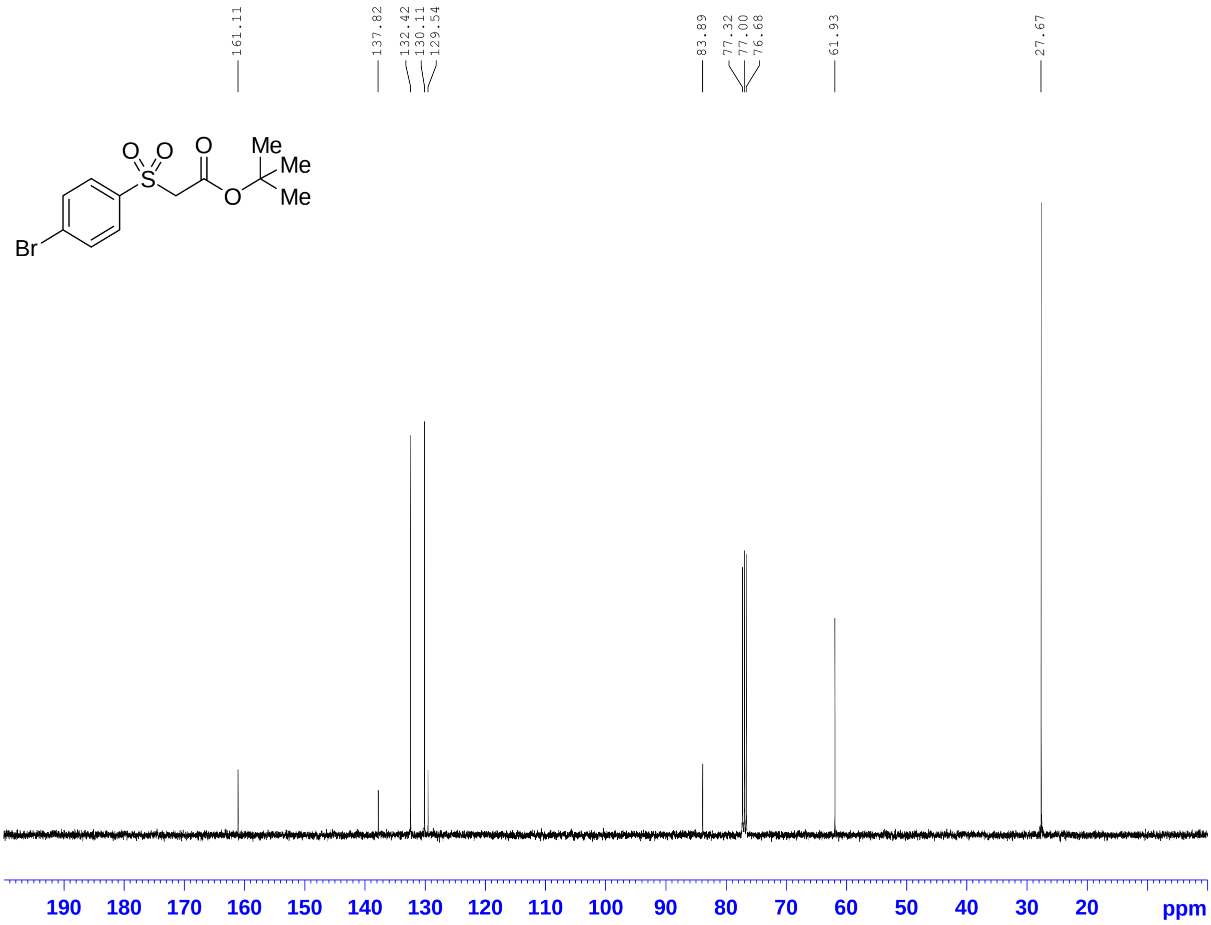
161.11
137.82
132.42
130.11
129.54
83.89
77.32
77.00
76.68
61.93
27.67
Br
O
O
S
O
O
Me
Me
Me
190
180
170
160
150
140
130
120
110
100
90
80
70
60
50
40
30
20
ppm

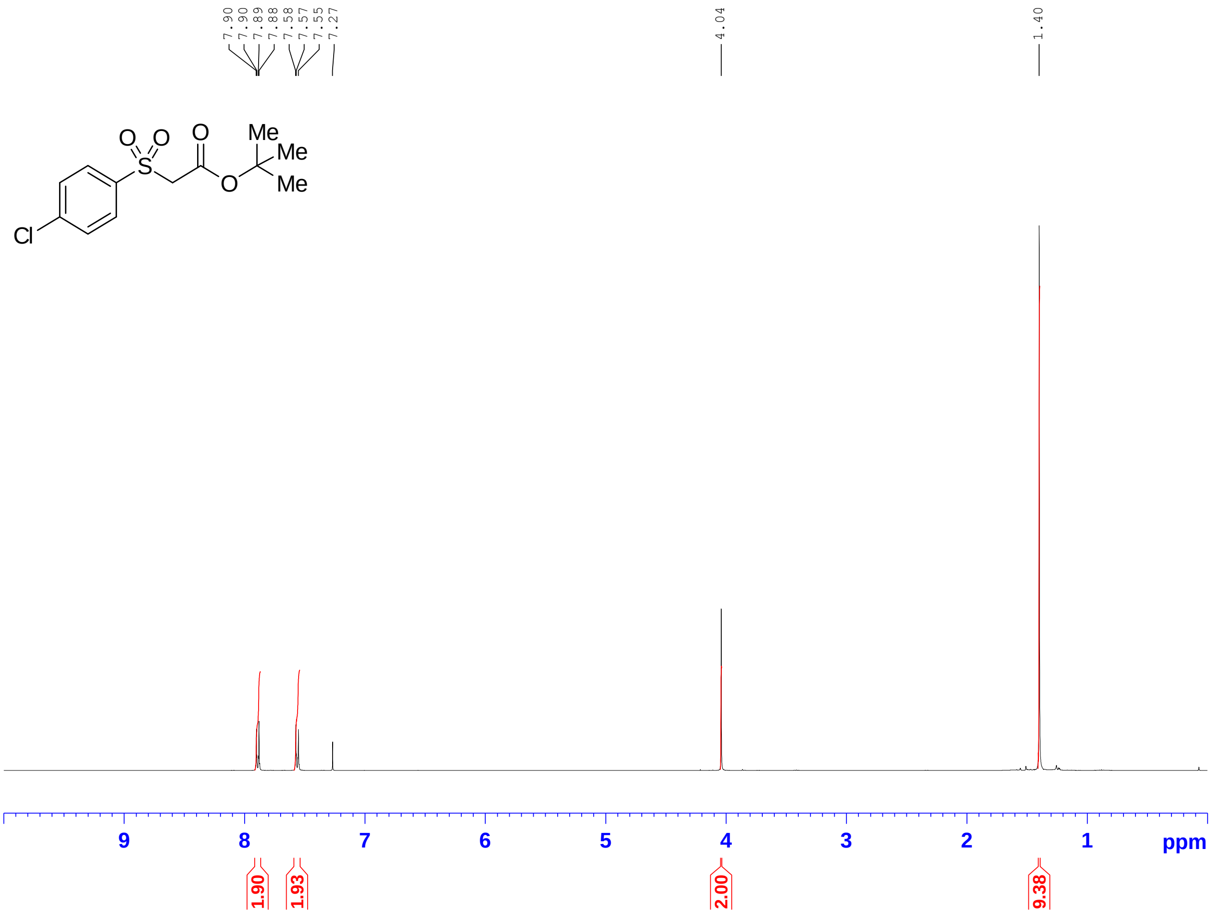
7.90
7.90
7.89
7.88
7.58
7.57
7.55
7.27
4.04
1.40
O
O
S
O
O
Me
Me
Me
Cl
9
8
7
6
5
4
3
2
1
ppm
1.90
1.93
2.00
9.38

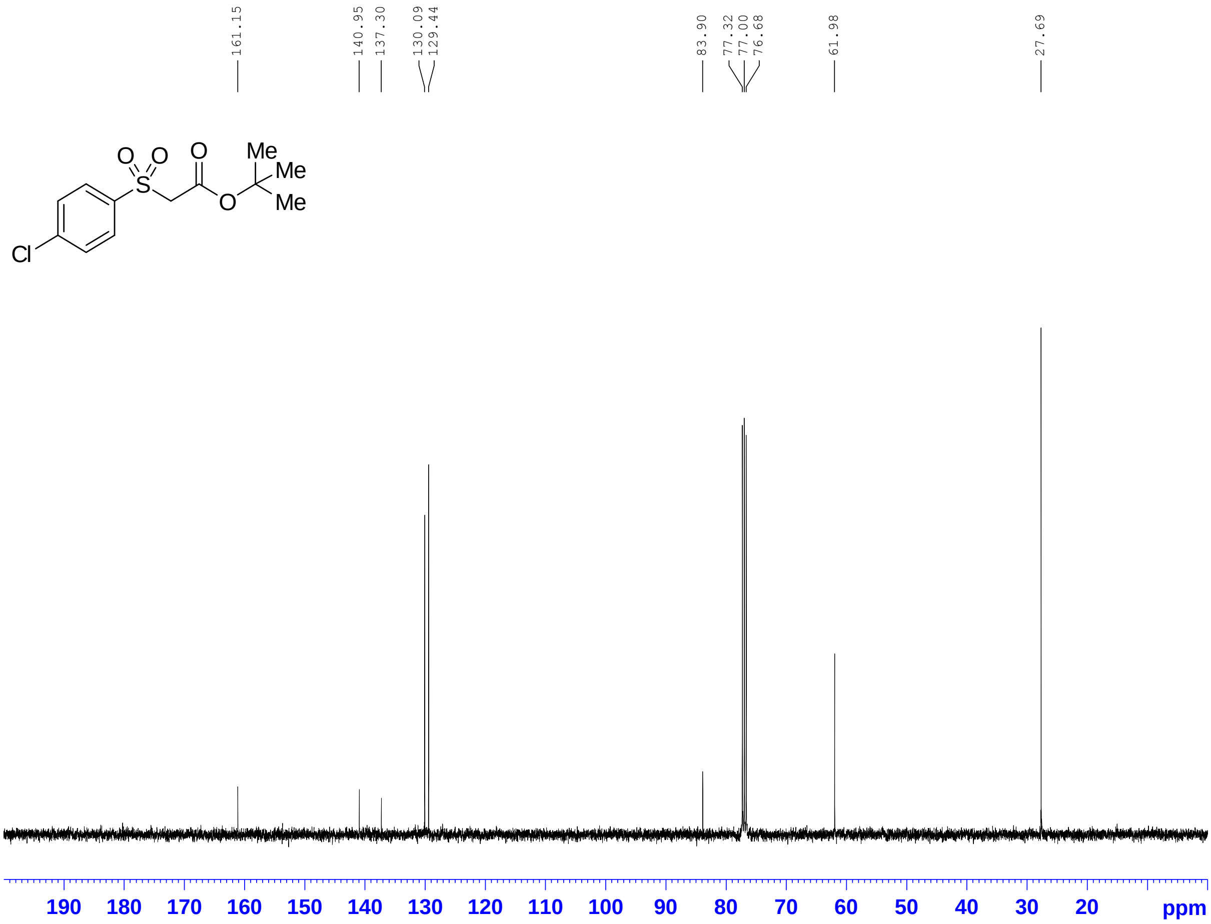
161.15
140.95
137.30
130.09
129.44
83.90
77.32
77.00
76.68
61.98
27.69
O
O
S
O
O
Me
Me
Me
Cl
190
180
170
160
150
140
130
120
110
100
90
80
70
60
50
40
30
20
ppm

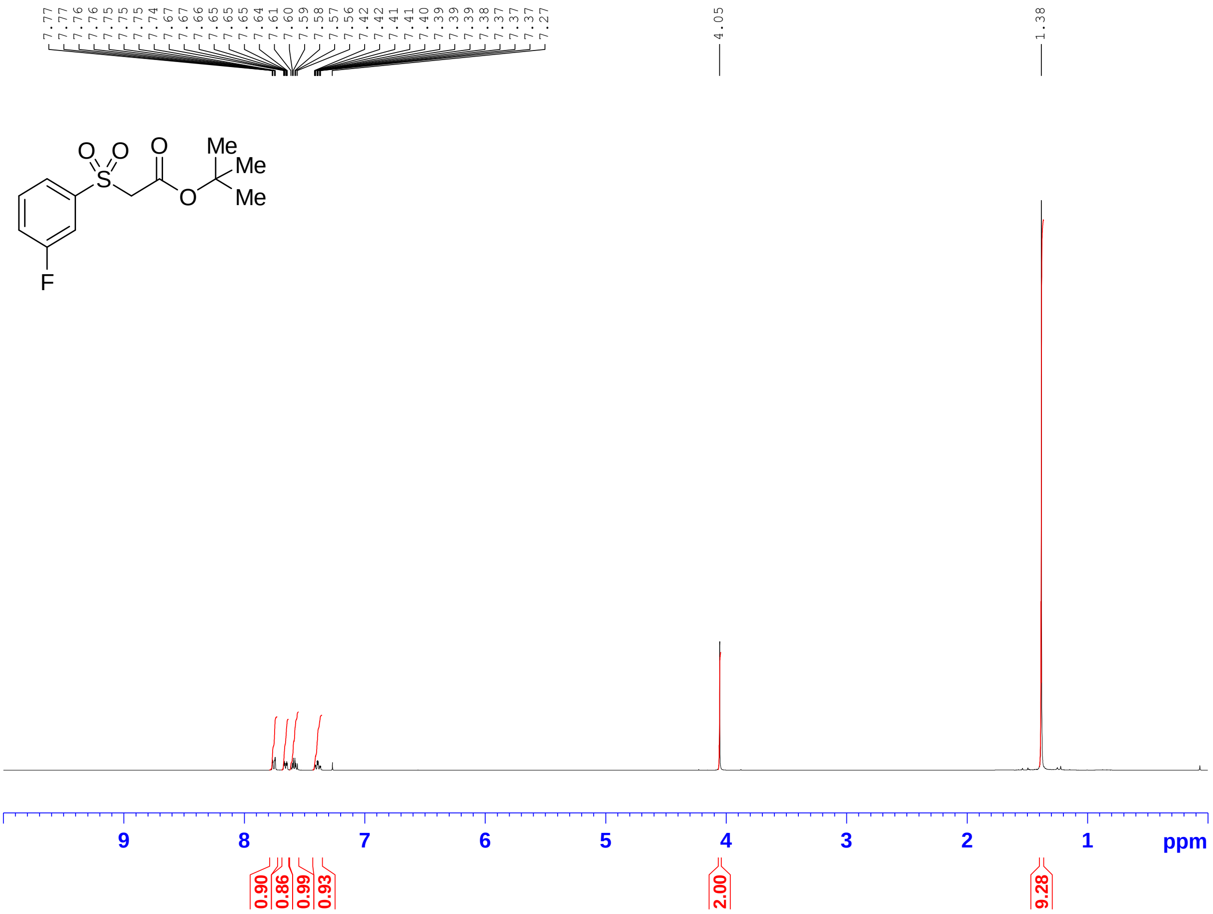
7.77
7.77
7.76
7.76
7.75
7.75
7.75
7.74
7.67
7.67
7.66
7.65
7.65
7.64
7.61
7.60
7.59
7.58
7.57
7.56
7.42
7.42
7.41
7.40
7.39
7.39
7.39
7.38
7.37
7.37
7.37
7.27
4.05
1.38
O
O
S
O
O
Me
Me
Me
F
9
8
7
6
5
4
3
2
1
ppm
0.90
0.86
0.99
0.93
2.00
9.28

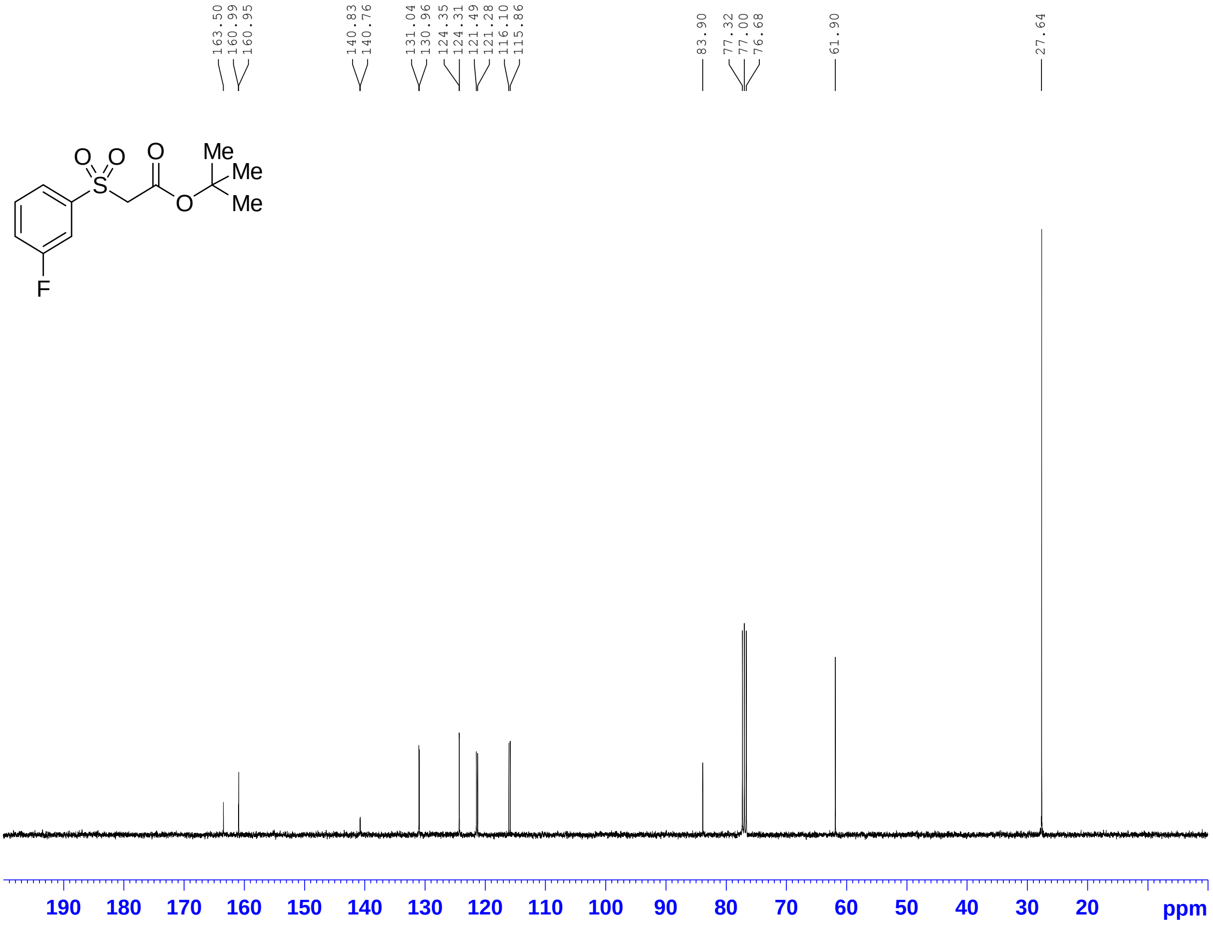
163.50
160.99
160.95
140.83
140.76
131.04
130.96
124.35
124.31
121.49
121.28
116.10
115.86
83.90
77.32
77.00
76.68
61.90
27.64
O
O
S
O
O
Me
Me
Me
F
190
180
170
160
150
140
130
120
110
100
90
80
70
60
50
40
30
20
ppm

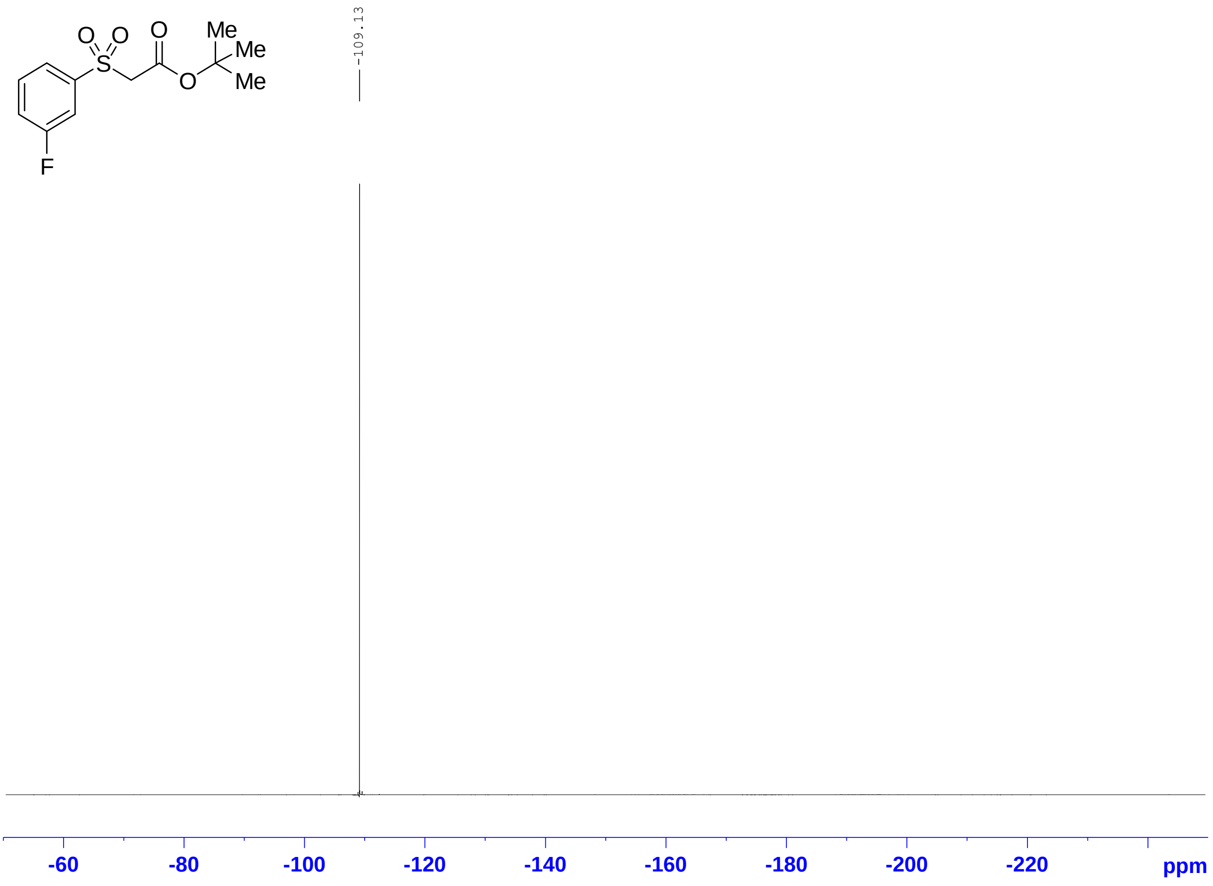

O
O
O
Me
Me
Me
S
O
F
-109.13
-60
-80
-100
-120
-140
-160
-180
-200
-220
ppm

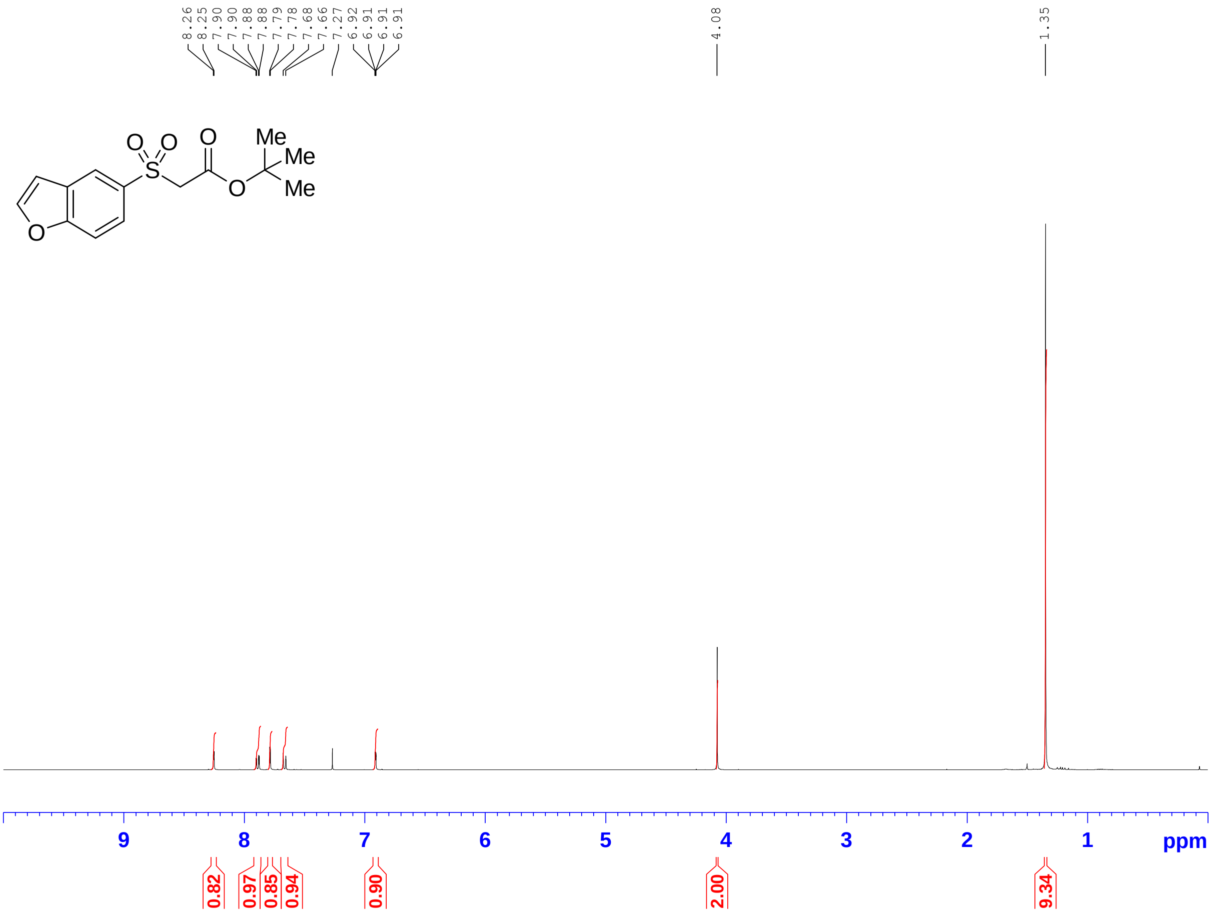
8.26
8.25
7.90
7.90
7.88
7.88
7.79
7.78
7.68
7.66
7.27
6.92
6.91
6.91
6.91
4.08
1.35
O O O Me Me Me S O O
9 8 7 6 5 4 3 2 1 ppm
0.82 0.97 0.85 0.94 0.90 2.00 9.34

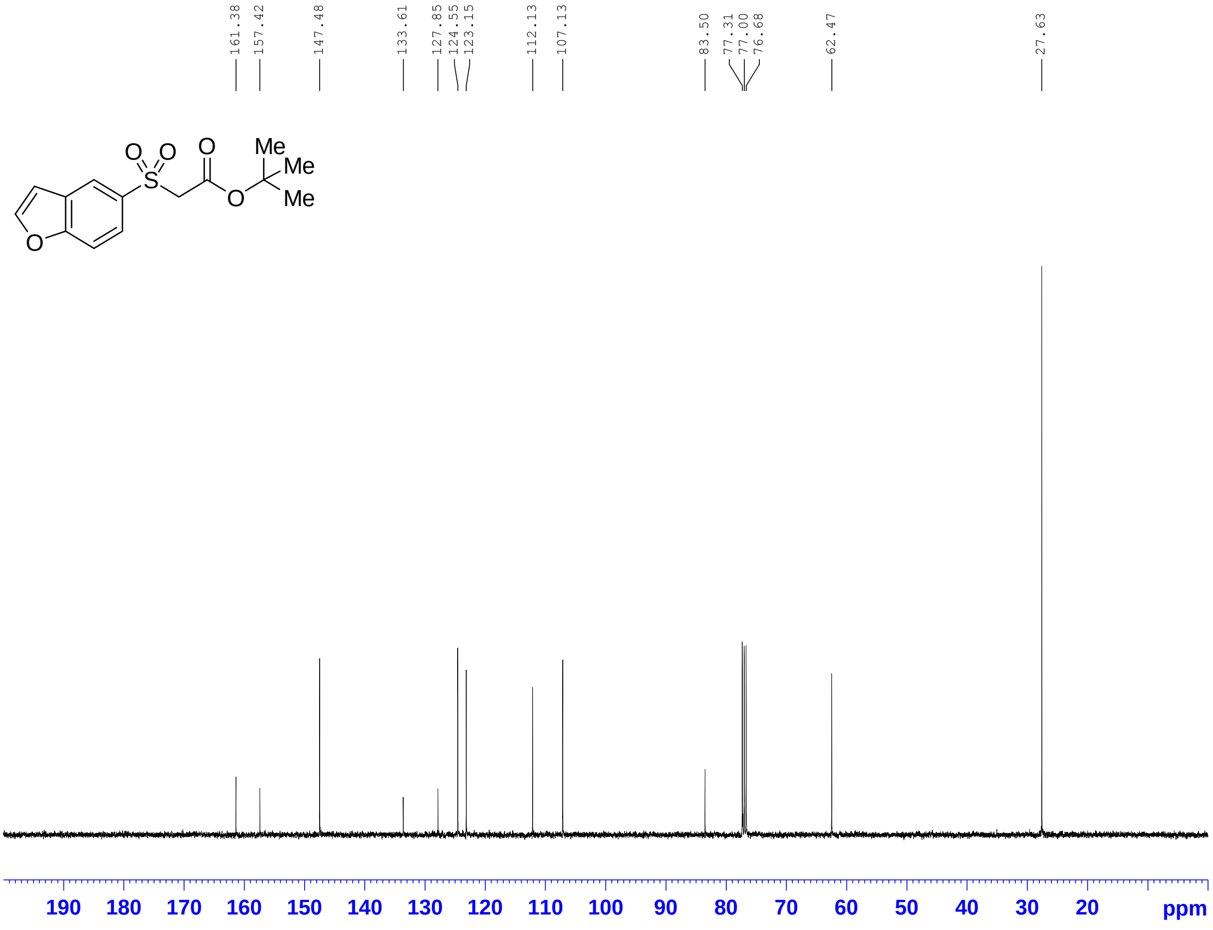
161.38
157.42
147.48
133.61
127.85
124.55
123.15
112.13
107.13
83.50
77.31
77.00
76.68
62.47
27.63
O O O Me Me Me S O O
190 180 170 160 150 140 130 120 110 100 90 80 70 60 50 40 30 20 ppm

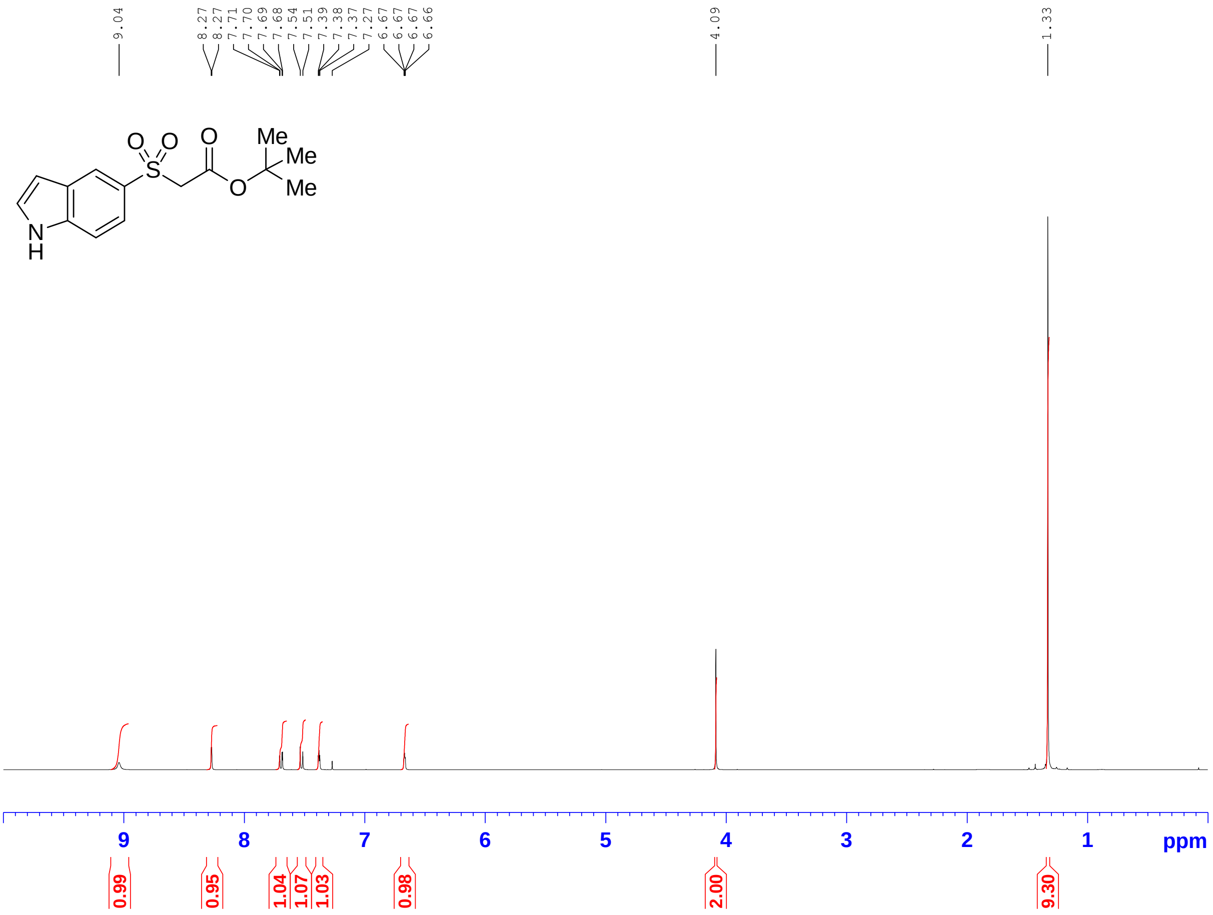
9.04
8.27
8.27
7.71
7.70
7.69
7.68
7.54
7.51
7.39
7.38
7.37
7.27
6.67
6.67
6.67
6.66
4.09
1.33
Me
Me
Me
N
H
O
S
O
O
O
0.99
0.95
1.04
1.07
1.03
0.98
2.00
9.30
ppm

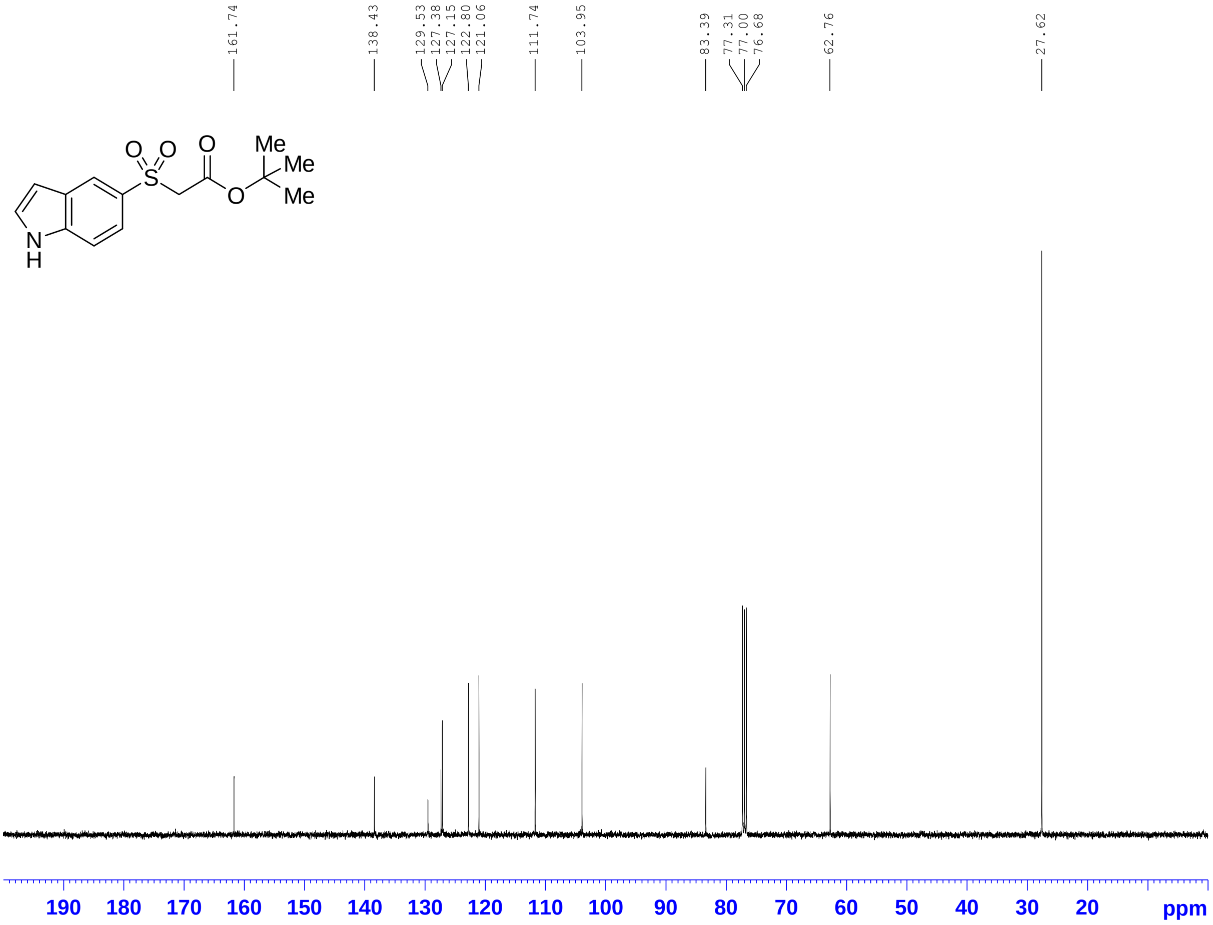
161.74
138.43
129.53
127.38
127.15
122.80
121.06
111.74
103.95
83.39
77.31
77.00
76.68
62.76
27.62
ppm

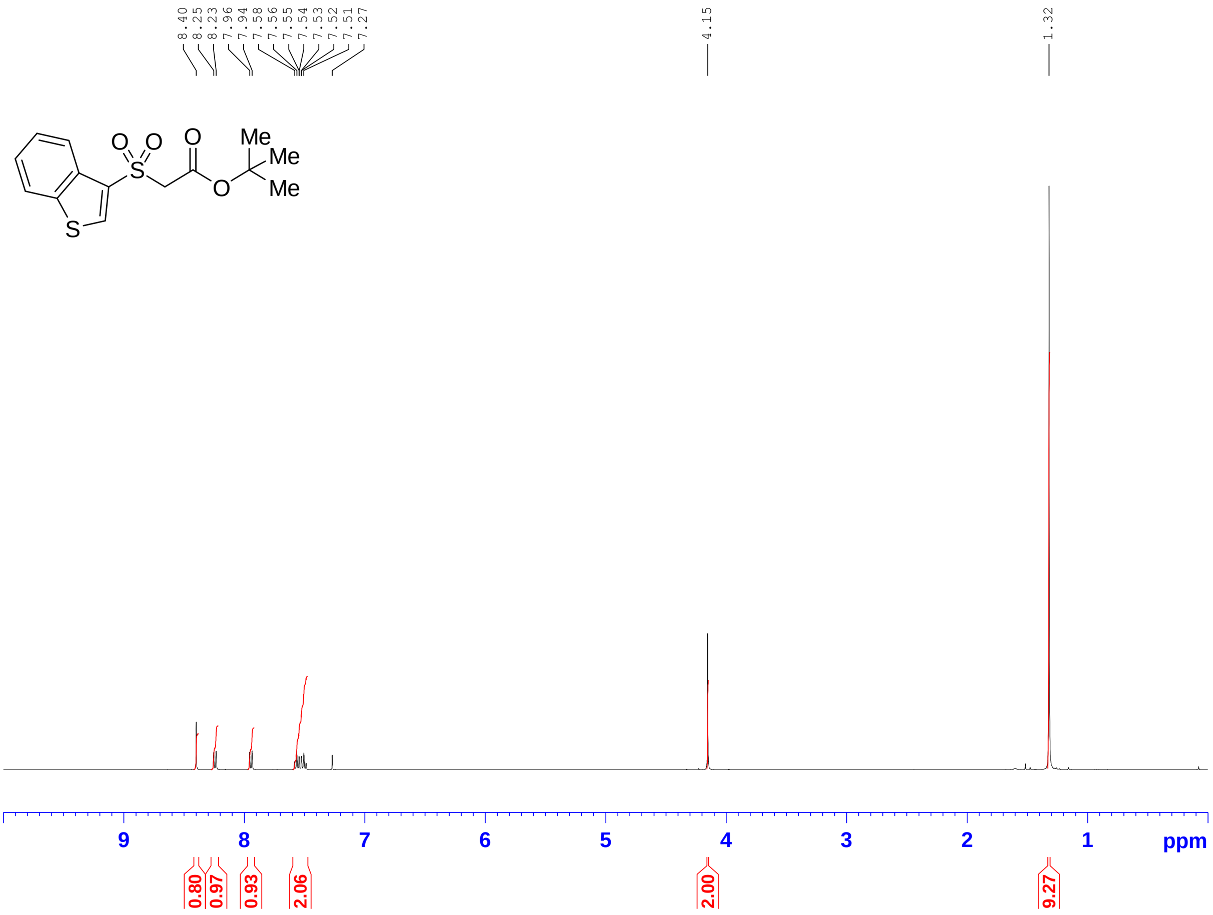
8.40
8.25
8.23
7.96
7.94
7.58
7.56
7.55
7.54
7.53
7.52
7.51
7.27
4.15
1.32
Me
Me
Me
O
O
O
O
S
S
9
8
7
6
5
4
3
2
1
ppm
0.80
0.97
0.93
2.06
2.00
9.27

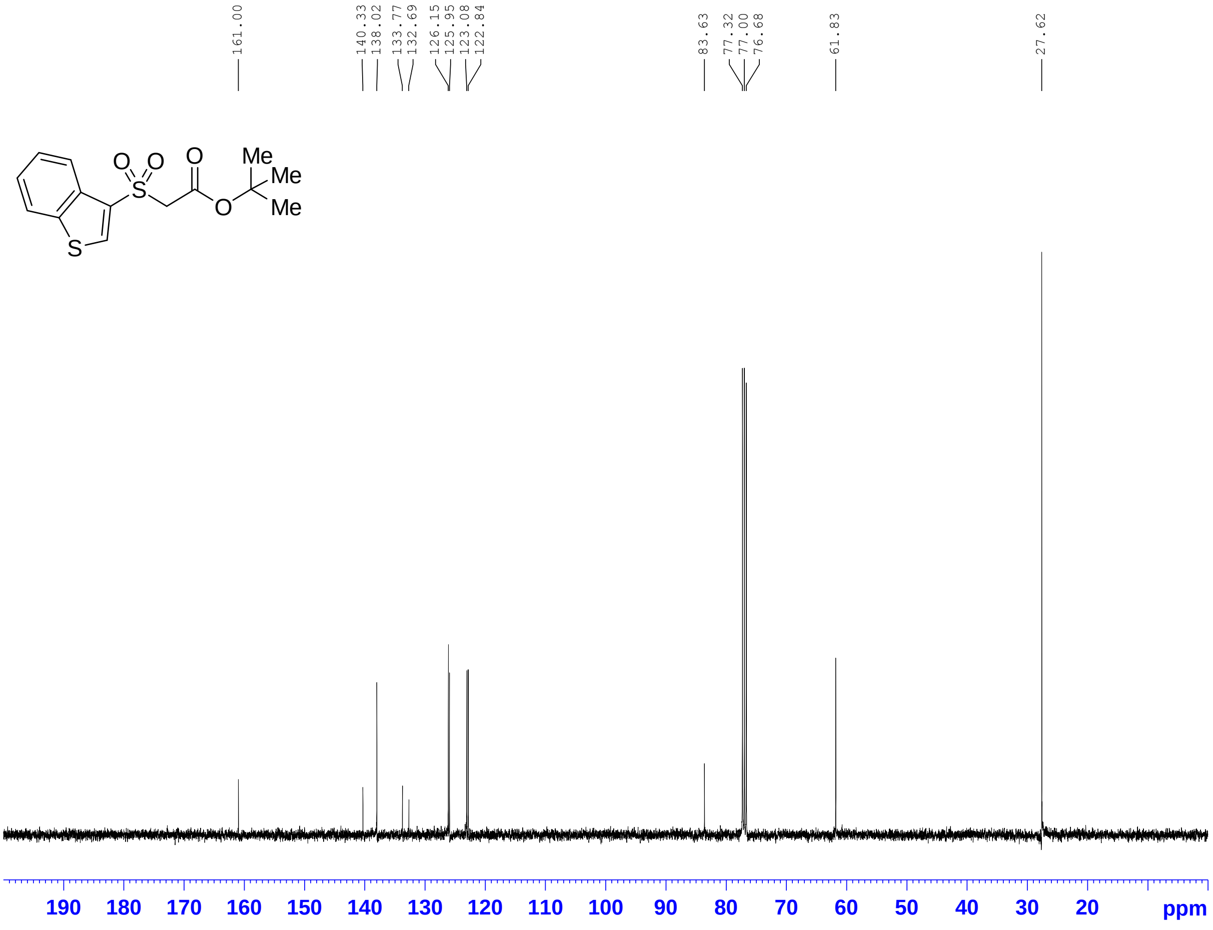
161.00
140.33
138.02
133.77
132.69
126.15
125.95
123.08
122.84
83.63
77.32
77.00
76.68
61.83
27.62
190
180
170
160
150
140
130
120
110
100
90
80
70
60
50
40
30
20
ppm

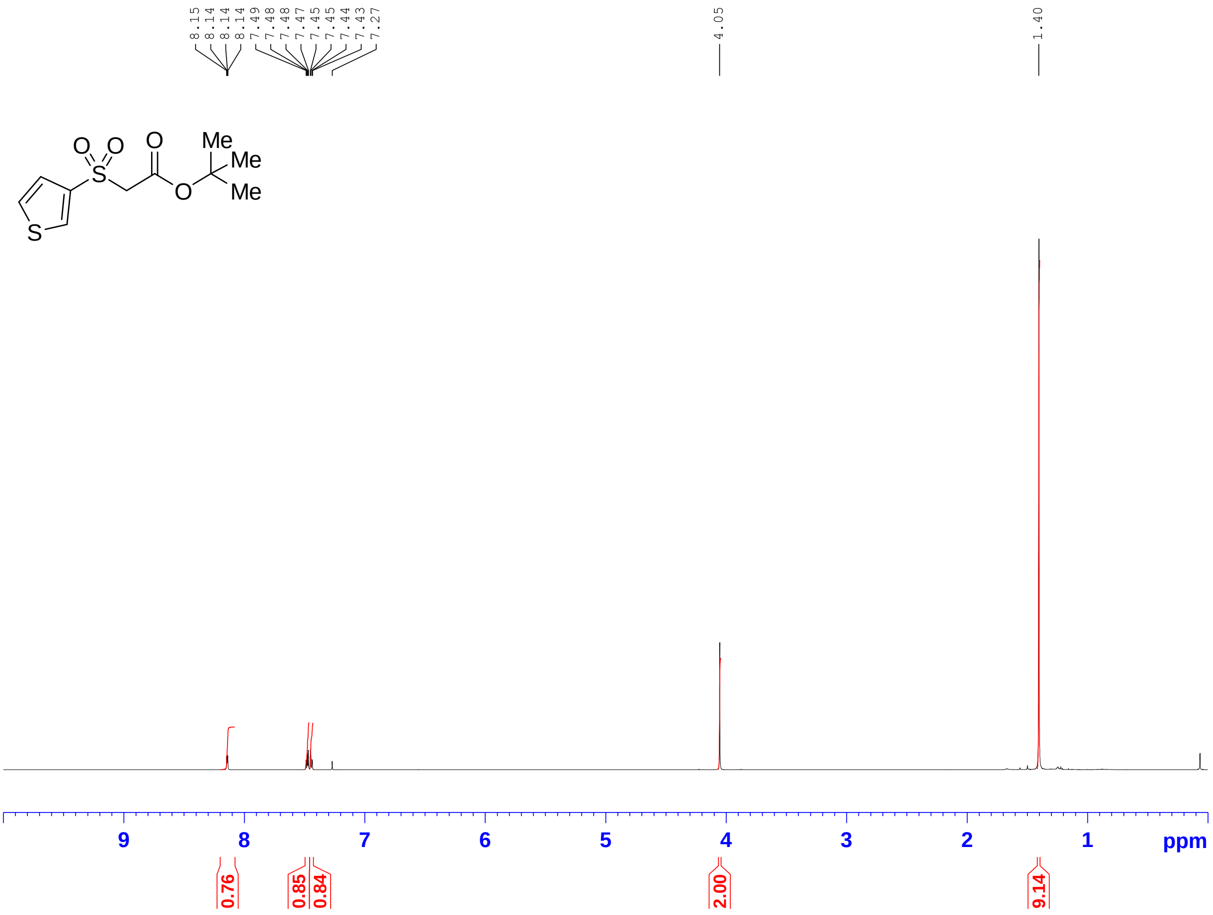
8.15
8.14
8.14
8.14
7.49
7.48
7.48
7.47
7.45
7.45
7.44
7.43
7.27
4.05
1.40
O O O Me Me Me S O S
9 8 7 6 5 4 3 2 1 ppm
0.76 0.85 0.84 2.00 9.14

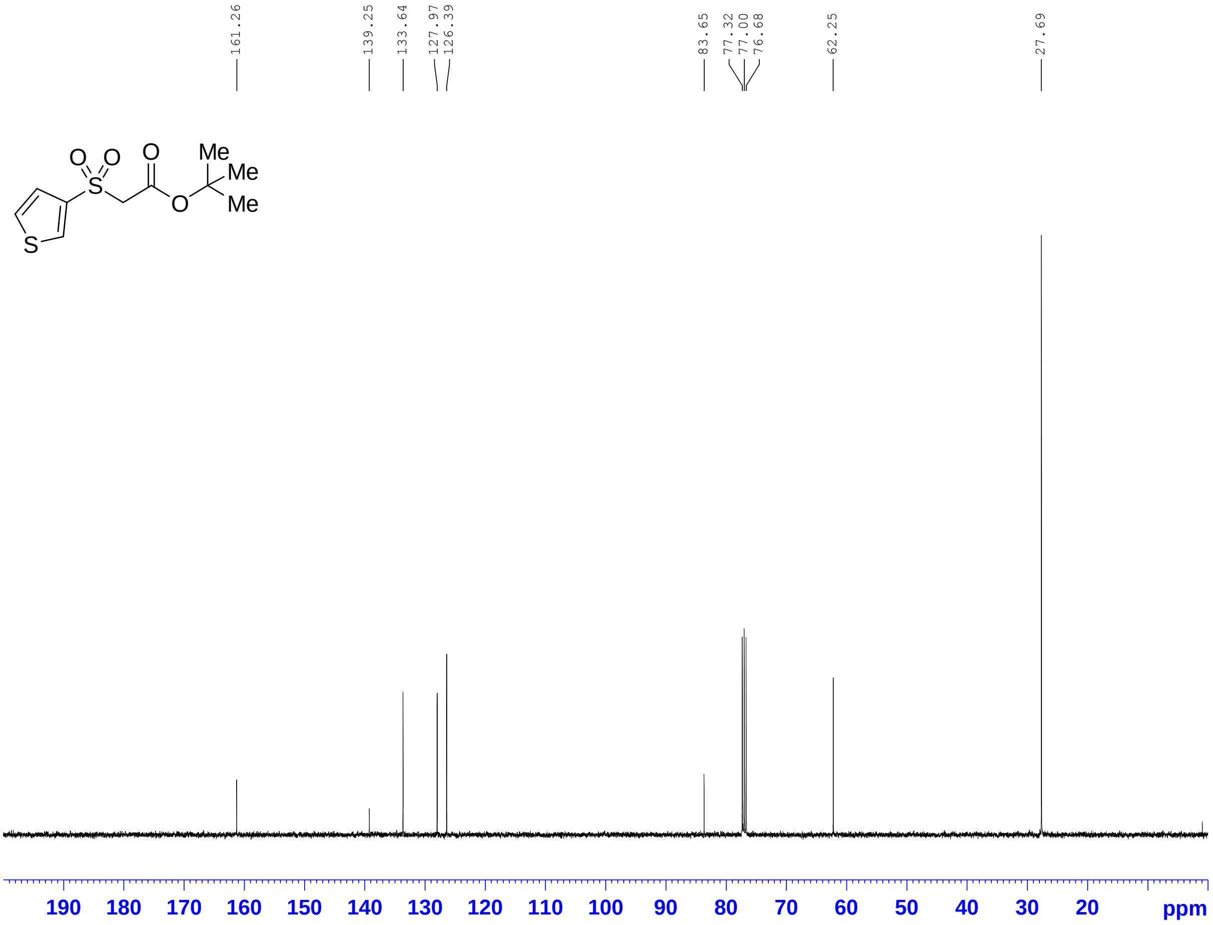
161.26
139.25
133.64
127.97
126.39
83.65
77.32
77.00
76.68
62.25
27.69
O O O Me Me Me S O S
190 180 170 160 150 140 130 120 110 100 90 80 70 60 50 40 30 20 ppm

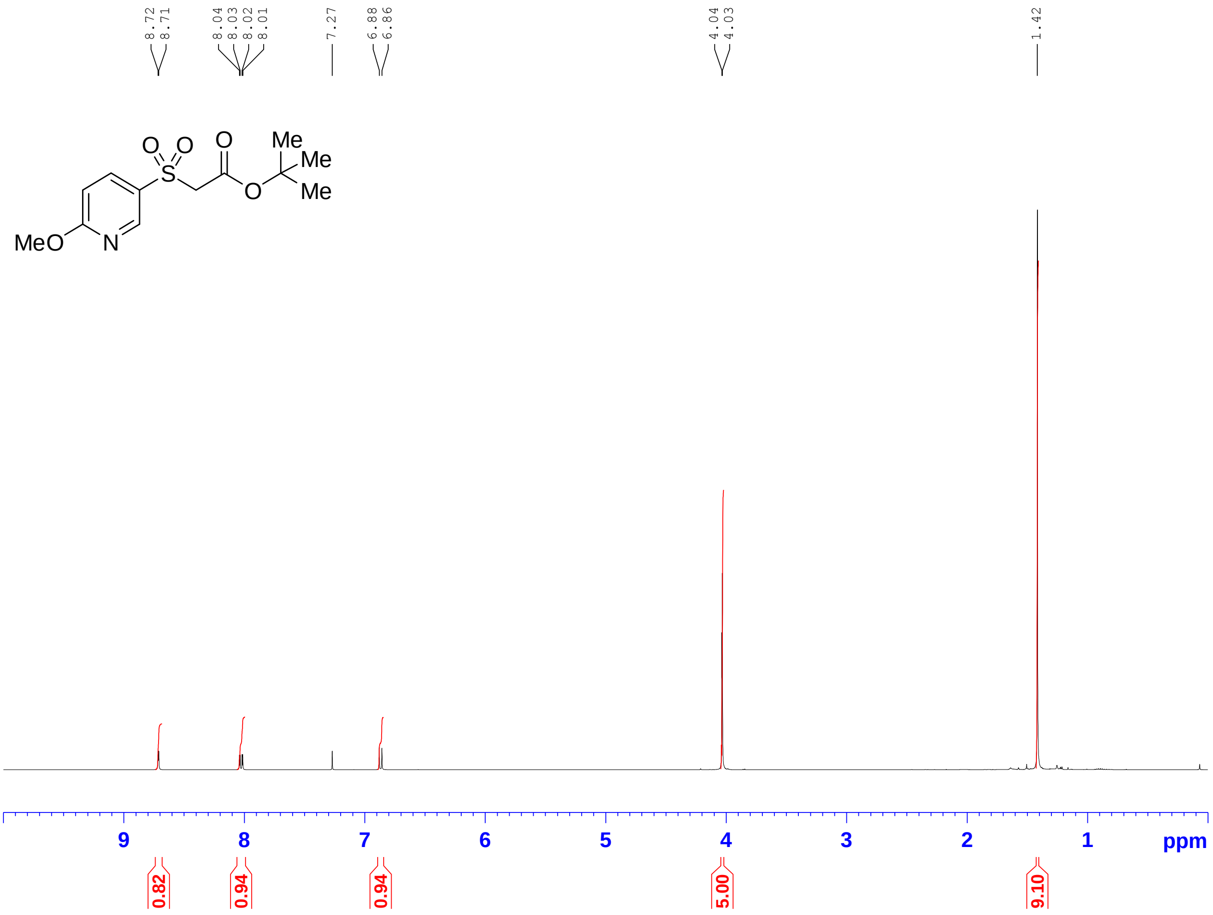
8.72
8.71
8.04
8.03
8.02
8.01
7.27
6.88
6.86
4.04
4.03
1.42
MeO
N
O
O
S
O
O
Me
Me
Me
9
8
7
6
5
4
3
2
1
ppm
0.82
0.94
0.94
5.00
9.10

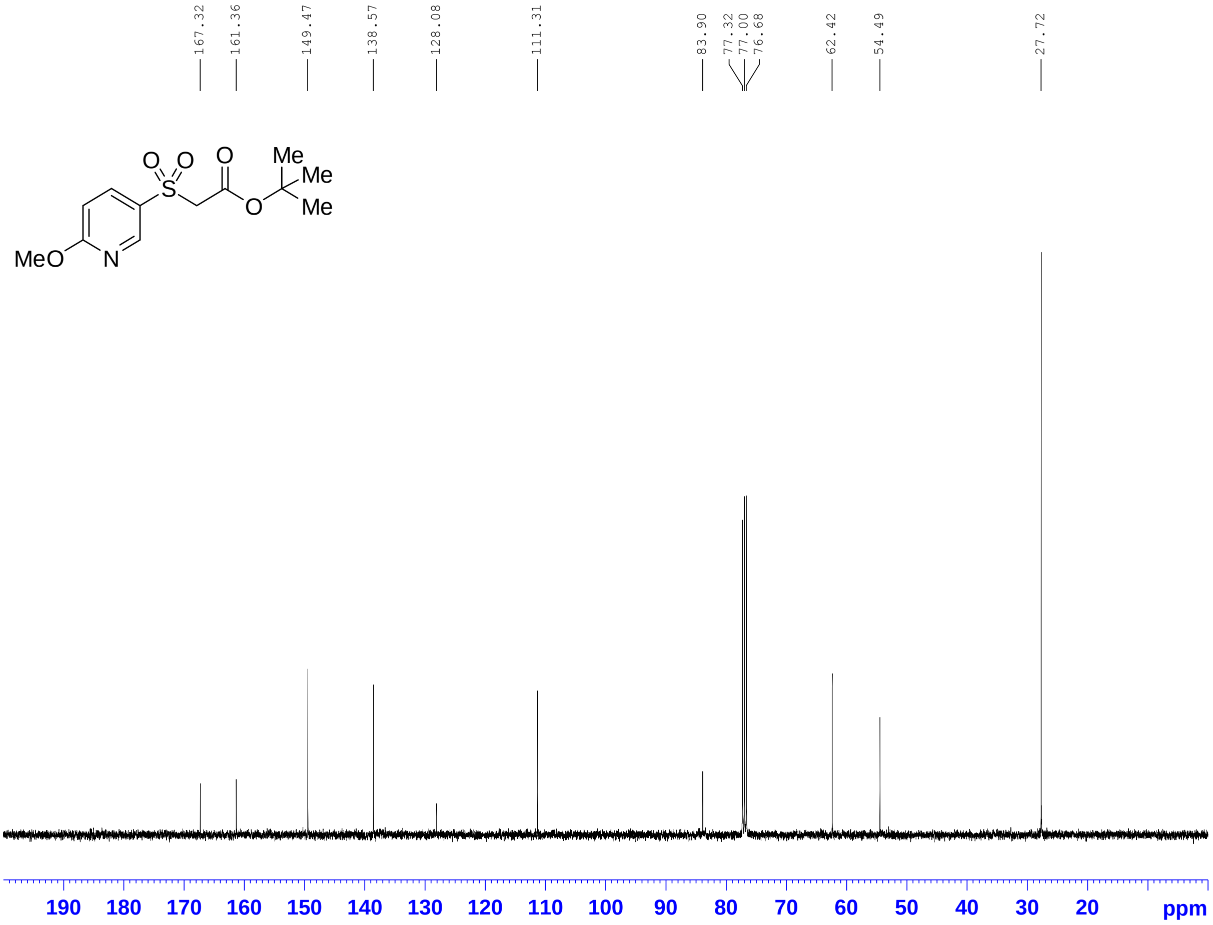
167.32
161.36
149.47
138.57
128.08
111.31
83.90
77.32
77.00
76.68
62.42
54.49
27.72
MeO
N
O
O
S
O
O
Me
Me
Me
190
180
170
160
150
140
130
120
110
100
90
80
70
60
50
40
30
20
ppm

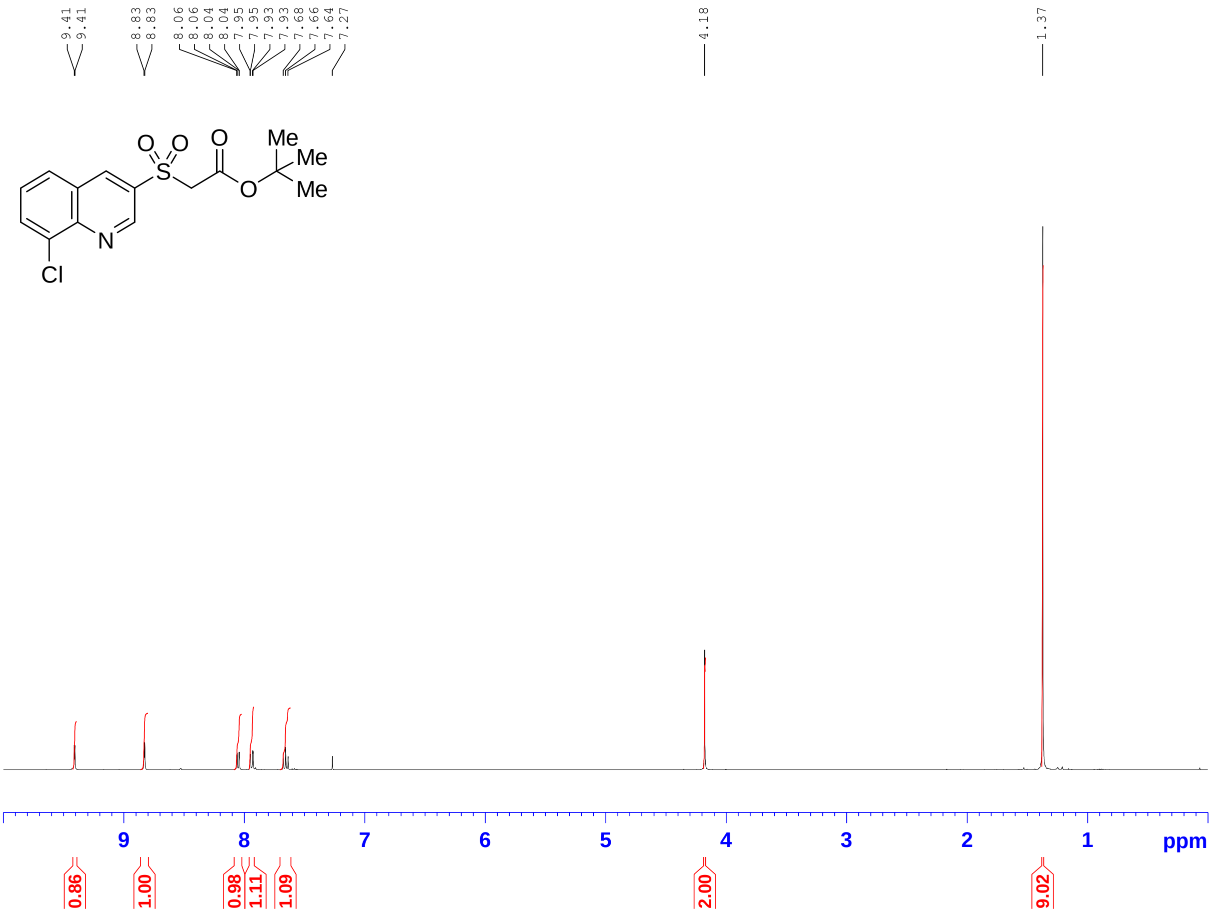
9.41
9.41
8.83
8.83
8.06
8.06
8.04
8.04
7.95
7.95
7.93
7.93
7.68
7.66
7.64
7.27
4.18
1.37
O
O
S
O
O
Me
Me
Me
N
Cl
9
8
7
6
5
4
3
2
1
ppm
0.86
1.00
0.98
1.11
1.09
2.00
9.02

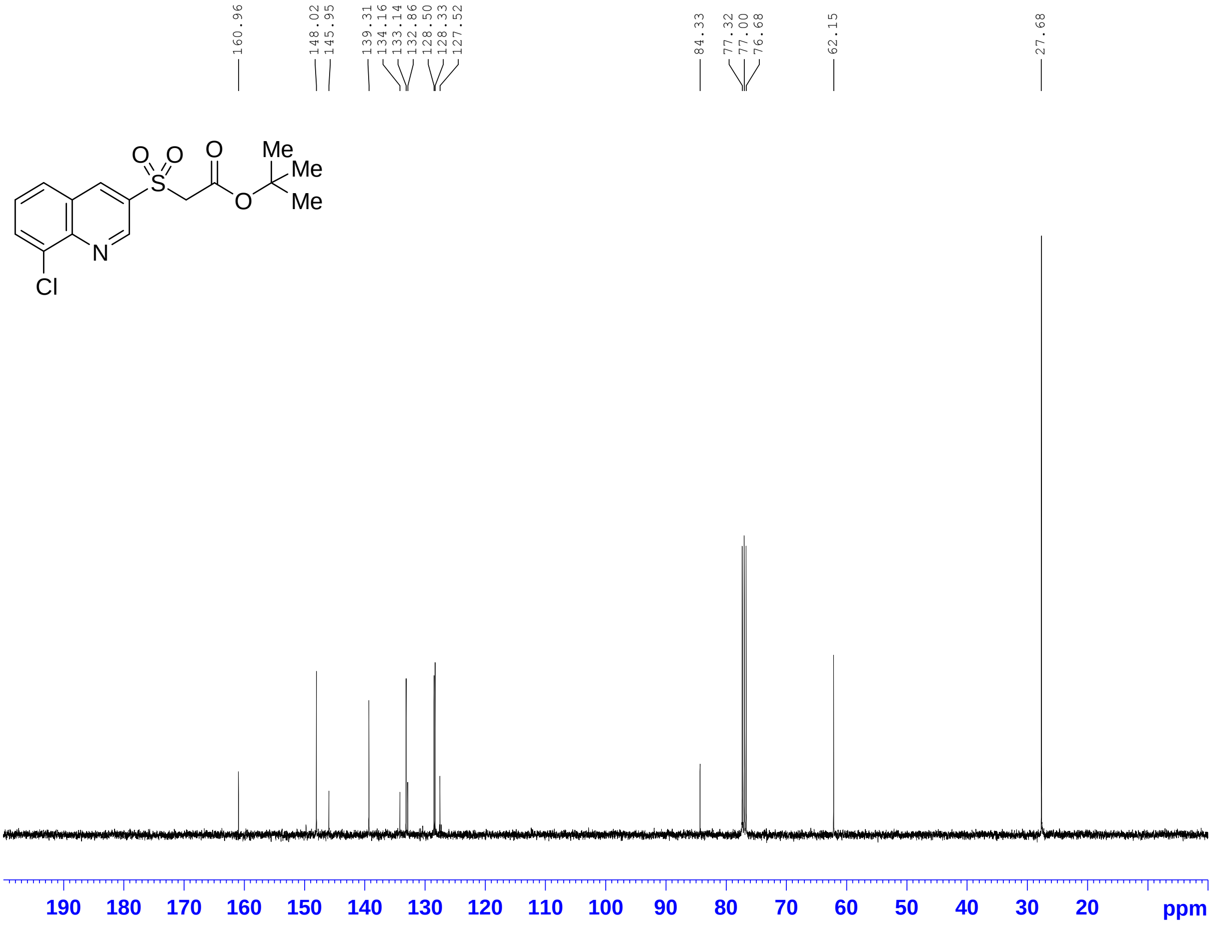
160.96
148.02
145.95
139.31
134.16
133.14
132.86
128.50
128.33
127.52
84.33
77.32
77.00
76.68
62.15
27.68
O
O
S
O
O
Me
Me
Me
N
Cl
190
180
170
160
150
140
130
120
110
100
90
80
70
60
50
40
30
20
ppm

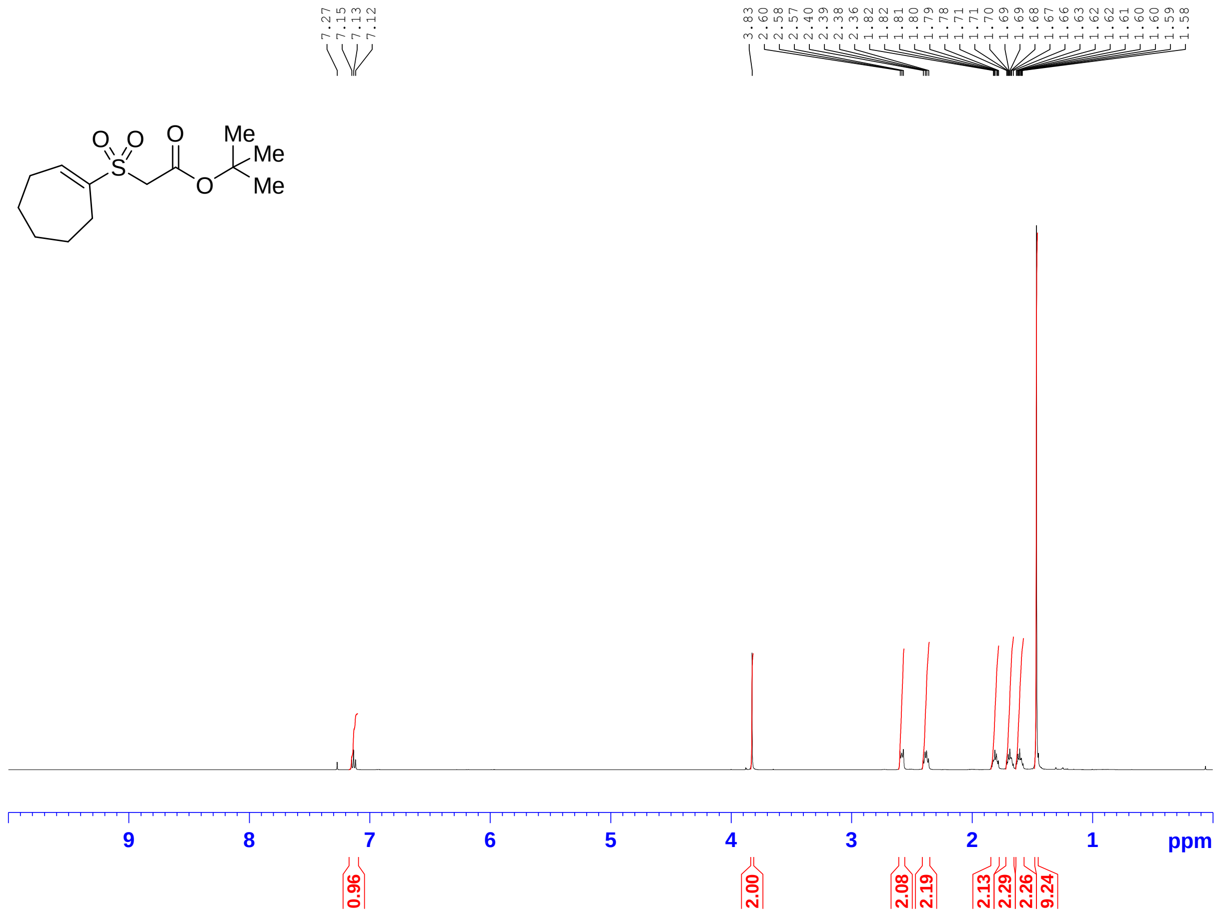

7.27
7.15
7.13
7.12
3.83
2.60
2.58
2.57
2.40
2.39
2.38
2.36
1.82
1.82
1.81
1.80
1.79
1.78
1.71
1.71
1.70
1.69
1.69
1.68
1.67
1.66
1.63
1.62
1.62
1.61
1.60
1.59
1.58
O
O
S
O
O
Me
Me
Me
9
8
7
6
5
4
3
2
1
ppm
0.96
2.00
2.08
2.19
2.13
2.29
2.26
9.24

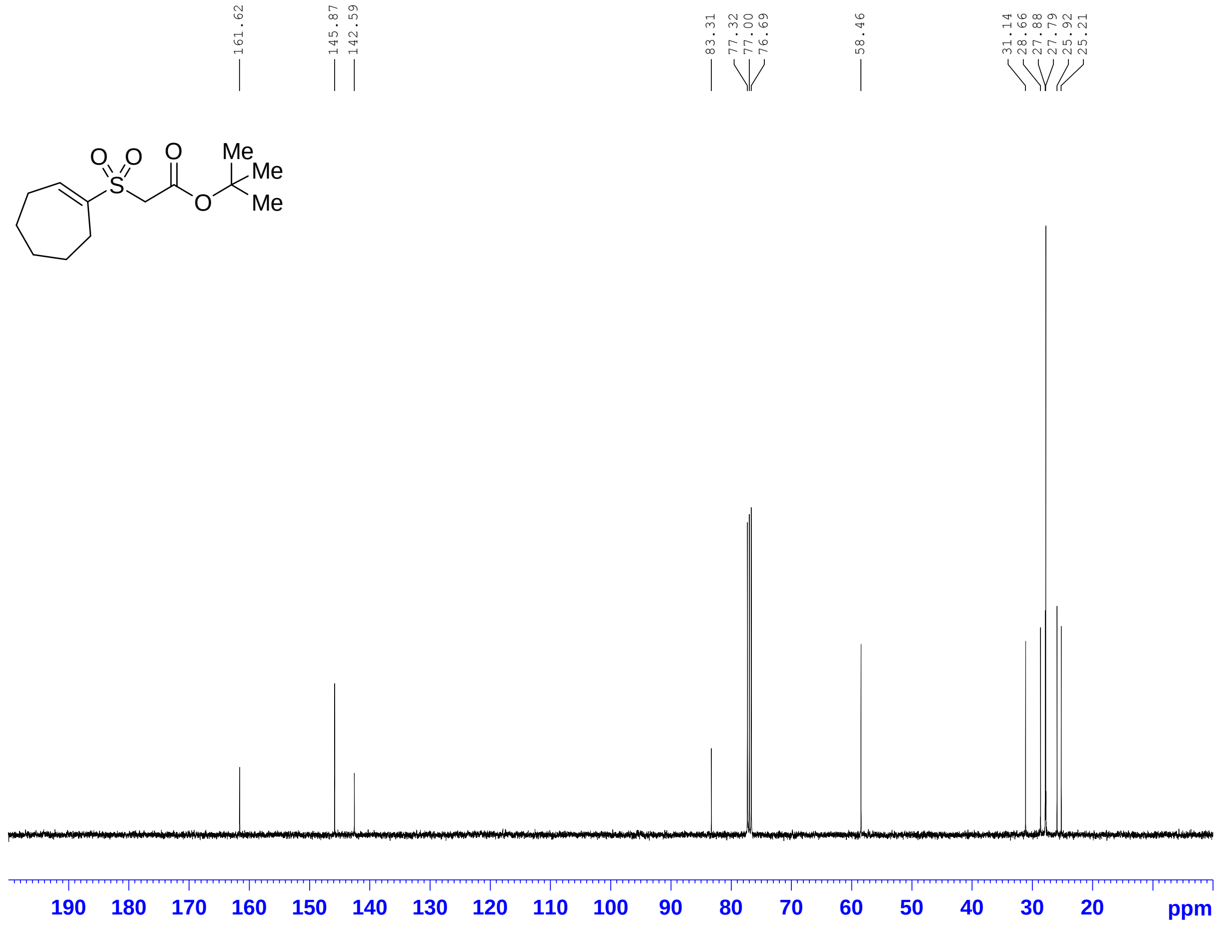

161.62
145.87
142.59
83.31
77.32
77.00
76.69
58.46
31.14
28.66
27.88
27.79
25.92
25.21
O
O
S
O
O
Me
Me
Me
190
180
170
160
150
140
130
120
110
100
90
80
70
60
50
40
30
20
ppm

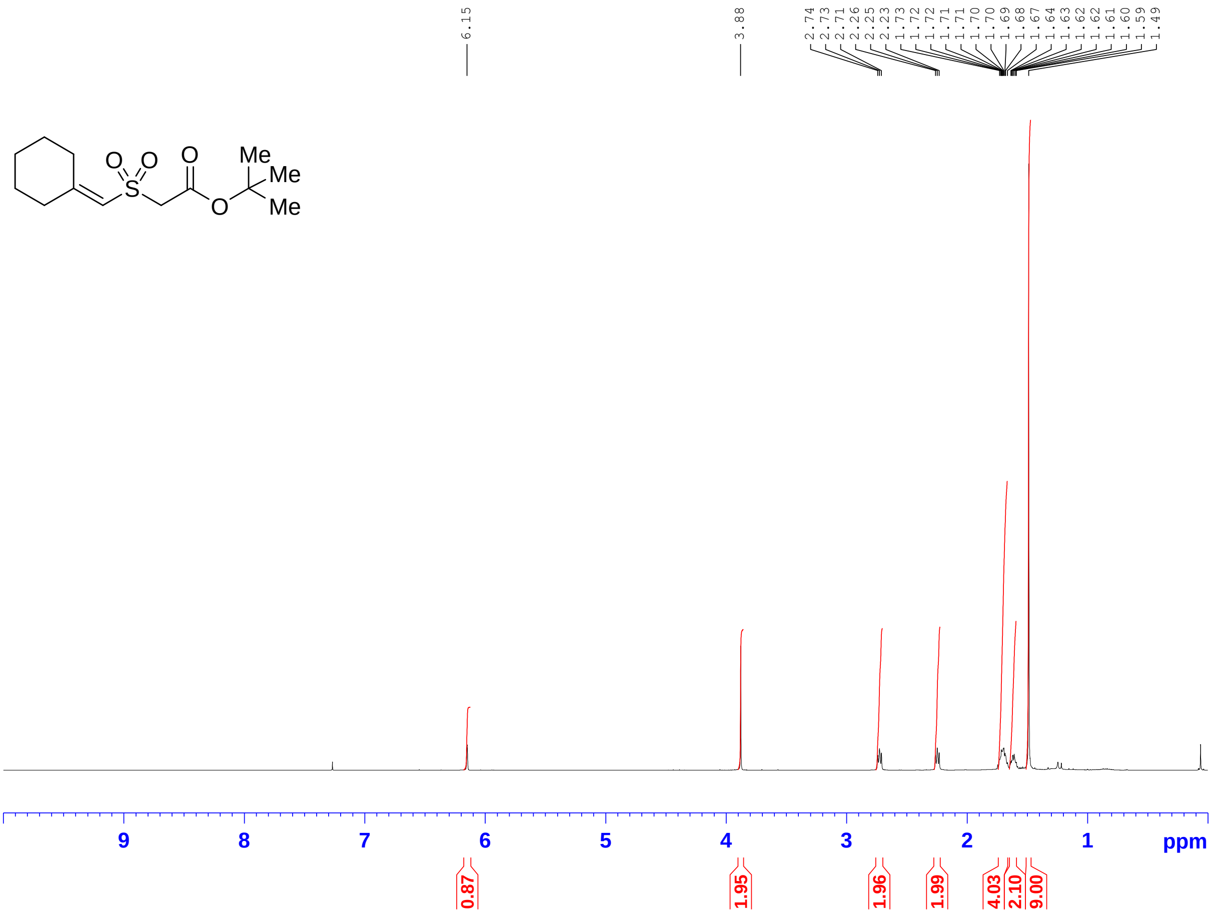
6.15
3.88
2.74 2.73 2.71 2.26 2.25 2.23 1.73 1.72 1.72 1.71 1.71 1.70 1.70 1.69 1.68 1.67 1.64 1.63 1.62 1.62 1.61 1.60 1.59 1.49
O O O Me Me Me S O
9 8 7 6 5 4 3 2 1 ppm
0.87 1.95 1.96 1.99 4.03 2.10 9.00

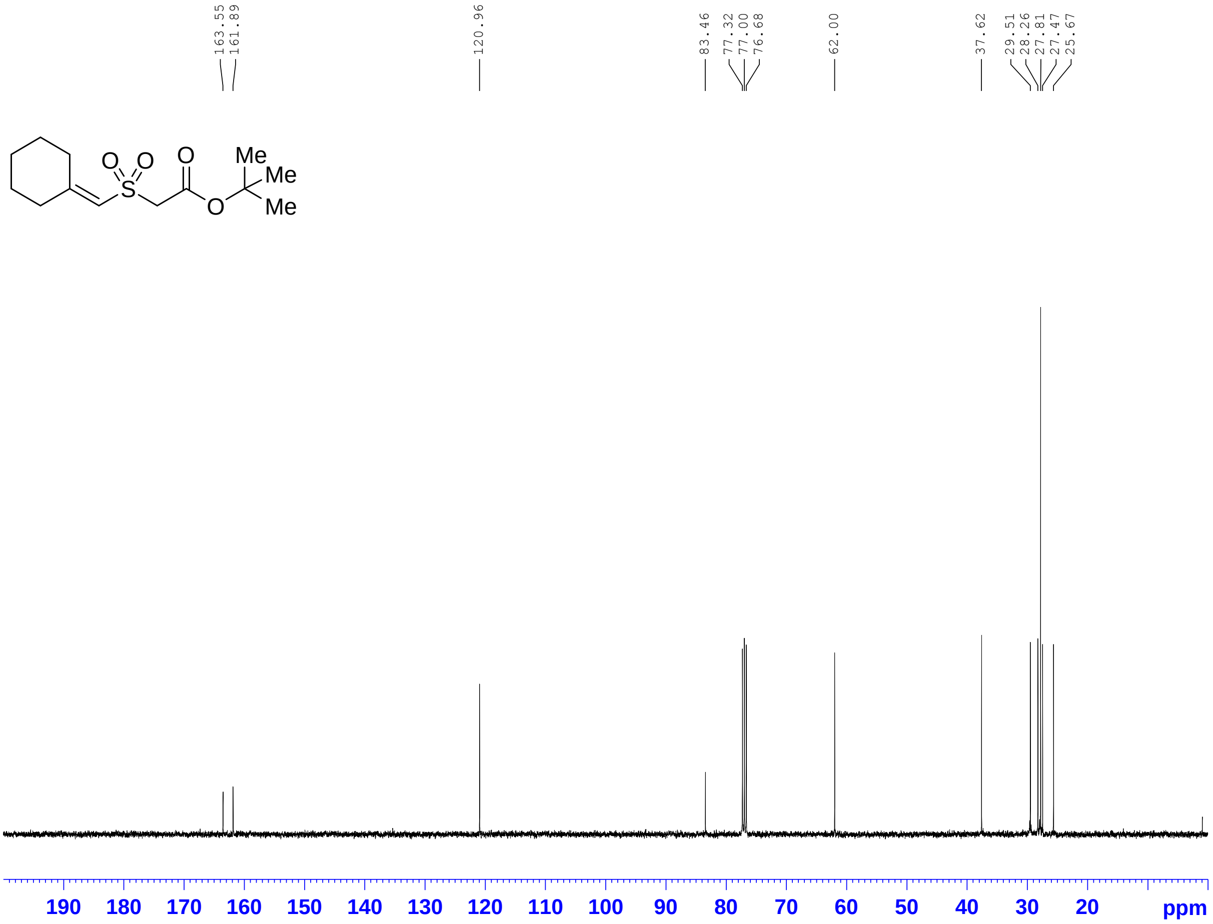
163.55 161.89
120.96
83.46 77.32 77.00 76.68
62.00
37.62
29.51 28.26 27.81 27.47 25.67
O O O Me Me Me S O
190 180 170 160 150 140 130 120 110 100 90 80 70 60 50 40 30 20 ppm

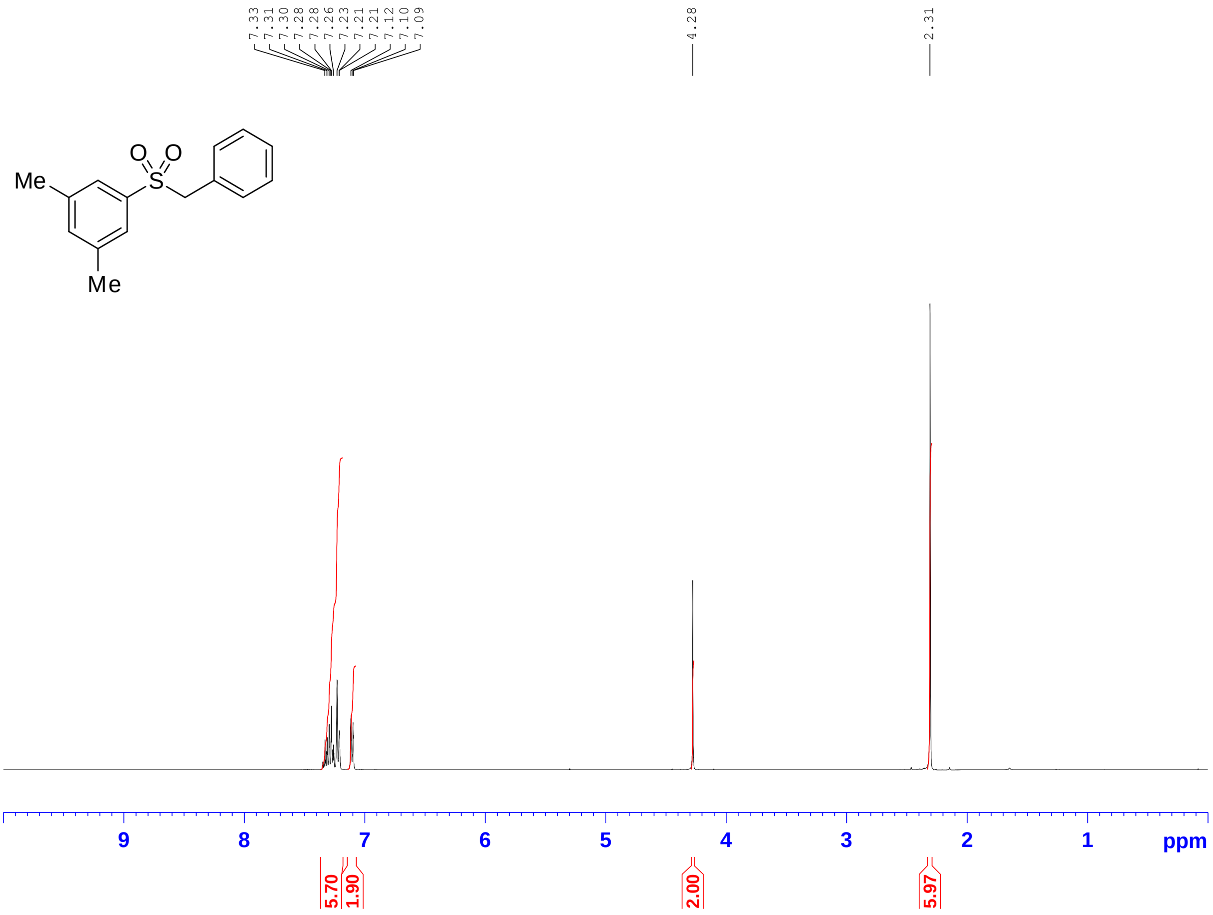
7.33
7.31
7.30
7.28
7.28
7.26
7.23
7.21
7.21
7.12
7.10
7.09
4.28
2.31
Me
O
O
S
Me
9
8
7
6
5
4
3
2
1
ppm
5.70
1.90
2.00
5.97

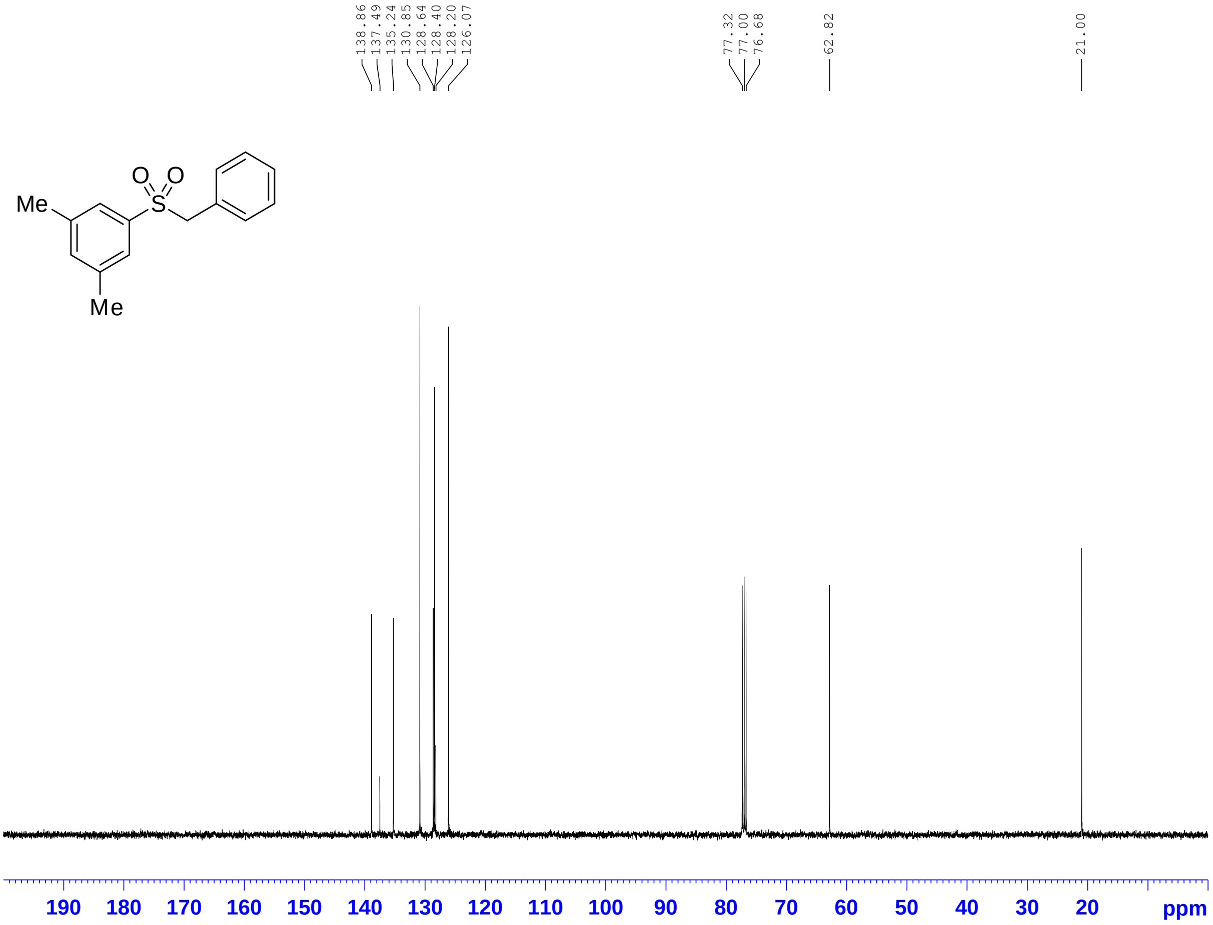
138.86
137.49
135.24
130.85
128.64
128.40
128.20
126.07
77.32
77.00
76.68
62.82
21.00
Me
O
O
S
Me
190
180
170
160
150
140
130
120
110
100
90
80
70
60
50
40
30
20
ppm

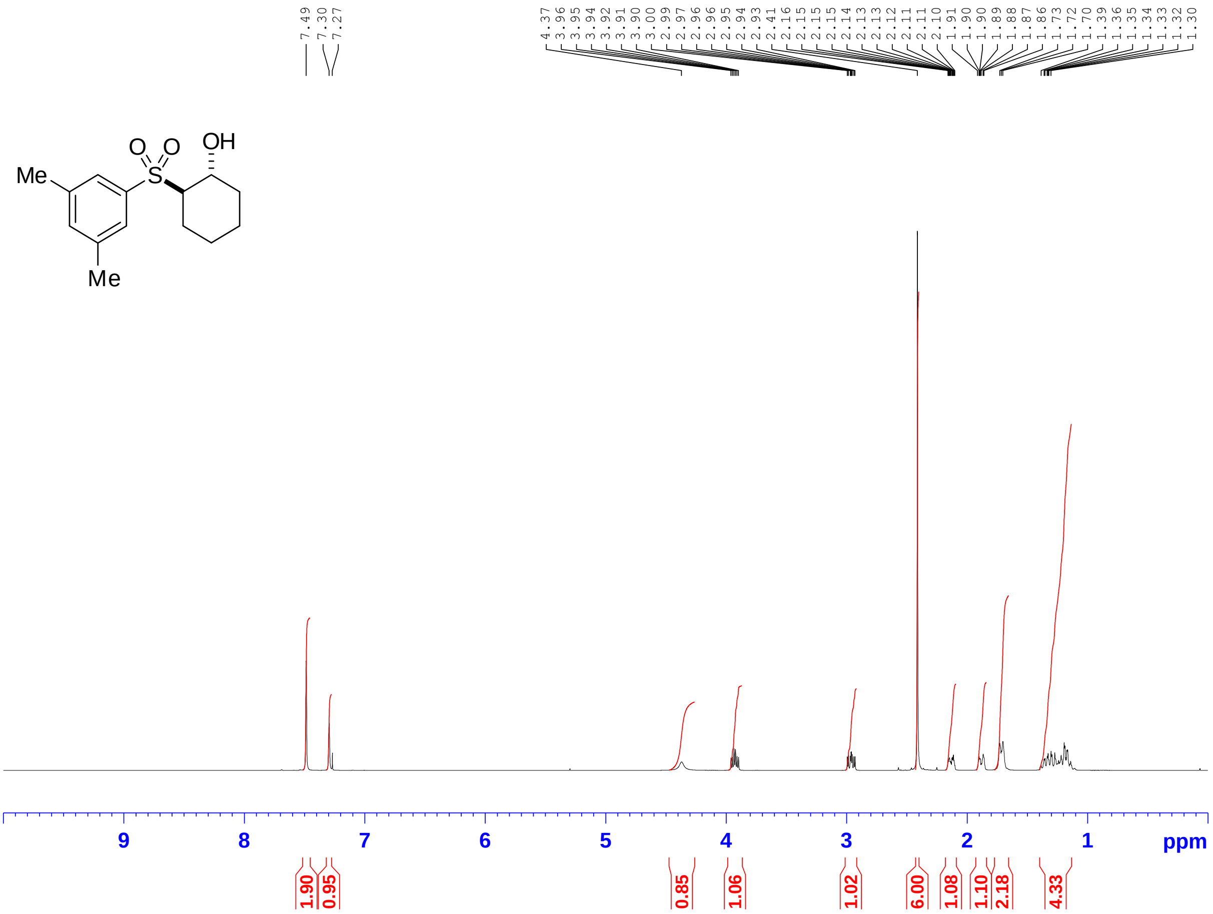
Me
Me
OH
S
O
O
ppm

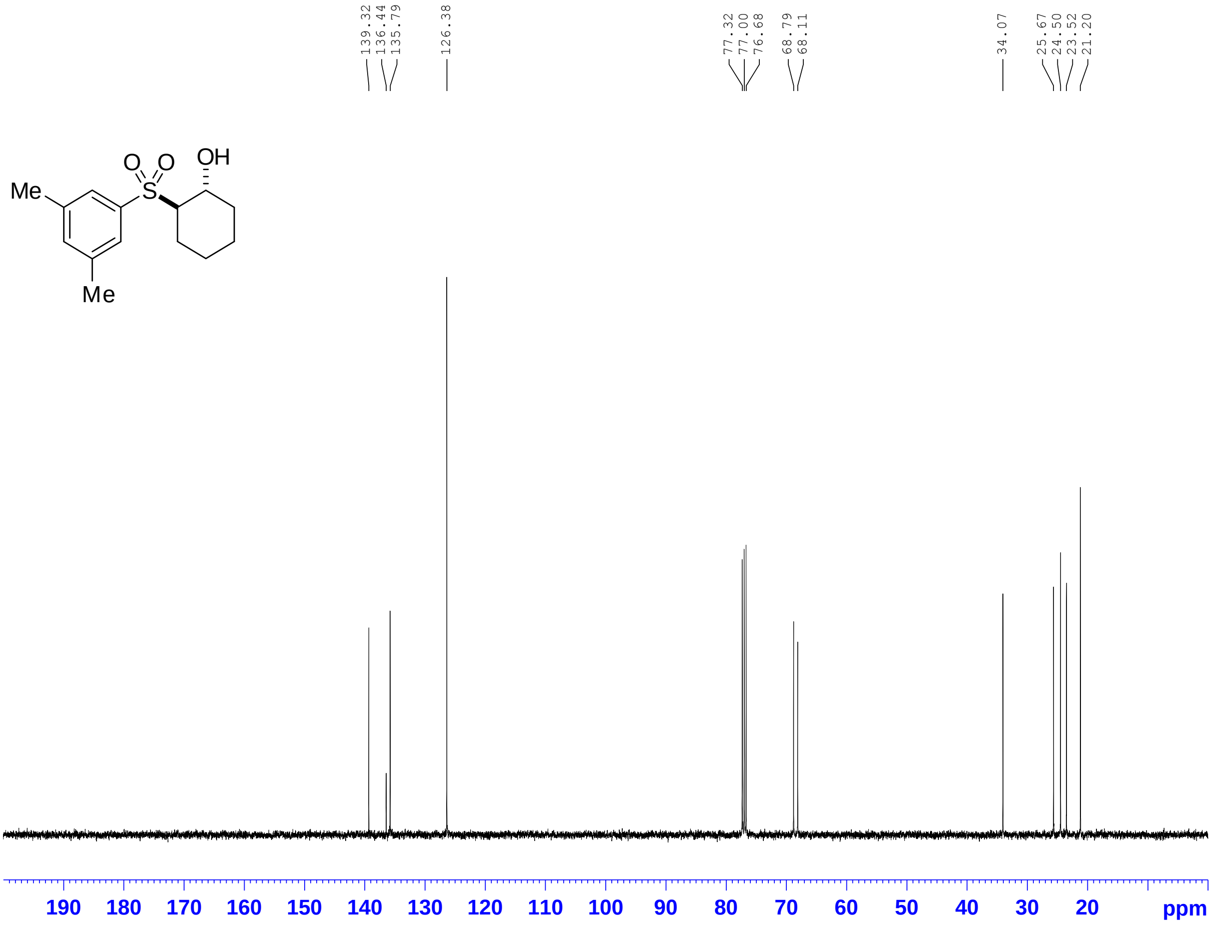
139.32
136.44
135.79
126.38
77.32
77.00
76.68
68.79
68.11
34.07
25.67
24.50
23.52
21.20
Me
Me
OH
S
O
O
ppm

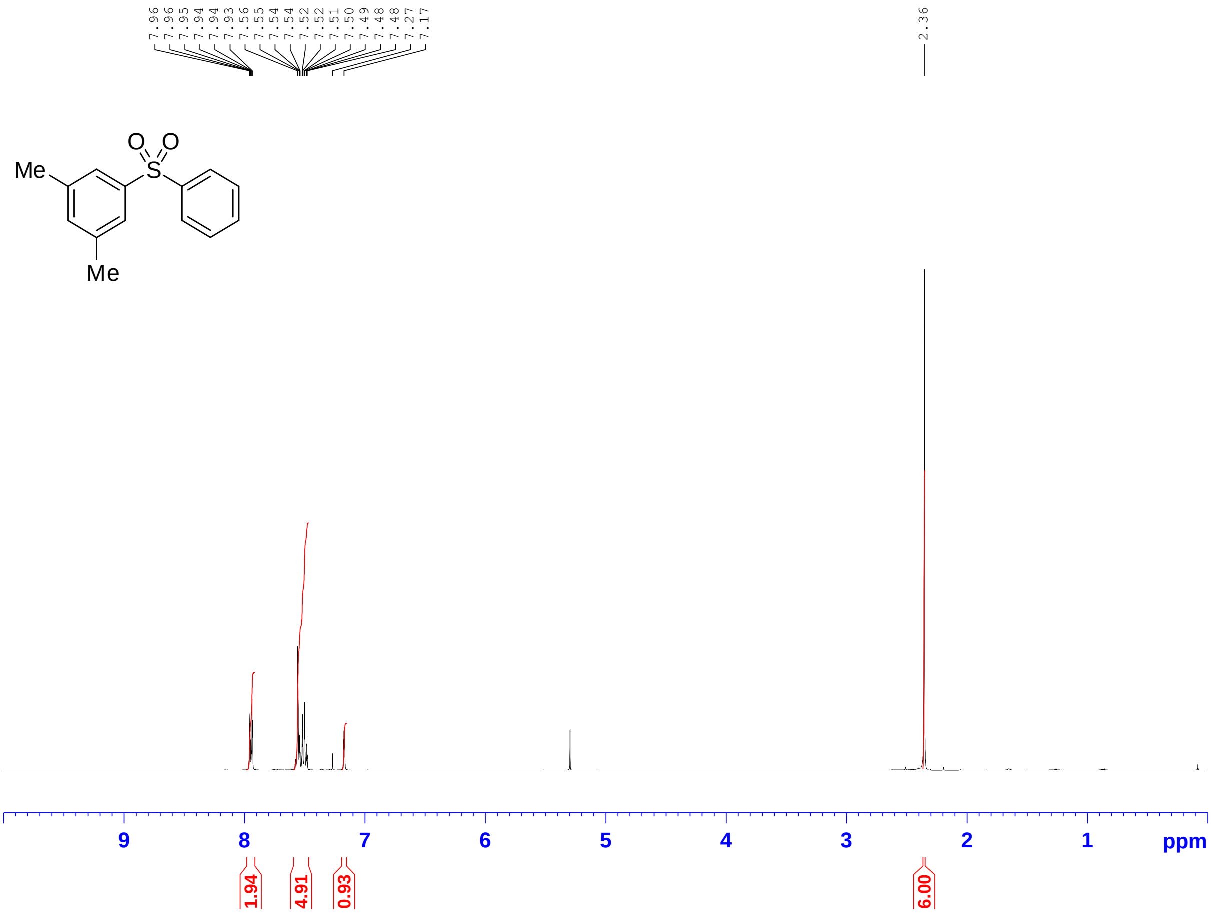
7.96
7.96
7.95
7.94
7.94
7.93
7.56
7.55
7.54
7.54
7.52
7.52
7.51
7.50
7.49
7.48
7.48
7.27
7.17
2.36
Me
Me
O
O
S
1.94
4.91
0.93
6.00
9 8 7 6 5 4 3 2 1 ppm

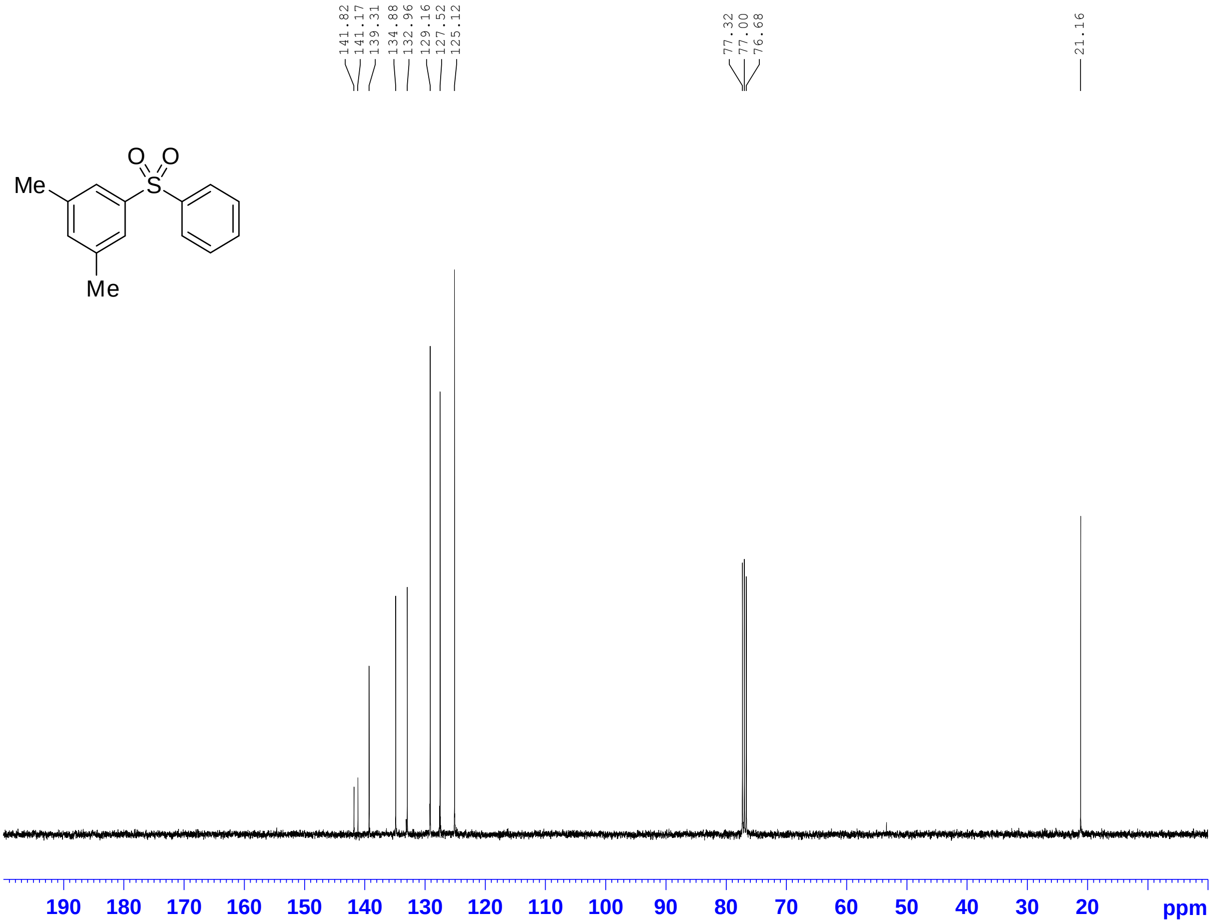
141.82
141.17
139.31
134.88
132.96
129.16
127.52
125.12
77.32
77.00
76.68
21.16
Me
Me
O
O
S
190 180 170 160 150 140 130 120 110 100 90 80 70 60 50 40 30 20 ppm

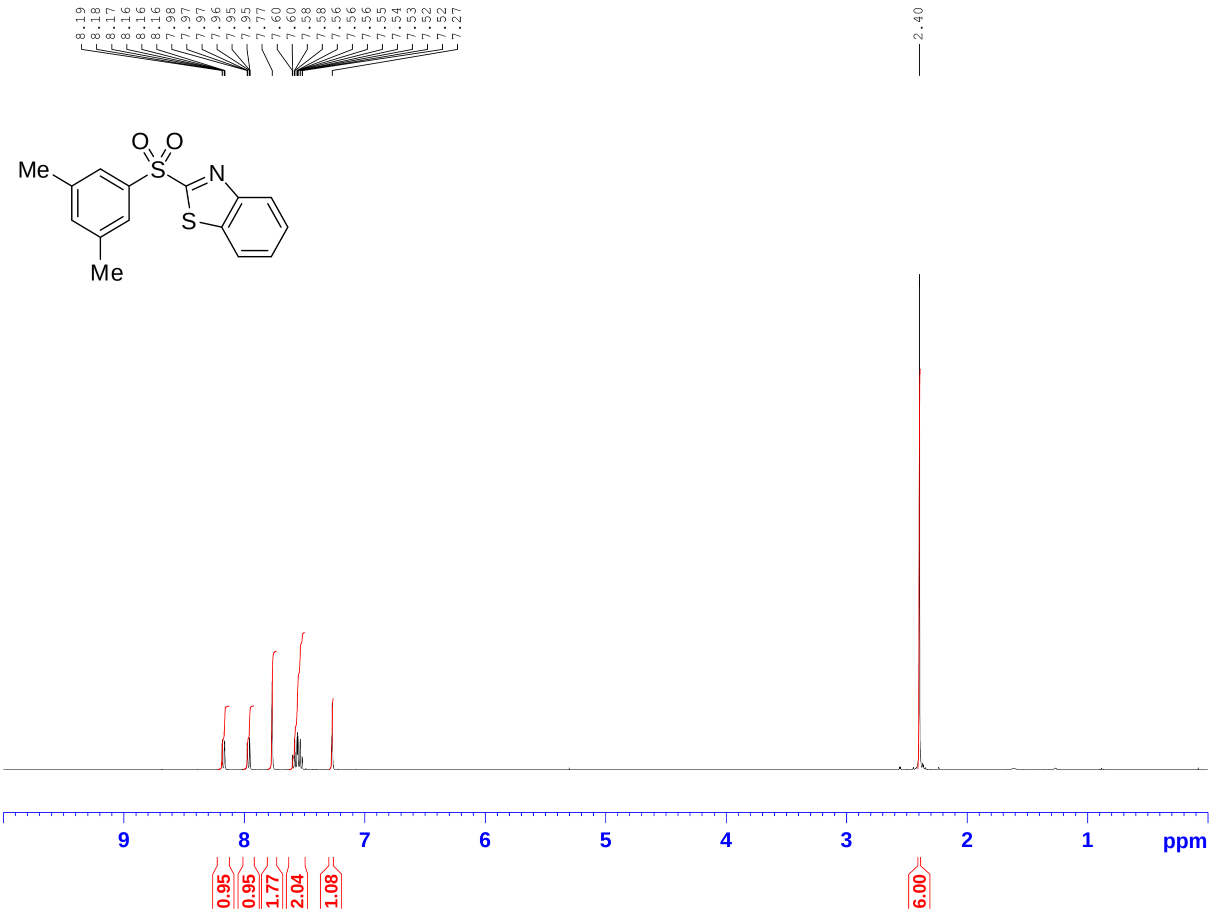
8.19
8.18
8.17
8.16
8.16
8.16
7.98
7.97
7.97
7.96
7.95
7.95
7.77
7.60
7.60
7.58
7.58
7.56
7.56
7.56
7.55
7.54
7.53
7.52
7.52
7.27
2.40
Me
O
O
S
N
S
Me
ppm
0.95
0.95
1.77
2.04
1.08
6.00

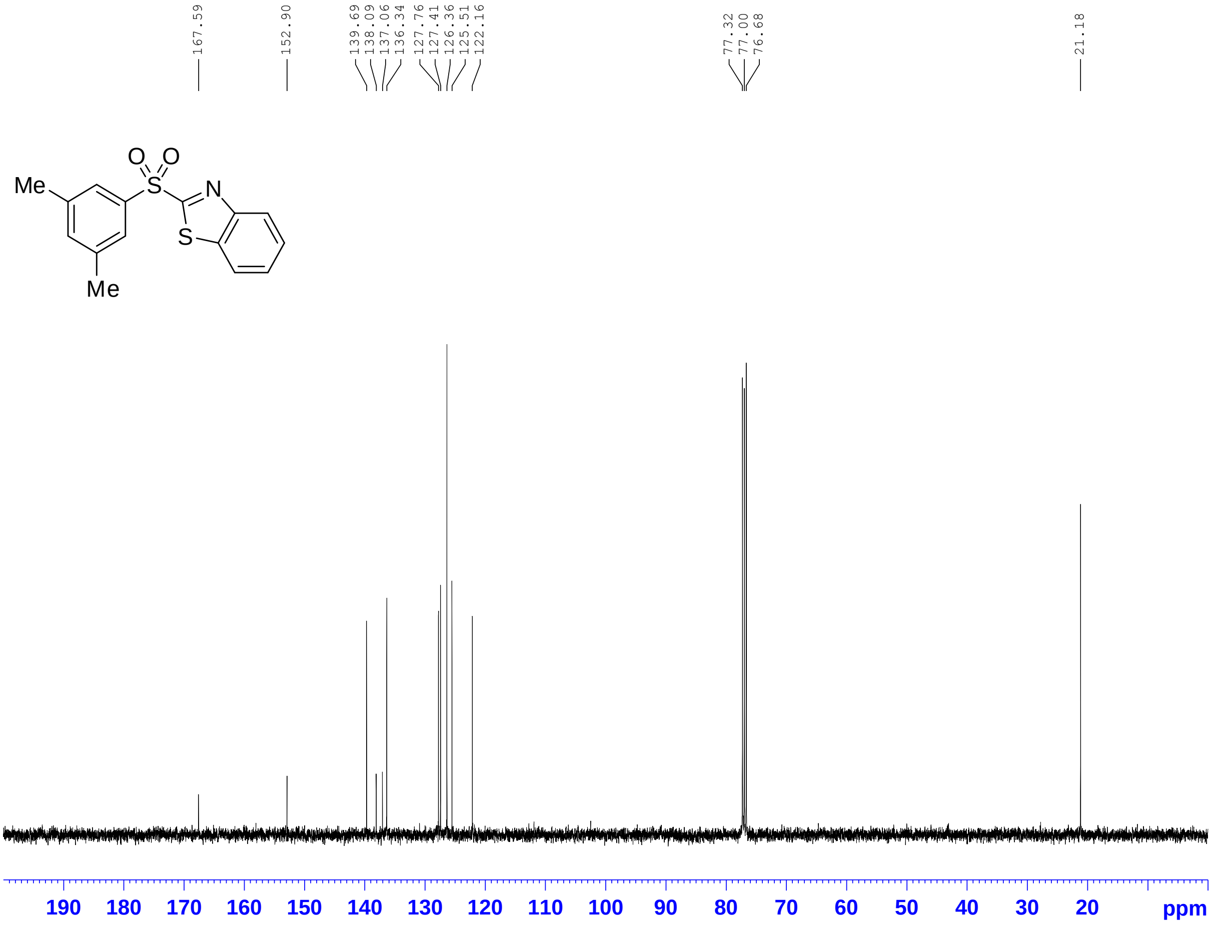
167.59
152.90
139.69
138.09
137.06
136.34
127.76
127.41
126.36
125.51
122.16
77.32
77.00
76.68
21.18
Me
O
O
S
N
S
Me
ppm

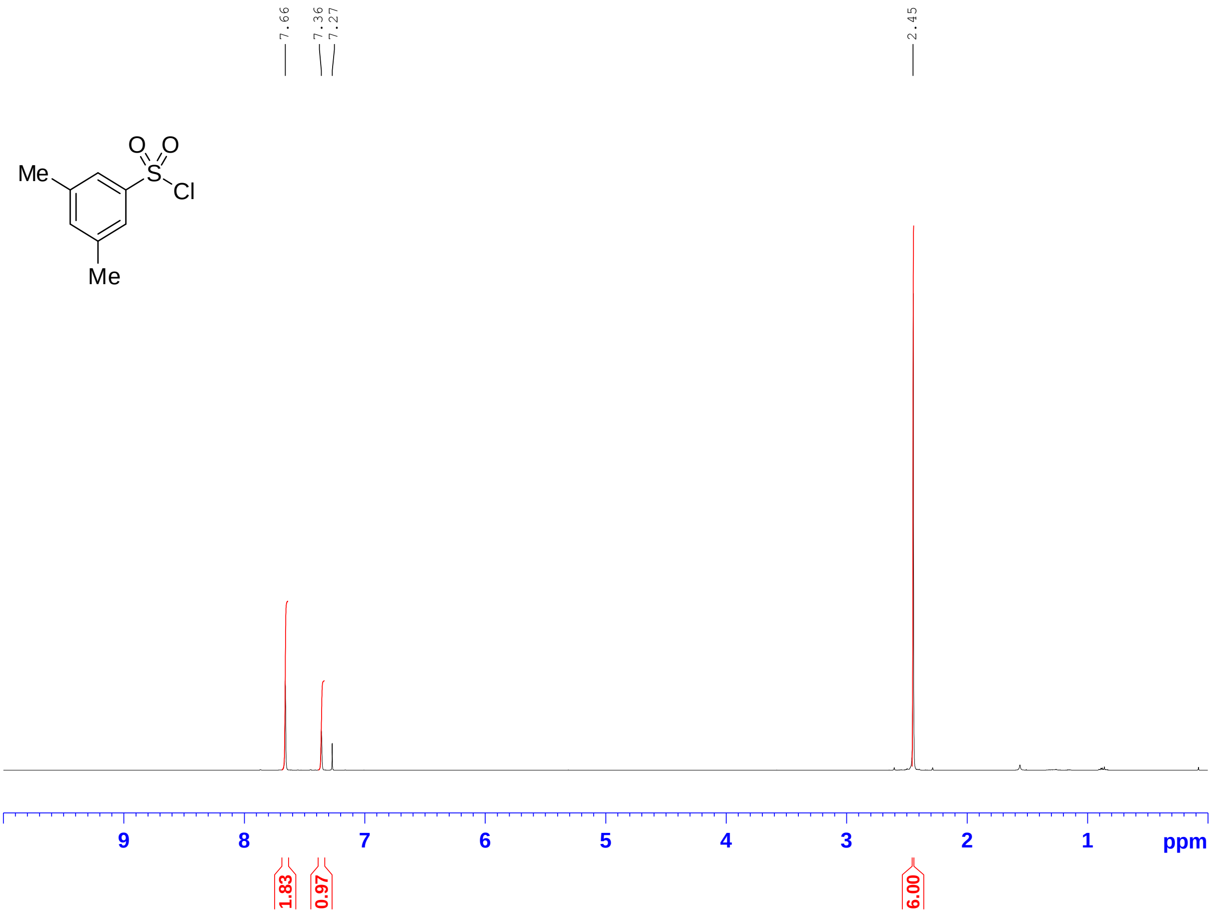
7.66
7.36
7.27
2.45
Me
O
O
S
Cl
Me
9
8
7
6
5
4
3
2
1
ppm
1.83
0.97
6.00

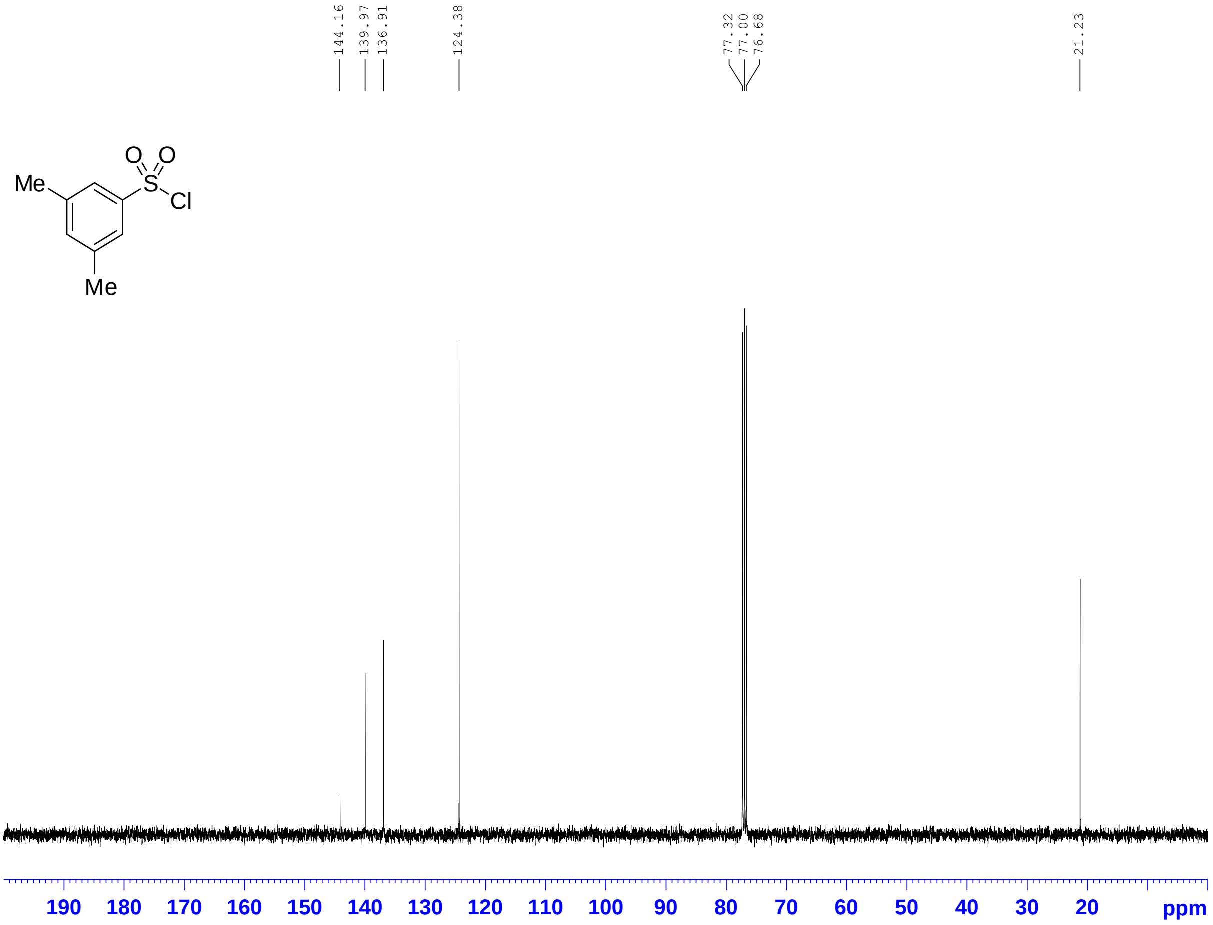
144.16
139.97
136.91
124.38
77.32
77.00
76.68
21.23
Me
O
O
S
Cl
Me
190
180
170
160
150
140
130
120
110
100
90
80
70
60
50
40
30
20
ppm

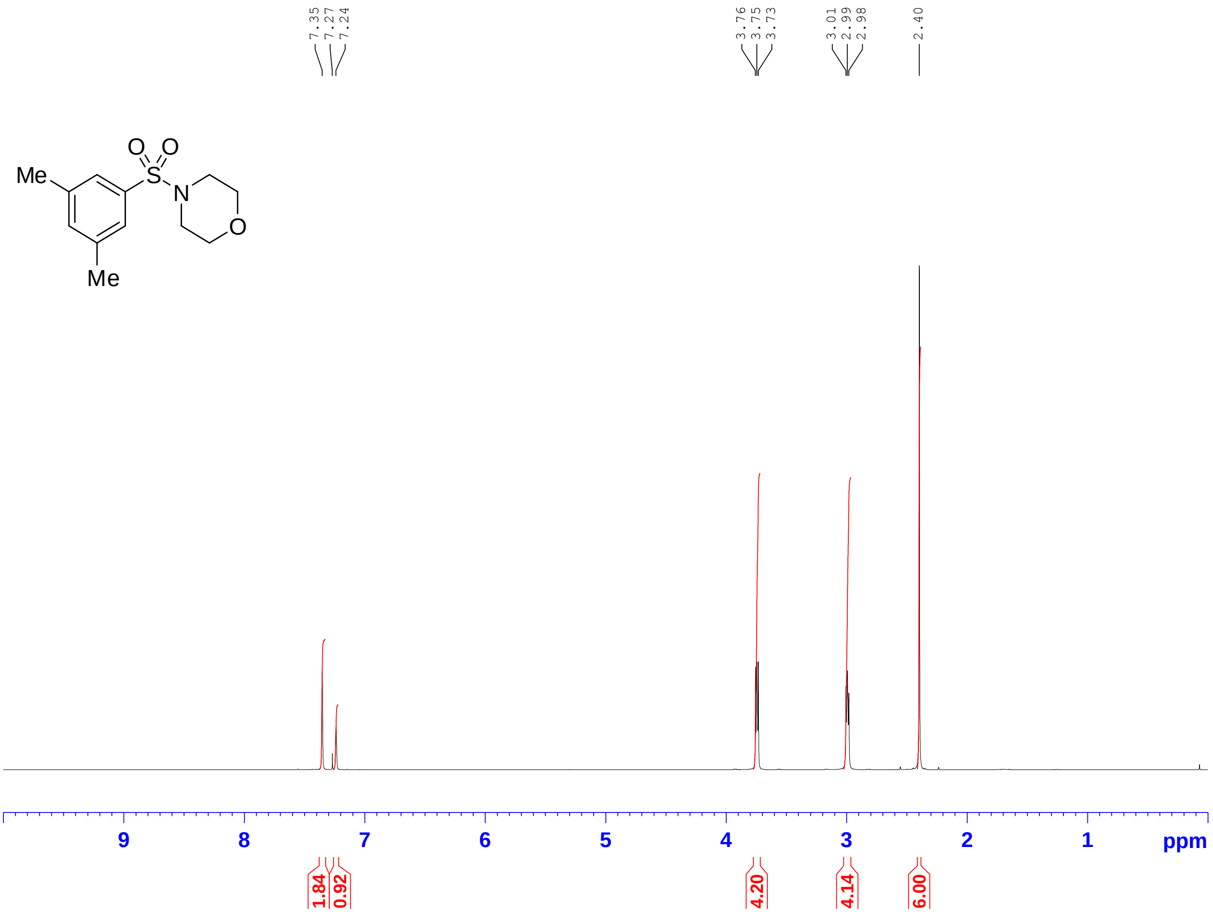
7.35
7.27
7.24
3.76
3.75
3.73
3.01
2.99
2.98
2.40
Me
O
O
S
N
O
Me
9
8
7
6
5
4
3
2
1
ppm
1.84
0.92
4.20
4.14
6.00

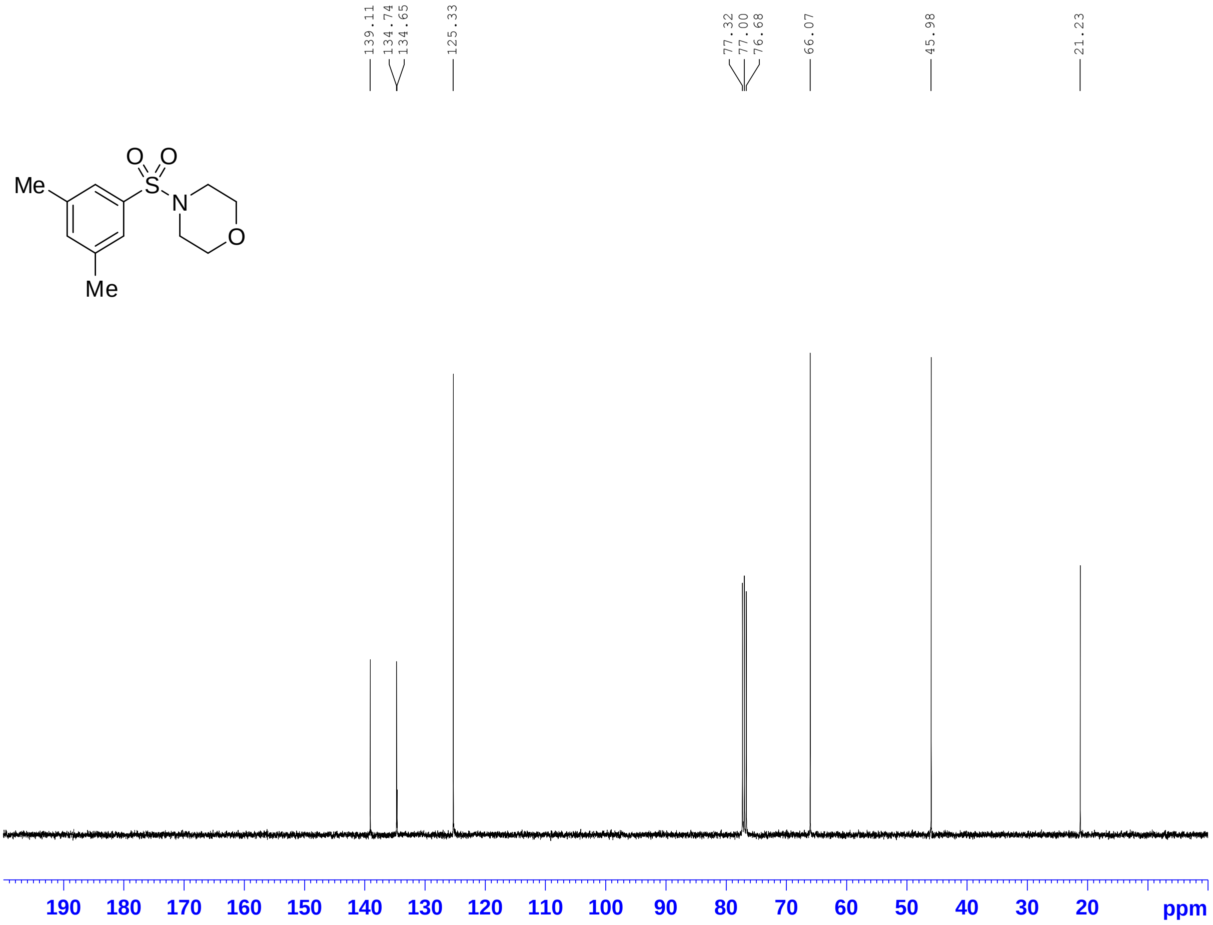
139.11
134.74
134.65
125.33
77.32
77.00
76.68
66.07
45.98
21.23
Me
O
O
S
N
O
Me
190
180
170
160
150
140
130
120
110
100
90
80
70
60
50
40
30
20
ppm

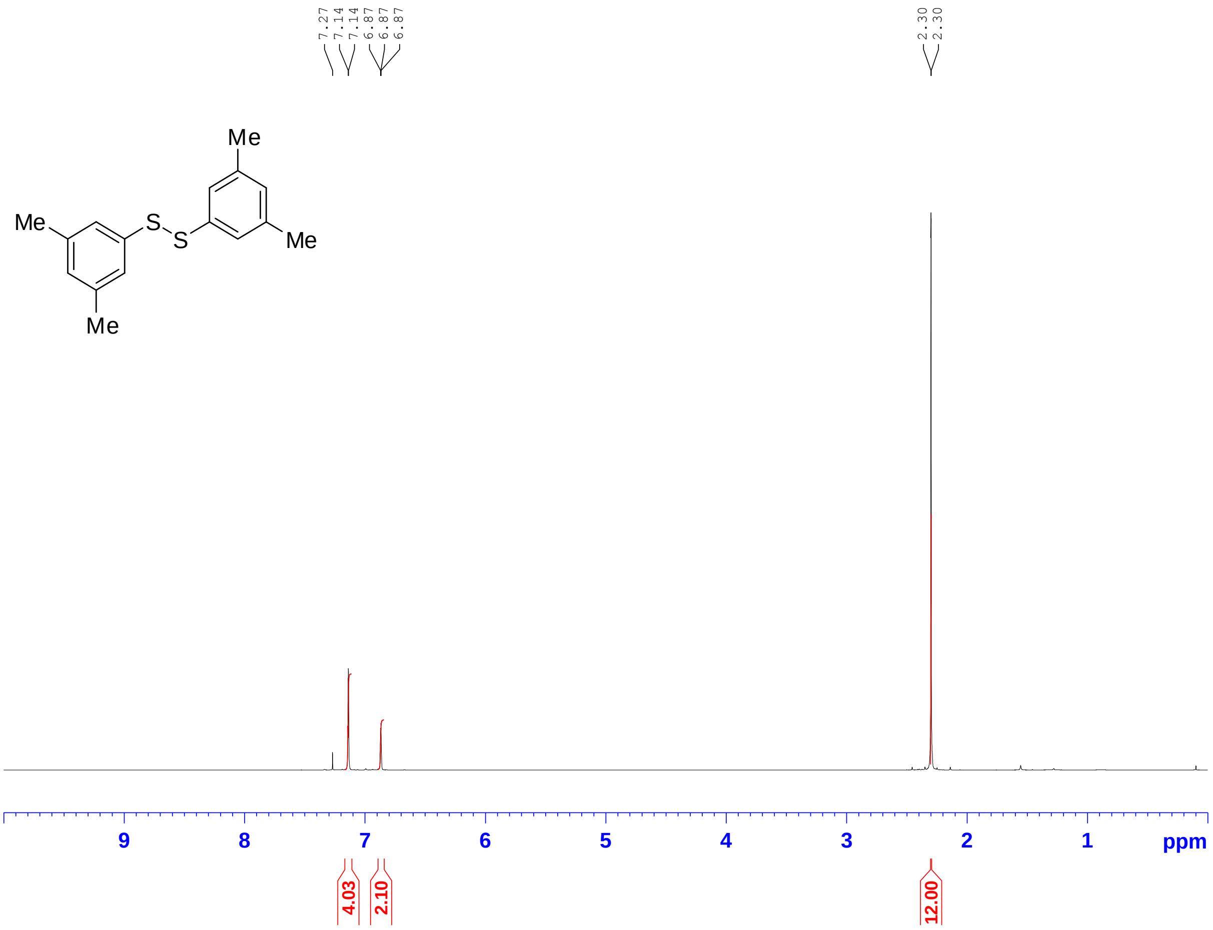
7.27
7.14
7.14
6.87
6.87
6.87
2.30
2.30
Me
Me
S
S
Me
Me
9
8
7
6
5
4
3
2
1
ppm
4.03
2.10
12.00

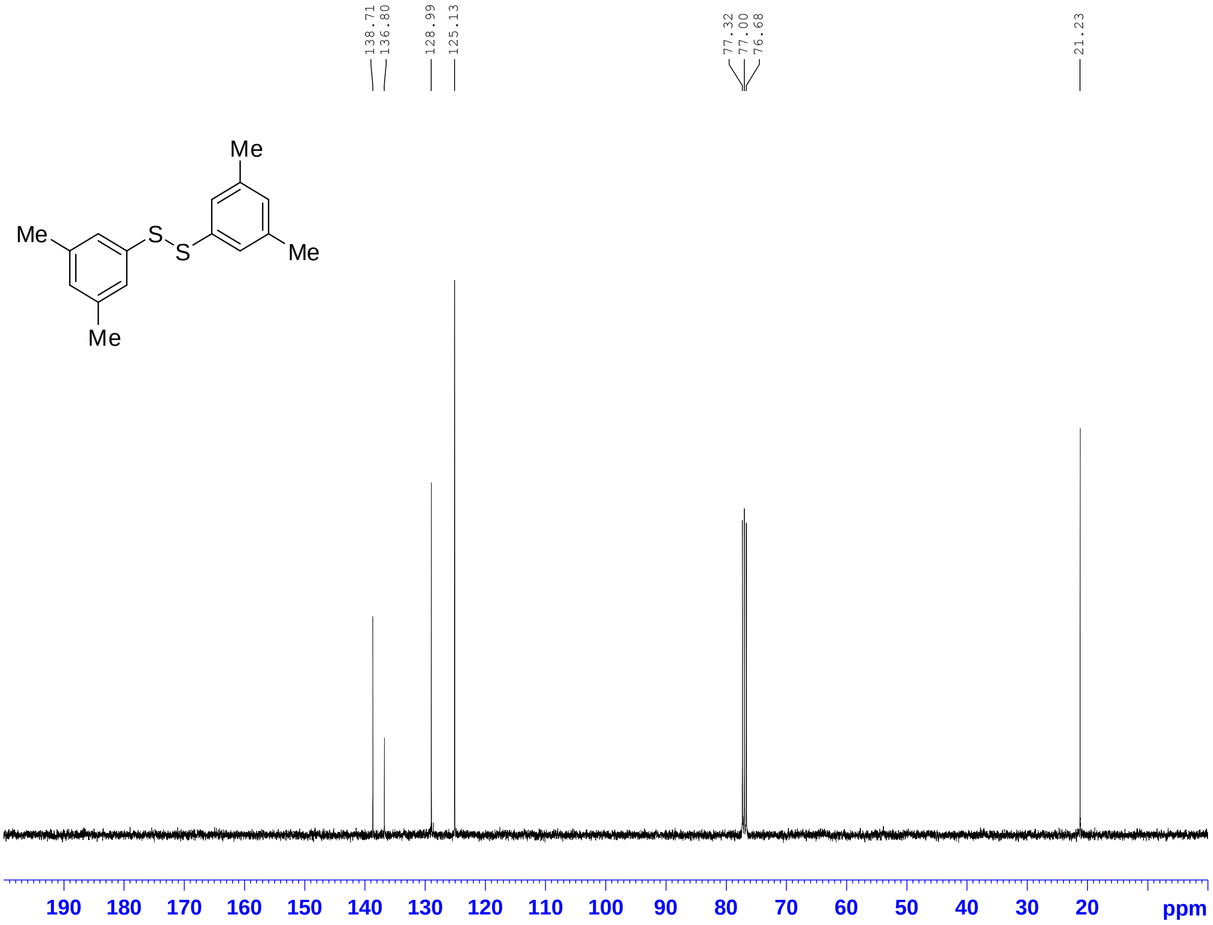
138.71
136.80
128.99
125.13
77.32
77.00
76.68
21.23
Me
Me
S
S
Me
Me
190
180
170
160
150
140
130
120
110
100
90
80
70
60
50
40
30
20
ppm
